# Supplementary material for: Mapping routine measles vaccination in low- and middle-income countries
Source: Nature. 2020 Dec 16;589(7842):415–9. doi: 10.1038/s41586-020-03043-4 (PMC7739806; doi:10.1038/s41586-020-03043-4)
Supplement: Supplementary file 1 — This file contains Supplementary Methods, Supplementary Results and Discussion, Supplementary Figures 1-20 and Supplementary Tables 1-15. [file 41586_2020_3043_MOESM1_ESM.pdf]

nature research
https://doi.org/10.1038/s41586-020-03043-4

# Supplementary information

# Mapping routine measles vaccination in low- and middle-income countries

In the format provided by the authors and unedited

# Mapping routine measles vaccination in low- and middle-income countries


**1.0 Supplementary Methods** 4
*1.1 GATHER compliance* 4
*1.2 Definitions* 4
*1.2.1 Routine first dose of measles-containing vaccine coverage* 4
*1.3.2 Low- and middle-income countries* 5
*1.3 Data* 5
*1.3.2 Data seeking* 5
*1.3.2 Inclusion and exclusion criteria for data sources* 5
*1.3.3 Final list of countries included in analysis* 5
*1.3.4 Data preparation* 5
*1.3.5 Geographic positioning of GPS-located clusters and areal location data* 6
*1.3.6 Geospatial covariate selection* 7
*1.4 Modelling methods* 7
*1.4.1 Geographic modelling regions* 7
*1.4.2 Stacked generalisation child models* 7
*1.4.3 Geospatial model priors* 7
*1.4.4 Spatial mesh construction* 8
*1.4.5 Model fitting* 8
*1.4.6 Additional modelling parameters and limitations* 8
*1.5 Post-estimation calibration and figure production* 9
*1.5.1 Production of GBD coverage estimates* 9
*1.5.2 Figure production* 10
**2.0 Supplementary results and discussion** 10
*2.1 MCV1 coverage* 10
*2.2 Age cohort selection sensitivity analysis* 10
*2.3 Changes in coverage sensitivity analysis* 11
**3.0 Supplementary Figures** 12
*Supplementary Fig. 1:* Household surveys included in modelling 12
*Supplementary Fig. 2:* Data inclusion flow chart 26
*Supplementary Fig. 3:* Areal data spatial resampling 27
*Supplementary Fig. 4:* Geospatial covariates used in modelling 28
*Supplementary Fig. 5:* Example of finite spatial mesh 30

*Supplementary Figs 6–7:* First administrative level in-sample validation plots ........ 31
*Supplementary Figs 8–9:* Second administrative level in-sample validation plots ........ 34
*Supplementary Figs 10–11:* First administrative level out-of-sample validation plots ........ 37
*Supplementary Figs 12–13:* Second administrative level out-of-sample validation plots ........ 39
*Supplementary Fig. 14:* Pixel-level mean, lower, and upper uncertainty limits of MCV1 coverage, 2019 ........ 43
*Supplementary Fig. 15:* First administrative level mean, lower, and upper uncertainty limits of MCV1 coverage, 2019 ........ 44
*Supplementary Fig. 16:* Second administrative level mean, lower, and upper uncertainty limits of MCV1 coverage, 2019 ........ 45
*Supplementary Fig. 17:* District level MCV1 coverage as function of uncertainty via Coffey-Feingold-Bromberg metric, 2019 ........ 46
*Supplementary Fig. 18:* Estimated first-dose measles-containing vaccine (MCV1) coverage among districts in 101 low- and middle-income countries, 2000–2019, full age cohort model. ........ 47
*Supplementary Fig. 19:* Estimated first-dose measles-containing vaccine (MCV1) coverage among districts in 101 low- and middle-income countries, 2000–2019, target age and t+1 cohort model. ........ 48
*Supplementary Fig. 20:* Estimated first-dose measles-containing vaccine (MCV1) coverage among districts in 101 low- and middle-income countries, 2000–2019, target age cohort only model. ........ 49
**4.0 Supplementary Tables** ........ 51
*Supplementary Table 1:* GATHER compliance checklist ........ 51
*Supplementary Table 2:* MCV1 vaccination schedule by country ........ 53
*Supplementary Table 3:* List of countries considered for analysis stratified by Socio-Demographic Index (SDI) level and Decade of Vaccine (DoV) status ........ 57
*Supplementary Table 4:* Input sources included in final analysis ........ 58
*Supplementary Table 5:* Survey sources excluded from analysis ........ 111
*Supplementary Table 6:* Geospatial covariates used in modelling ........ 122
*Supplementary Table 7:* Covariates used per region in stacked generalisation ensemble modelling after variance inflation factor algorithm for covariate selection ........ 127
*Supplementary Table 8:* Fitted model parameters and final penalised control prior specification ........ 129
*Supplementary Table 9a–b:* First administrative level in-sample fit metrics ........ 131
*Supplementary Table 10a–b:* Second administrative level in-sample fit metrics ........ 133
*Supplementary Table 11a–b:* First administrative level out-of-sample fit metrics ........ 135
*Supplementary Table 12a–b:* Second administrative level out-of-sample fit metrics ........ 137
*Supplementary Table 13:* Gini coefficients in 2000 and 2019 ........ 139
*Supplementary Table 14:* Age cohort regression coefficients ........ 145
*Supplementary Table 15:* Key analysis findings for multiple age cohort modelling choices ........ 146
**5.0 Supplementary References** ........ 149

## 1.0 Supplementary Methods

*1.1 GATHER compliance*

Refer to Supplementary Table 1 for information on how this study meets GATHER compliance criteria[1].

*1.2 Definitions*

*1.2.1 Routine first dose of measles-containing vaccine coverage*

Routine coverage of the first dose of any measles-containing vaccine (MCV1), including measles-rubella and measles-mumps-rubella combination vaccines, was defined as the proportion of children born in a given year who received at least one dose of any measles-containing vaccine through routine immunisation services after reaching the target age for immunisation. In keeping with WHO/UNICEF Estimates of National Immunization Coverage (WUENIC) definitions of MCV1 coverage, our MCV1 coverage indicator reflects country-specific population schedules[2]. For countries recommending the first dose of a measles-containing-vaccine for children < 12 months old, the youngest (target age) cohort used to assess coverage in this analysis is 12–23 months old. If the first dose is not recommended until at least 12 months of age, the youngest (target age) cohort used to assess coverage is 24–35 months old. For a full list of schedules by country, please refer to Supplementary Table 2. We have included children among the target age cohort and the cohorts 1 and 2 years older than the target cohort at the time of survey. We excluded children that were 3 years older than the target cohort at the time of survey. We have tested this assumption in a sensitivity analysis that can be found in Supplementary Information section 2.2.

We assign coverage from each birth cohort of children surveyed to the year in which they were aged 0–12 months for modelling, in alignment with the methods used by WUNEIC and estimates from the Global Burden of Diseases, Injuries, and Risk Factors study (GBD). The WUENIC and GBD national-level coverage estimates both additionally incorporate administrative and/or official country-reported data on MCV1 coverage, which does not routinely collect nor report information on the precise birth cohort that accompanies the country-reported values of coverage. In order to maintain comparability with these national-level estimates, and because our estimates are calibrated to those produced by GBD, we adopt the same methodology for birth cohort assignment.

We considered children to have been vaccinated with MCV1 based on evidence from either home-based record (i.e., vaccination card) or parental recall. As with all survey-based measures of vaccination coverage, however, the use of data from parental recall could result in recall bias. Because recall bias of vaccination status varies in both direction and magnitude, we were unable to adjust for this bias[3], a limitation of this analysis.

In order to inform policy decisions regarding measles immunisation strategies, the ability to distinguish between the performance of routine immunisation services and supplemental immunisation activities is key. For instance, SIAs may reach unimmunised children across wealth quintiles more equitably than RI[4], but SIAs may be more vulnerable to disruption due to funding challenges, disasters, or political shifts. Currently, routine immunisation services are still recommended globally in the era of COVID-19[5]04/12/2020 14:14:00 and serve as the primary mechanism through which measles vaccination will be delivered in most settings during the pandemic, given COVID-19-related disruptions to planned SIAs. As the Global Measles and Rubella Strategic Plan suggests, RI services are meant to serve as the cornerstone of measles immunisation in all countries[6].

In this study, we therefore aim to model routine immunisation coverage, within the limits of the available data. To accomplish this aim, we included doses of MCV1 only from routine immunisation wherever possible when processing survey microdata, in order to minimise inclusion of doses administered in supplemental immunisation activities (SIA). See Supplementary Information section 1.3.4 for details on the removal of SIA doses during our data processing. This approach to estimating routine immunisation coverage is concordant with that used in other recent published studies[7] on routine MCV1 coverage. Due to inherent limitations in the underlying survey data, however, some SIA doses are still

likely to be misclassified as routine despite this process – particularly those from recall and from surveys where coding of SIA doses may have been inconsistent. While we aim to produce as representative an estimate of routine MCV1 coverage as possible, estimates presented in this manuscript should be interpreted in the context of this limitation.

*1.3.2 Low- and middle-income countries*

We defined low- and middle-income countries (LMICs) based on Socio-Demographic Index (SDI), as estimated in 2019 by the Global Burden of Diseases, Injuries, and Risk Factors Study (GBD)[8]. This metric combines education, fertility and income to summarise development. A country was included in our analysis if was in the bottom three quintiles and therefore having a low (0–0.455), low-middle (0.455–0.606), or middle (0.606–0.688) classification. Additionally, we included countries if they were part of the 94 Decade of Vaccine priority countries regardless of their SDI classification[8,9,10]. For the full list of included countries (with the exception of Jordan, neither classified as low- or middle-income or a DoV priority country, which was added to increase geographic continuity), refer to Supplementary Table 3.

*1.3 Data*

*1.3.2 Data seeking*

An overview of the entire data preparation and geospatial modelling process can be found in Extended Data Fig. 1. We searched the Global Health Data Exchange[11] (GHDx) for sources with the key word "vaccine," "measles vaccines," "MMR vaccines," "mumps vaccines," "rubella vaccines," "vaccination cards," or "immunisation" for all LMICs . Sources included in models of coverage are listed in Supplementary Fig. 1 and Supplementary Table 4. We gathered a total of 354 surveys for use in final analysis.

*1.3.2 Inclusion and exclusion criteria for data sources*

Data sources considered for inclusion were those for all LMICs containing subnationally-resolved information on MCV1 coverage. Surveys were included if they contained information on children between 12 and 59 months old, precise GPS coordinates or areal geographic boundaries and survey design variables, such as strata and weight.

National-level time series of survey estimates were examined to assess systematic survey bias[2]. When the estimates of a particular source appeared implausible in the context of other data or external information on the reliability of that source, the survey methodology, case definitions, and data collection for that source were evaluated, along with the underlying spatial patterns of the original survey data. Sources assessed as implausible or with known methodological flaws were excluded from the modelling process and are listed in Supplementary Table 5.

*1.3.3 Final list of countries included in analysis*

For 13 countries (Bhutan, Brazil, China, Dominica, Georgia, Grenada, Libya, Oman, Palestine, Saint Lucia, Saint Vincent and the Grenadines, Seychelles, and Venezuela), no available subnational vaccine coverage data met the inclusion and exclusion criteria and therefore have been excluded from this analysis.

*1.3.4 Data preparation*

The following data were extracted from each survey source: vaccine card or home-based record (HBR) doses, parental recall vaccine doses, age (in months), survey weight and design variables, and GPS-cluster or areal location. Individuals with evidence of vaccination either from HBR or recall were considered to

have been vaccinated. Individuals were excluded from the analysis if they were missing age, spatial, or survey design information or were outside of the study age or year range. The study included all years between 2000 and 2019. A comprehensive overview of data from all study geographies included can be found in Supplementary Fig. 2.

Individual age, in months, at the time of survey collection was used to assign each child to a birth cohort (12–23 months, 24–35 months, 36–47 months, and 48–59 months). Data corresponding to each birth cohort were included in the modelling process in the year in which that birth cohort was aged 0–12 months old[12]. This method allows the inclusion of additional individuals, which increases overall geographical representation, but requires assumptions such as negligible catch-up vaccination and no differential mortality or migration. However, the influence of including older cohorts in our model on the key findings appeared to be minor (Supplementary Information section 2.2).

Wherever possible, we removed doses recorded on vaccination cards or from parental recall that were attributable to SIAs. First, we reviewed the underlying survey methodology to identify, where applicable, the coding used to identify doses delivered through SIAs rather than routine immunisation services. We confirmed the occurrence of each SIA by cross-referencing the summary documentation of historical SIAs compiled by WHO[13]. For children who participated in a SIA and had card or recall codes that indicating doses which were attributable to the campaign, we excluded those doses from our analysis of routine immunisation coverage. For surveys for which SIA doses were not identified through coding on vaccination cards or in specific recall survey variables, we were not able to make this distinction, reflecting an inherent limitation in data collection and recording from the underlying survey data.

*1.3.5 Geographic positioning of GPS-located clusters and areal location data*

Individuals were assigned to the most granular geographic location possible. Antigen-dose-specific coverage at each of these locations, either GPS-located cluster or an areal location, was calculated. The mean MCV1 coverage was calculated at the most precise geographic location possible. Survey weights were taken into account using the Kish adjustment when calculating averages for areally assigned data. Data without GPS information were matched to the most precise geographic location possible using a library of boundary definitions largely composed of shapefiles from individual surveys, the Global Administrative Unit Layers database, the Database of Global Administrative Areas, and other country-specific shapefiles. If the smallest geographic unit available was smaller than a 5 × 5-km pixel, the unit was assigned to a GPS-located cluster at the centroid of the pixel.

To be included in the model-based geostatistical framework, all data must be geo-located at the precision of a specific latitude and longitude coordinate pair. For areally located data that has been matched to an administrative unit but for which no GPS coordinates were available, we used a previously described method to generate a set of candidate cluster locations where survey sampling could plausibly have occurred, using population weights and k-means clustering[14] (Supplementary Fig. 3). In brief, 10,000 points were randomly sampled from the area within the administrative boundary that was matched to the areal data. Points were sampled proportionally to the total population within that space and time as estimated by the WorldPop population raster[15]. A set of integration points (1 per 1,000 5 × 5-km pixels) was generated via k-means clustering, representing candidate locations of survey sampling under the assumption that each survey sampled locations proportional to population. These integration points were used as input locations for the areal data in the geospatial model. Each integration point was assigned the mean MCV1 coverage value previously calculated for the entire areal location. A weight was also assigned to each integration point (i.e., candidate observation location) that is proportional to the number of randomly sampled points that were included in the k-means cluster, such that all weights sum to 1 for each areal data observation. The “survey” package in R[16] was used to determine a conservative sample size for the entire areal unit, and the sample size at each integration point was determined using this calculated total sample size and the weights of each integration point. Overall, this method produces a set of candidate observations of vaccine coverage spread over the area in question, with increased weight given to locations that are more densely populated.

*1.3.6 Geospatial covariate selection*

We used a variance inflation factor (VIF) algorithm for geospatial covariate selection before using the subsequent covariates in child models. We chose to use a covariate selection algorithm before fitting child models to remove collinear covariates to facilitate model convergence. For each coordinate point where data is located, geospatial covariate values were extracted. The VIF algorithm works by computing the variance of each covariate, which is conflated when multicollinearities exist in the covariate values[17]. The VIF was computed for each covariate, removing the largest incrementally, until the VIF for each remaining covariate was less than 3. This algorithm was run for all modelling regions (as discussed in Supplementary Information section 1.4.1).

## *1.4 Modelling methods*

*1.4.1 Geographic modelling regions*

Coverage was modelled in 13 continuous geographic regions, and the outputs of these regional models were combined to create comprehensive maps of antigen-dose-specific coverage in LMICs. Countries were assigned to one of 13 regions. Regions were decided by adapting regions defined by GBD, which are constructed to group countries together by epidemiological similarity and geographical proximity (Extended Data Fig. 2). We modified these regions to ensure geographical contiguity. We included a one-degree buffer to each region when modelling to ensure minimal effects of regional edges in final results. Computationally, it is much more efficient to fit multiple regions in parallel that are smaller in area. Additionally, fitting models in separate regions allows for different regional relationships in coverage with geostatistical covariates.

*1.4.2 Stacked generalisation child models*

Stacked generalisation is an ensemble method for combining models and has shown to increase predictive accuracy in spatiotemporal disease modelling[18]. We use stacked generalisation in our modelling framework to combine predictions of our child models (lasso, GAM and boosted regression trees) for three main reasons. First, its use allows us to increase our predictive accuracy, although they reduce the causal interpretation of the subsequent results. Next, as not many geospatial covariates largely explain vaccination coverage, stacked generalisation maximise the predictive power of the covariates we do have. Third, staked generalisation allows us to account for the complex, non-linear relationships between covariates and outcomes in our model.

The choice of using stacked generalisation breaks the ability for our model to be used to describe any causal pathway. Ideally, models would be optimised for prediction and causal inference. However, as our objective is predictive accuracy rather than causal inference, we believe that the benefits of inclusion of a stacked generalisation step outweigh this limitation.

*1.4.3 Geospatial model priors*

The following priors were used for modelling MCV1 coverage:

$$\beta_0 \sim \mathrm{N}(\mu = 0, \sigma^2 = 3^2),$$
$$\beta_h \sim^{iid} \mathrm{N}(\mu = 0, \sigma^2 = 3^2),$$
$$\log\left(\frac{1+\rho}{1-\rho}\right) \sim \mathrm{N}(\mu = 0, \sigma^2 = 1/0.15),$$
$$\frac{1}{\sigma^2_{nugget}} \sim \mathrm{loggamma}(\alpha = 1, \gamma = 2),$$
$$\frac{1}{\sigma^2_{ctry(i)}} \sim \mathrm{loggamma}(\alpha = 1, \gamma = 2),$$

For the Matérn function, we used a penalised complexity prior for the range, δ, such that $P(\delta < \boldsymbol{\delta_0}) < 0.05$, and standard deviation, $\sigma_{space}^2$, such that $P\left(\sigma_{space}^2 > 3\right) = 0.05$. $\boldsymbol{\delta_0}$ was chosen such that it was ~ 5% of the region size[19].

*1.4.4 Spatial mesh construction*

The SPDE approximation to the residuals of the Gaussian process requires the construction of a finite spatial elements mesh for each modelling region in order to fit the spatiotemporal correlated error term in the generalised linear modelling framework. A spherical finite mesh was constructed for each modelling region using edge-smoothed polygon boundaries; the inner mesh triangle edge length ranges from 25 to 500 km and the outer mesh edge length is 1000 km. For an example mesh, see Supplementary Fig. 5. With this type of spatial mesh, the spatial distance matrix as part of the Matérn covariate, was calculated using the greatest circle distance along the surface of the sphere (i.e. Earth).

We define the SPDE on $\mathbb{S}^2$, a spherical manifold, and use the great circle distance as described in the SPDE framework. Practically, this is implemented in our modelling pipeline by first converting data locations and geographic region boundaries to three-dimensional coordinates on the unit-sphere. Next, using the INLA "inla.mesh.create" function, we construct an $\mathbb{S}^2$, mesh using the three-dimensional data and boundaries.

*1.4.5 Model fitting*

Models were fit using R-INLA as described previously[14]. We have chosen to use R-INLA for model fitting and interpretation to leverage its computational tractability when running models across large spatial scales. This is possible through maximising computational efficiency via an inherent parallelising structure to exploit multi-core processing and also generalising across latent Gaussian models which allows for a significant amount of inference to be automated.

Cluster-level observations for which precise latitude and longitudinal coordinates are known were assigned a weight of 1. Areal data that was resampled as described above were given weight as a function of the K-means clustering process. These weights were used in the R-INLA fitting process to ensure both observation types were given equal contribution to the log-likelihood function of the model.

During model fitting, we apply a linear sum to 1 constraint on the child model $\beta_c$'s using the "extraconstr" argument of R-INLA[20], such that:

$$A\boldsymbol{\beta}_{\mathbf{c}}^{\top} = \mathbf{e}^{\top}$$

where A is a matrix of dimensions (1,1, length of vector of the fixed effects (i.e. 3)) and e = 1.

Model fitting parameters can be found in Supplementary Table 8. The posterior is numerically approximated as part of the INLA approach and thus convergence cannot be assessed in a comparable way as with a full MCMC sampler. As an alternative, we used the R-INLA "inla_results$mode$mode.status" function to ensure that the INLA results have identified the posterior modes of the hyperparameters and the associated Hessian is positive definite. This check was passed for all modelled regions.

*1.4.6 Additional modelling parameters and limitations*

We modeled using AR(1) temporal structure; alternative approaches could provide more flexibility, but in practice we find that this works adequately given subsequent out-of-sample model performance. Selection of optimal spatiotemporal covariance structures for vaccine coverage modeling is an important area for further investigation in future work. Also, we used a model that is separable in space and time. While this assumes that the covariance between years and locations has the same functional form regardless of the locations. Fitting a non-separable model would be complex and computationally challenging and should also be considered in future work.

*1.5 Post-estimation calibration and figure production*

*1.5.1 Production of GBD coverage estimates*

Using the results of the geostatistical modelling process above, we calibrated our set of posterior draws to estimates of MCV1 coverage from GBD 2019[8]. The geostatistical model described above uses only subnationally-resolved survey data, while coverage estimates produced by GBD 2019 additionally leverage surveys for which only national-level data are available as well as bias-adjusted national-level administrative data. This calibration step allows our geospatial estimates to reflect national-level trends in coverage that are informed by national-level surveys and administrative data, while preserving the relative spatial patterns estimated by the geospatial model. GBD 2019 produced coverage estimates for 11 vaccine antigen-dose combinations in 204 national and 858 subnational locations from 21 countries from 1980 to 2019. Coverage indicators represent the same indicators as in the geospatial models, which is the proportion of children in the target age population receiving MCV1, as previously described.

Data from household surveys, administrative reports of coverage, and other survey reports and tabulations are used to produce GBD coverage estimates. The GHDx and targeted country searches were used to identify all nationally-representative sources containing information on vaccine coverage. Household survey series used include the Demographic Health Survey (DHS) series, Multiple Indicator Cluster Survey (MICS) series, Reproductive Health Survey (RHS) series, and Living Standards Measurement Study (LSMS) series. Appropriate survey weights were applied to generate weighted national estimates of coverage, and accompanying uncertainty was calculated from standard error and sample size.

Annual estimates of administrative immunisation coverage from the Joint Reporting Form (JRF) were collected. GBD applies a time-varying country- and vaccine-specific bias adjustment to account for changing degrees of bias in the JRF-reported administrative estimates over time[8]. The ratio of survey coverage to administrative estimates for paired points in the same year and location was modelled in a spatiotemporal Gaussian process regression (ST-GPR) using SDI as a predictive covariate. From this model, estimates of administrative bias were obtained for all locations and years, which were then used to adjust the administrative data prior to inclusion in the final vaccine coverage model.

GBD uses an ST-GPR model to estimate vaccination coverage by synthesising data from various sources while borrowing strength from data across time and space. This method produces a complete time series and accompanying uncertainty for each location and antigen-dose combination and has been widely used in many other GBD risk factors and causes. The ST-GPR model mean function can be summarised as:

$$m_c(t) = X\beta + h(r_{c,t}) ,$$

where $X\beta$ is a linear model and $h(r_{c,t})$ is a residual smoothing function, for each country $c$ and each year $t$. In estimating vaccine coverage using ST-GPR, the following linear model was used:

$$logit(P_{c,t}) = \beta_0 + \beta_1 HAQI_{c,t} + \beta_2 war_{c,t} + \beta_3 stockout_{c,t} + \alpha_c + \gamma_{R[c]} + \omega_{SR[c]} + \varepsilon_{c,t} ,$$

where $P_{c,t}$ is vaccination coverage, $HAQI_{c,t}$ is health access and quality index[21], $war_{c,t}$ is mortality rate due to war and terror, and $stockout_{c,t}$ is a variable representing the magnitude of a stock-out as estimated by the logit-space difference between observed and expected administrative coverage for locations and years with a known antigen-specific vaccine stock-out, for each country $c$ and each year $t$. $\alpha_c$ , $\gamma_{R[c]}$, and $\omega_{SR[c]}$ are country, GBD region, and GBD super-region random effects. Estimates were produced through ST-GPR regression steps with 1,000 random samples from the posterior distribution per country and year. Uncertainty intervals were taken from the 25th and 975th ordered posterior samples.

*1.5.2 Figure production*
All maps were generated using ArcMap 10.6 and Python 2.7[22,23]. Other figures were produced using R version 3.6.1[24].

## 2.0 Supplementary results and discussion

*2.1 MCV1 coverage*
We aggregated estimates to country and first and second administrative boundaries, and showed pixel-level maps for years 2000, 2005, 2010, 2015, and 2019 (Extended Data Figs 3-7). In 2019, pixel-level means and 95% uncertainty intervals for the full posterior distribution are shown in Supplementary Figs 14-16. These results for all years 2000 to 2019 are also available in the online visualisation tool, which can be found here: (https://vizhub.healthdata.org/lbd/mcv).

Additionally, to portray uncertainty, the Coffey-Feingold-Bromberg statistic[25] was used. This statistic provides a measure of the uncertainty in a set of draws of proportions that is comparable across different means and ranges from 0% indicating no uncertainty and 100% indicating highest possible uncertainty (Supplementary Fig. 17).

As the relationships between covariates and coverage are multi-dimensional, we used methods to account for these complex relationships. These methods are optimised for prediction, not inference, and this model therefore cannot be used to infer a causal relationship between any given covariate and vaccination coverage.

*2.2 Age cohort selection sensitivity analysis*

In this analysis, we include data from multiple age cohorts of surveyed children where available, including the target age cohort and older birth cohorts. Older children may be more likely to be vaccinated due to additional opportunities for vaccination, e.g. through catch-up routine immunisation services, inclusion in additional supplemental immunisation activities, or through a second opportunity for first-dose immunisation during the second year of life in countries that have introduced MCV2. Alternatively, older cohorts may have lower coverage in some cases than younger cohorts in a given survey, due to – for instance – rapidly increasing trends in immunisation coverage. The inclusion of older children in the model, therefore, has the potential to introduce bias. If this bias is negligible, however, exclusion of older children would remove potentially informative observations of coverage across space and time from the model. To determine which cohorts of children should be included in our model, we therefore conducted a series of regression and sensitivity analyses to assess for potential bias and evaluate the influence of these decisions on the key findings of this study.

First, we conducted an analysis of cohorts of children for which we have multiple observations of vaccine coverage from surveys conducted at different points in time. We chose this approach because simply looking at coverage by cohort within a given survey presents an age-period-cohort problem, where the effects of changing coverage by age and changing coverage within a country over time are difficult to disentangle. By analysing cohorts with multiple survey observations, we could compare survey estimates of coverage for the cohort obtained at the time in which they were the target age and in at least one other year, for instance when the children were one year older than the target age (t+1), two years older than the target age (t+2), or three years older than the target age (t+3). We evaluated the effect of the time lag between survey observations for a given birth cohort using the following regression analysis, which yielded the results shown in Supplementary Information Table 14:

$$\ln\left(\frac{coverage\ observed\ when\ cohort\ was\ older\ than\ target\ age}{coverage\ observed\ when\ cohort\ was\ of\ the\ target\ age}\right) = I_{t+1}\beta_1 + I_{t+2}\beta_2 + I_{t+3}\beta_3$$

There was a significant effect for the indicator variable for the largest (t+3) time lag between observations for a given cohort. Therefore, these oldest cohorts, i.e. those that were 3 years older than the target age group, were removed from our data set.

However, to ensure major themes of this work were not sensitive to choice in age cohort, we ran three additional versions of the models: a model including all cohorts from each survey, a model with the target age cohort along with t+1 and t+2 cohorts (the approach presented in the main text, figures, and tables), a model with only the target age cohort and the t+1 cohort, and a model with only the target age cohort. The results and major findings of this work did not sustainably change among the four cohort versions (Supplementary Table 15, Supplementary Figs. 18-20).

*2.3 Changes in coverage sensitivity analysis*

If a district has high starting coverage, there is less room for improvement in coverage and a higher likelihood of stagnant coverage values. Therefore, we performed a sensitivity analysis of changes in coverage between 2000 to 2010 and 2010 to 2019 to account for baseline starting value. Including only districts with coverage below 90% in 2000, 82.2% (95% Uncertainty Interval (UI): 77.6–86.1%) of districts had increasing coverage from 2000 to 2010. Including only districts with coverage below 90% in 2010, 44.6% (95% UI: 38.3–53.4%) of districts had increasing coverage from 2010 to 2019. Our original trend presented therefore holds true in this supplementary analysis – coverage improved in fewer districts between 2010–2019 when compared to the time period 2000–2010.

## 3.0 Supplementary Figures

*Supplementary Fig. 1:* Household surveys included in modelling
All data are shown mapped to their corresponding geographic area. This is prior to candidate points being generated for areal data. The sample size represents the number of individual children included in the underlying microdata record for each survey. From 2000 to 2019, MCV1 data availability is shown for **a)** Caribbean, **b)** Central America, **c)** Central Asia and Eastern Europe, **d)** Central sub-Saharan Africa, **e)** East Asia, **f)** Eastern sub-Saharan Africa, **g)** North Africa and Middle East, **h)** Oceania, **i)** South America (Andean), **j)** South America (Tropical), **k)** South Asia, **l)** Southern sub-Saharan Africa, and **m)** Western sub-Saharan Africa. Countries excluded from this analysis due to lack of reliable survey data are shown in grey.

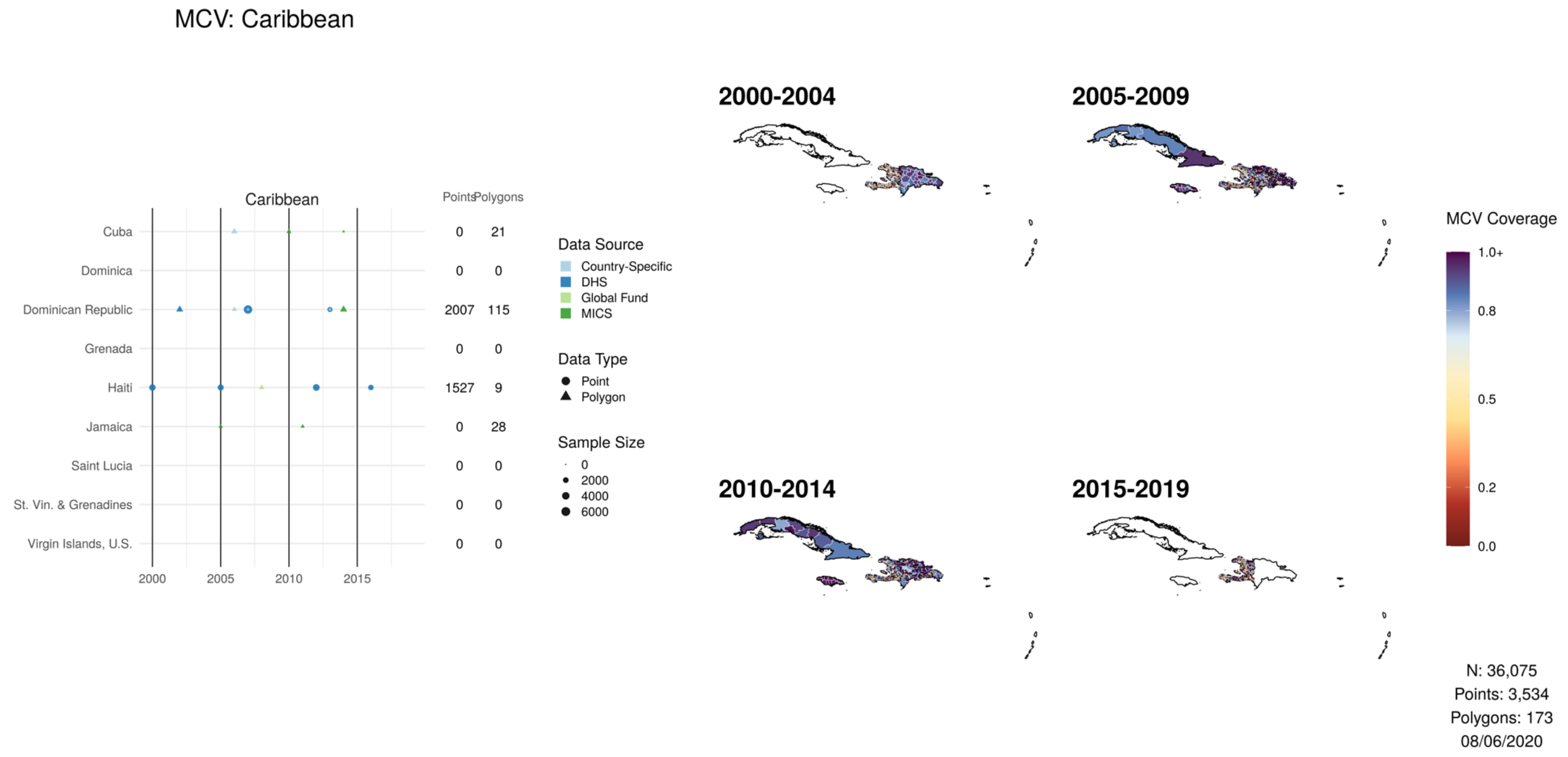


*Supplementary Fig. 1a.* Caribbean

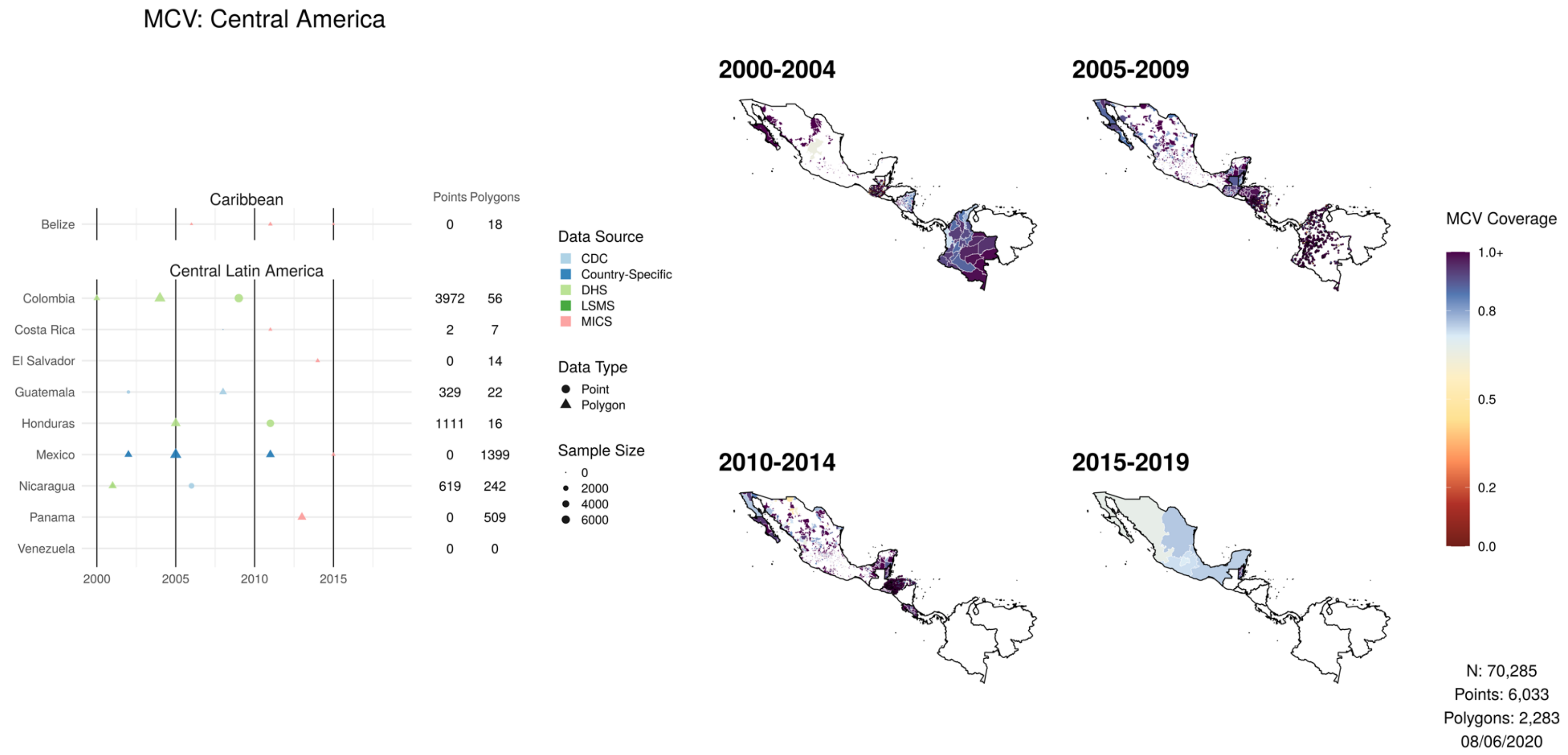


*Supplementary Fig. 1b.* Central America

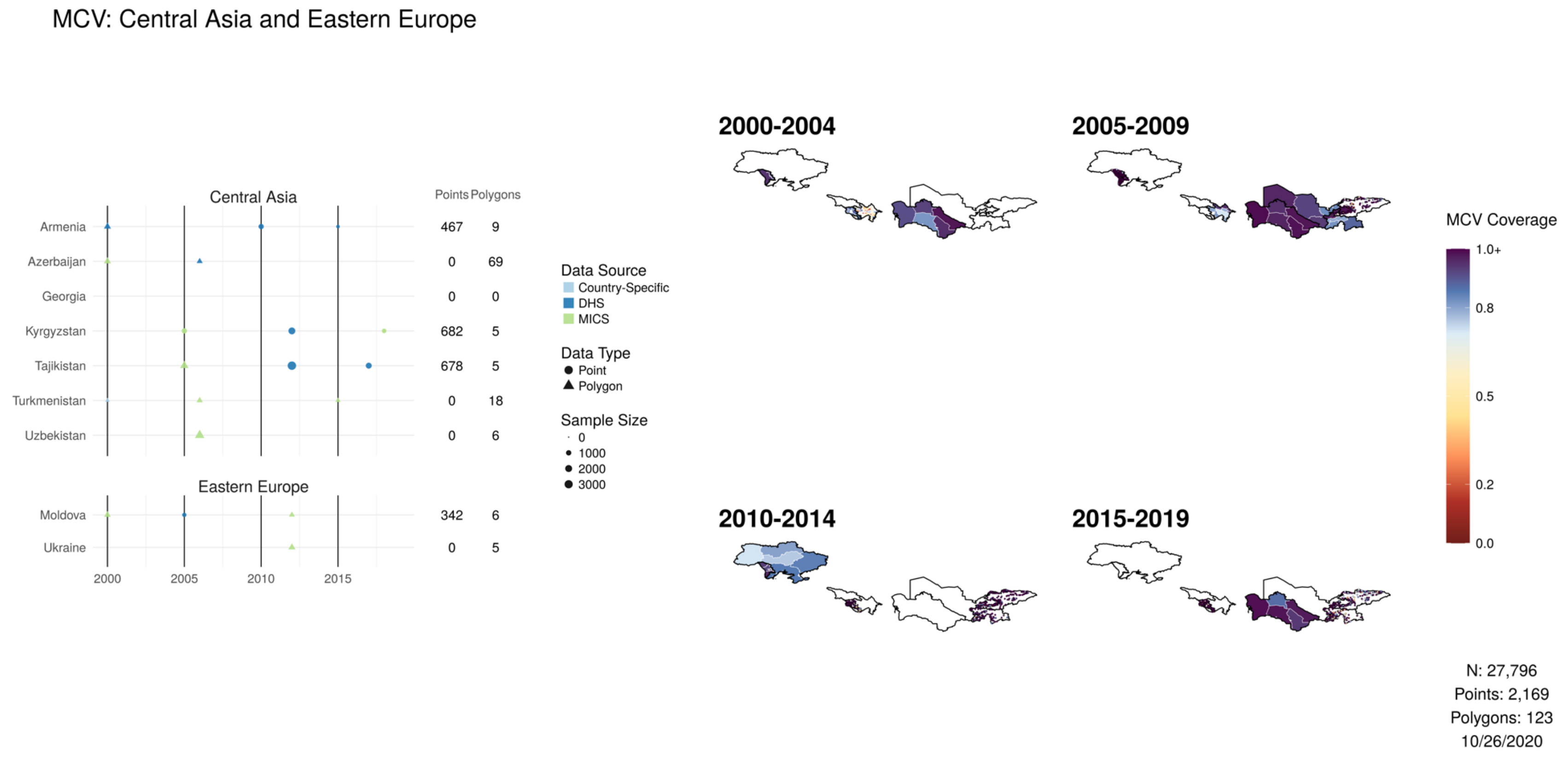


*Supplementary Fig. 1c.* Central Asia and Eastern Europe

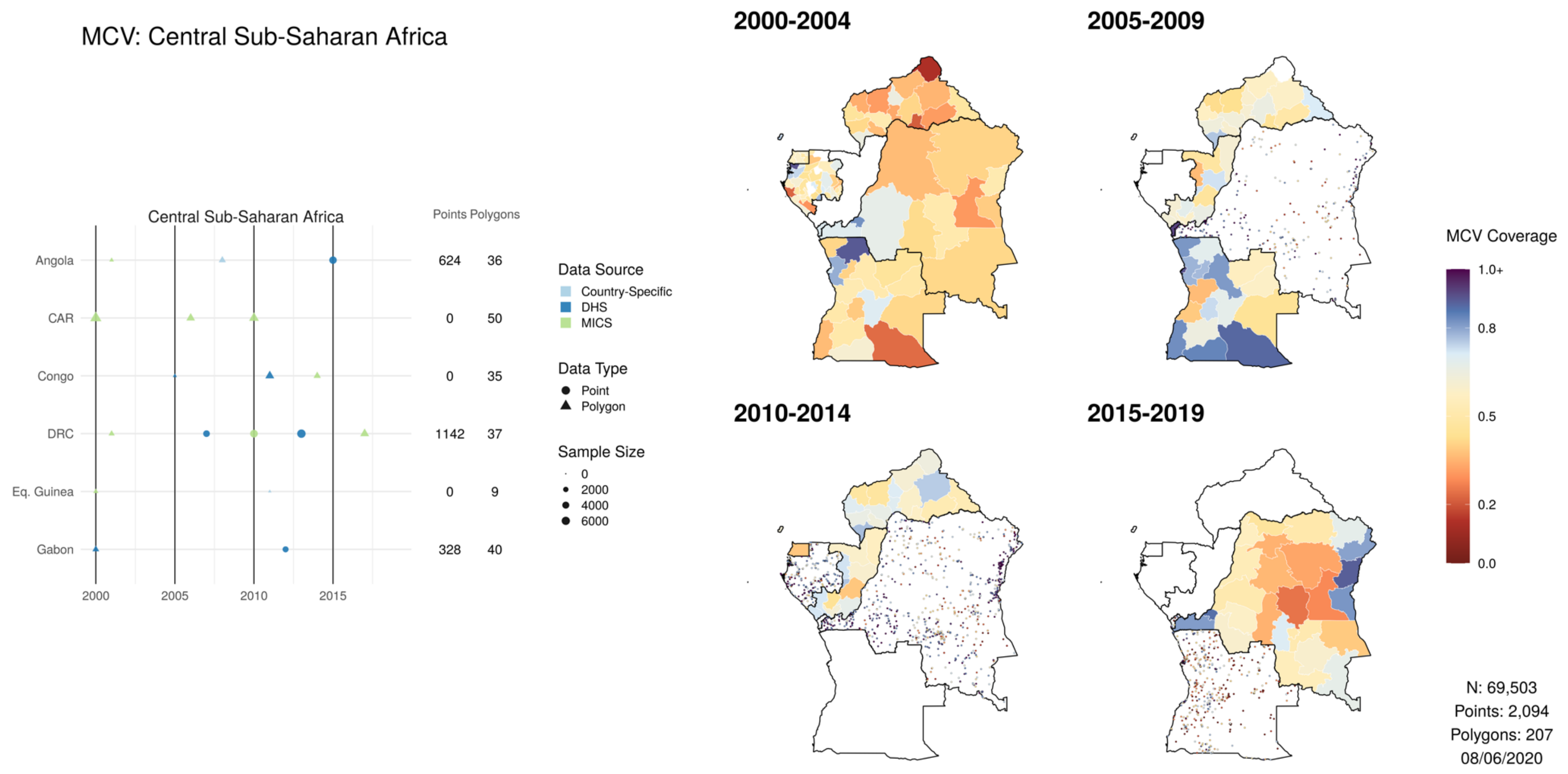


*Supplementary Fig. 1d.* Central Sub-Saharan Africa

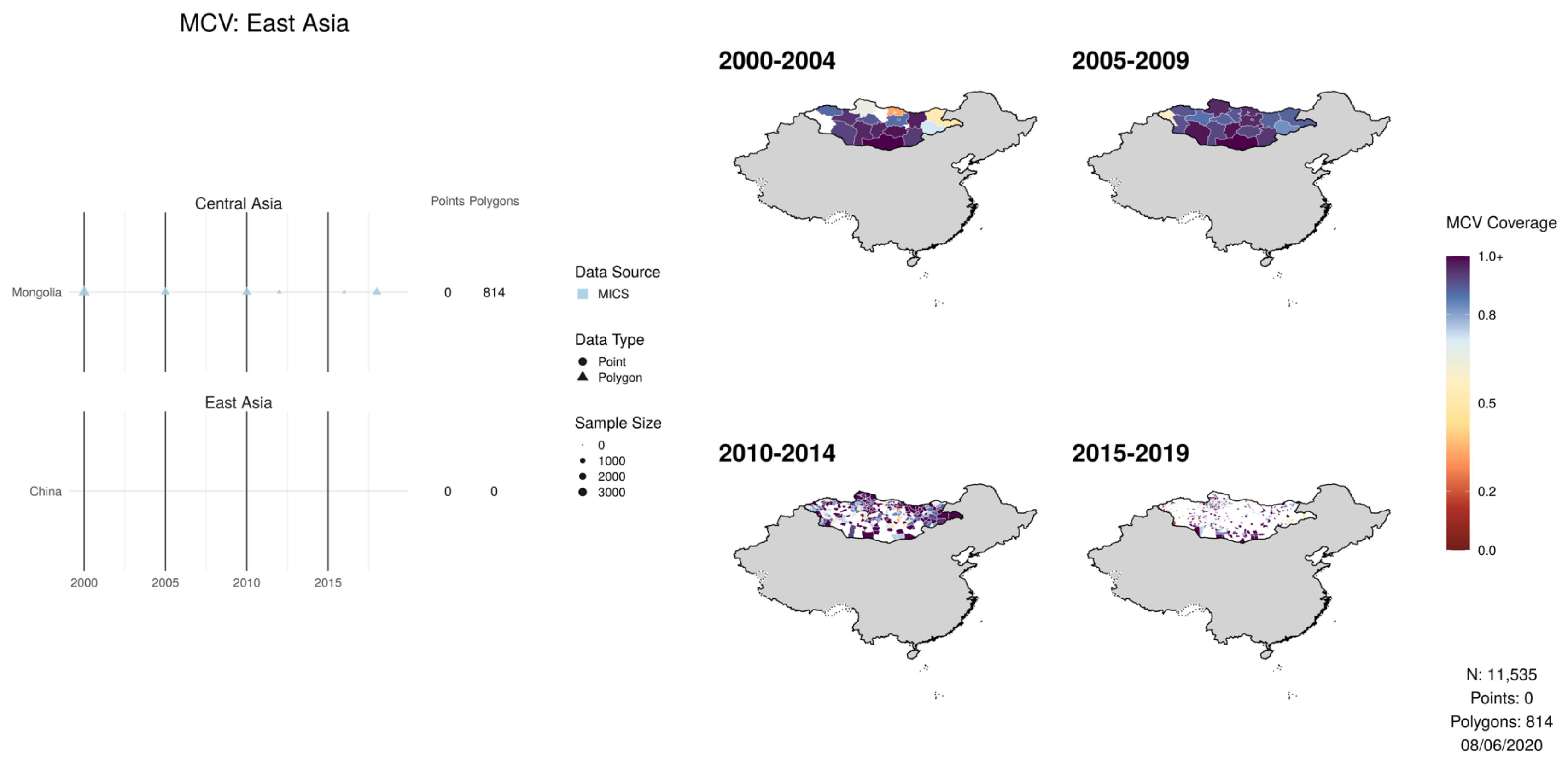


*Supplementary Fig. 1e.* East Asia

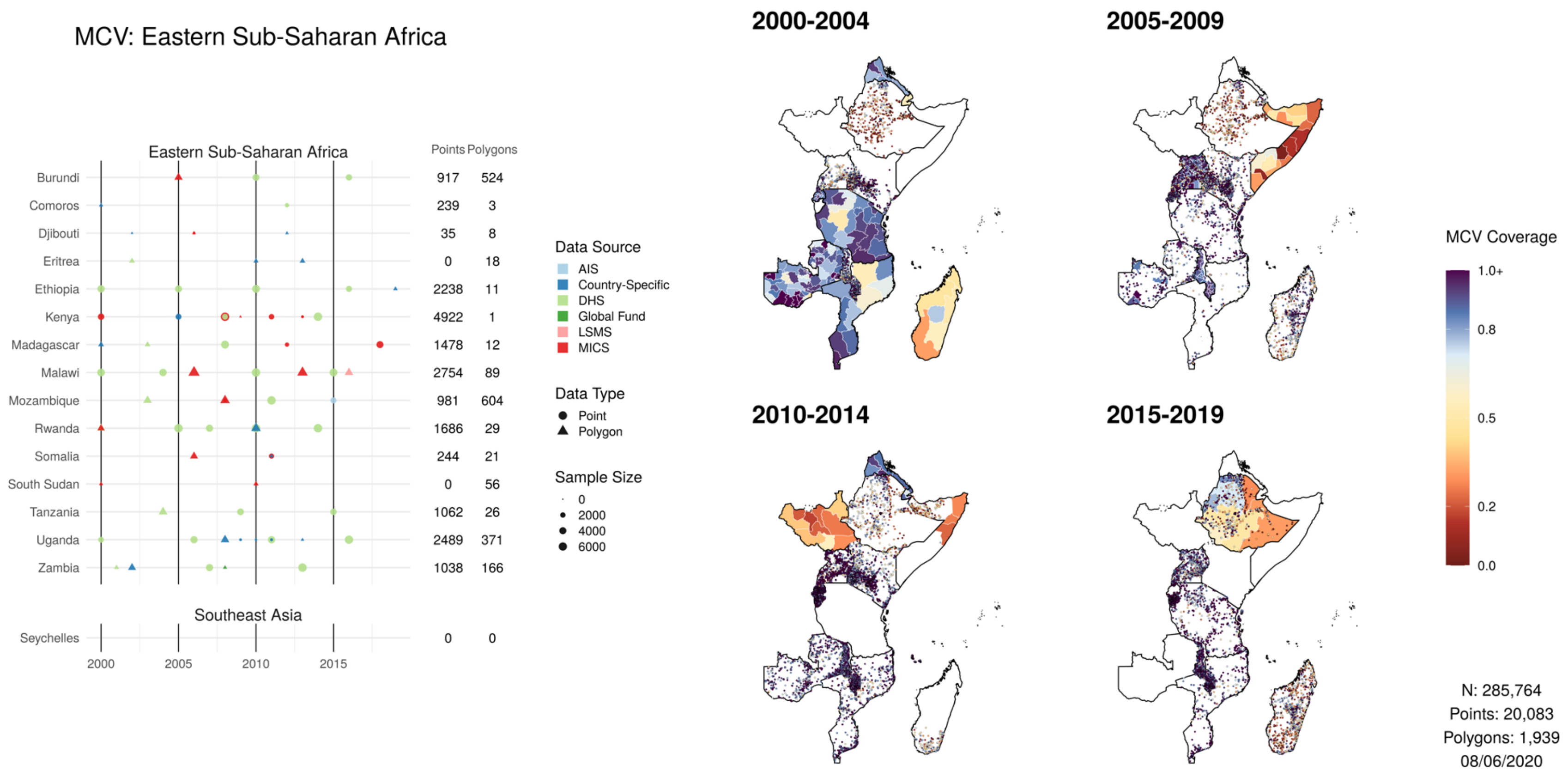


*Supplementary Fig. 1f.* Eastern Sub-Saharan Africa

MCV: North Africa and Middle East

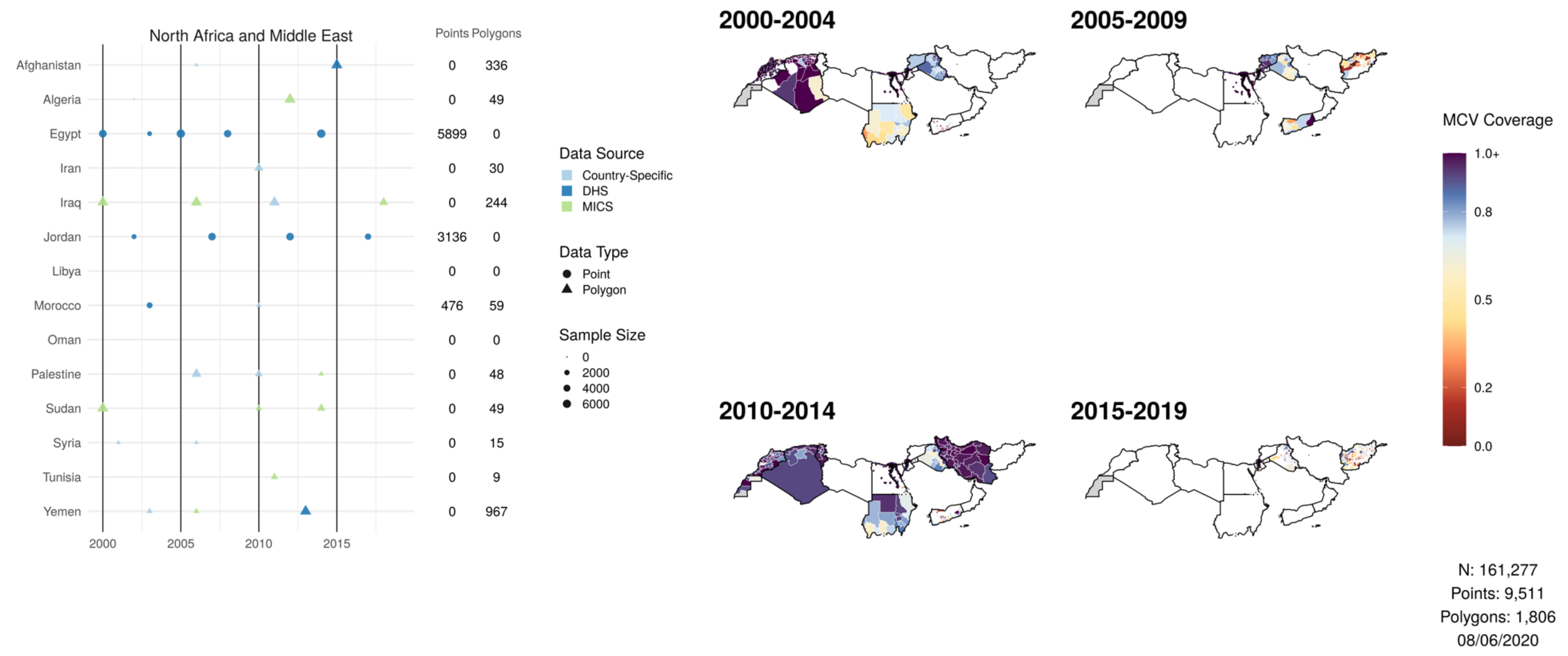


*Supplementary Fig. 1g.* North Africa and Middle East

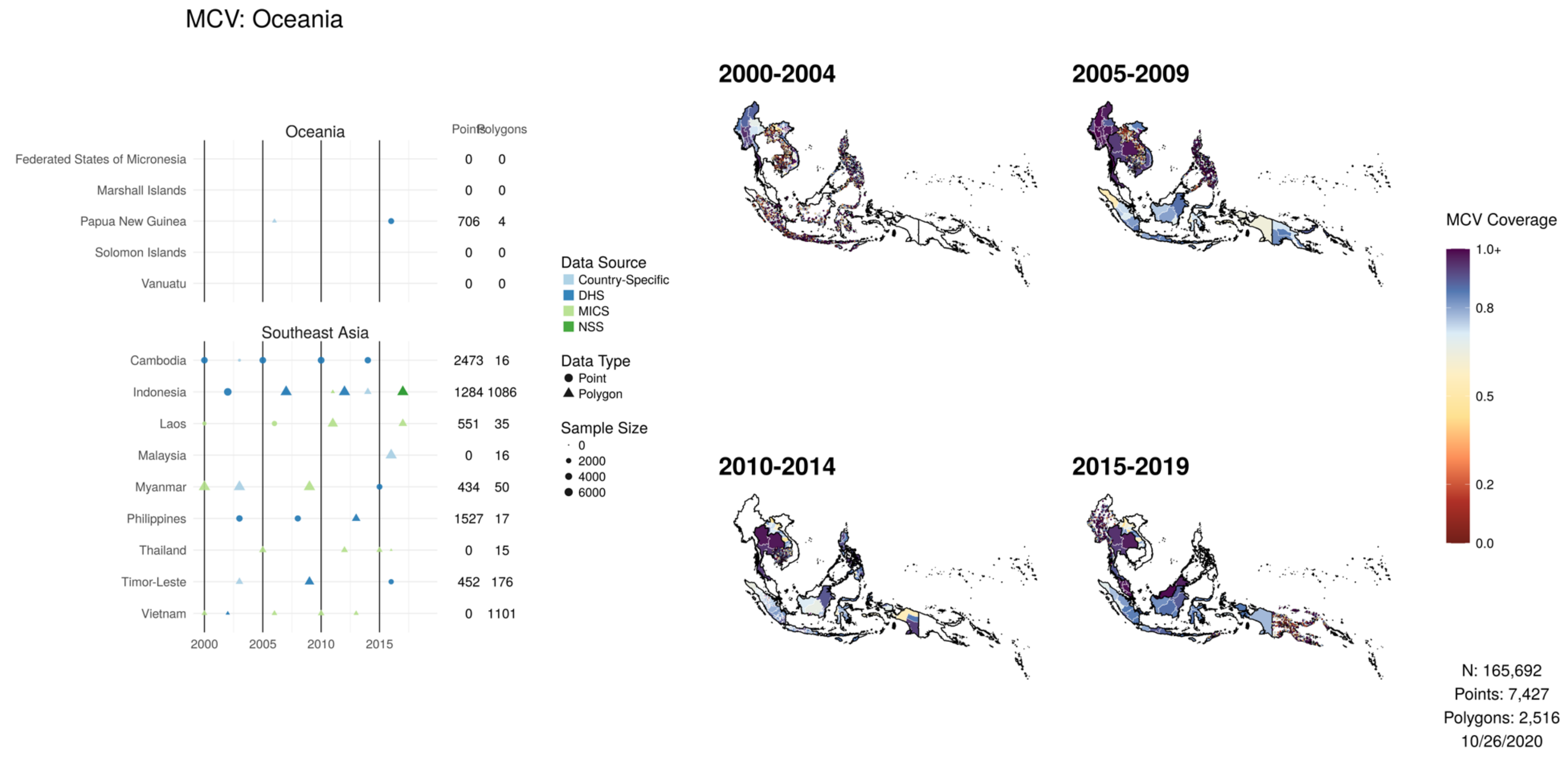


*Supplementary Fig. 1h.* Oceania

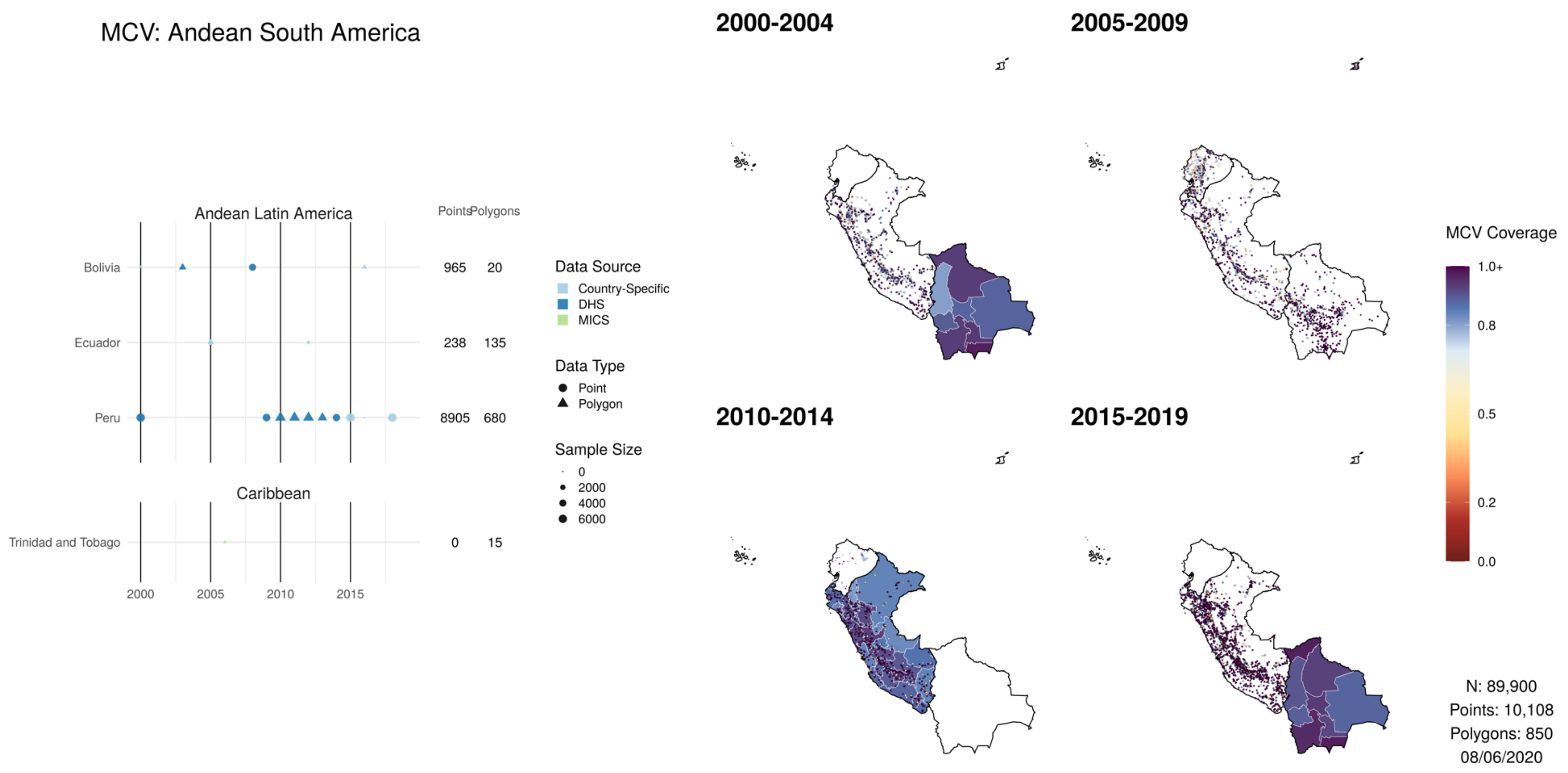


*Supplementary Fig. 1i.* Andean South America

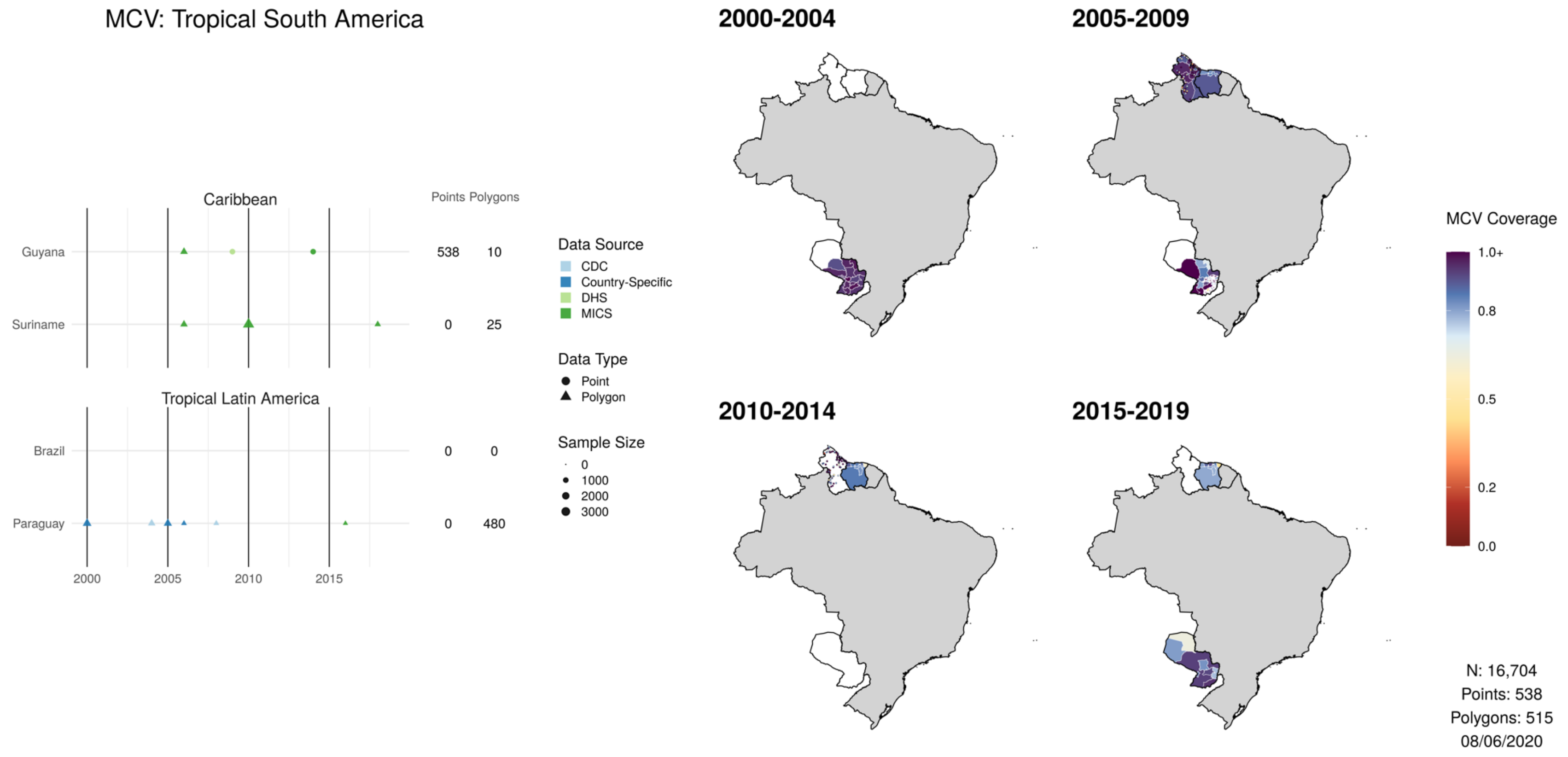


*Supplementary Fig. 1j.* Tropical South America

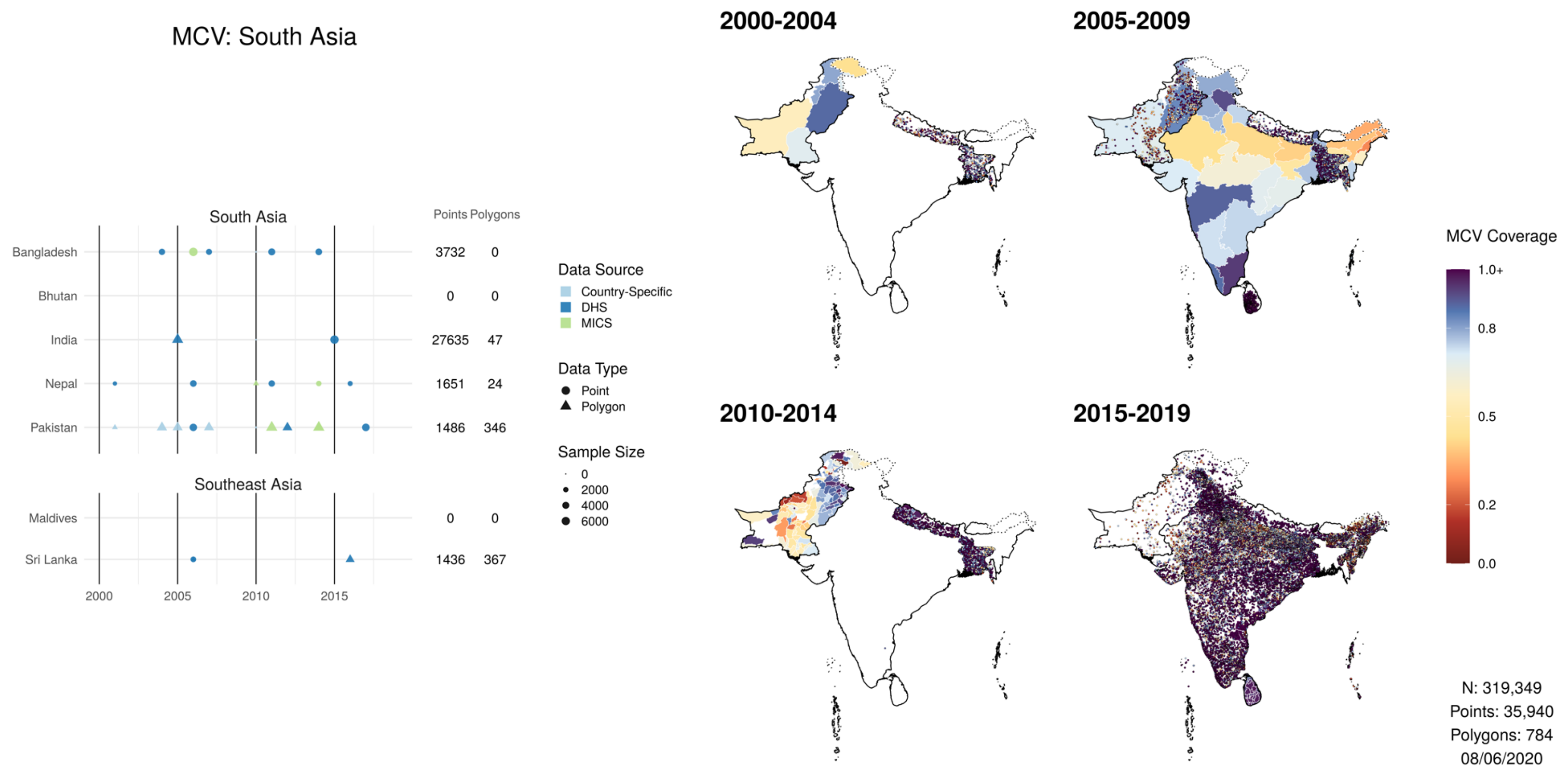


*Supplementary Fig. 1k.* South Asia

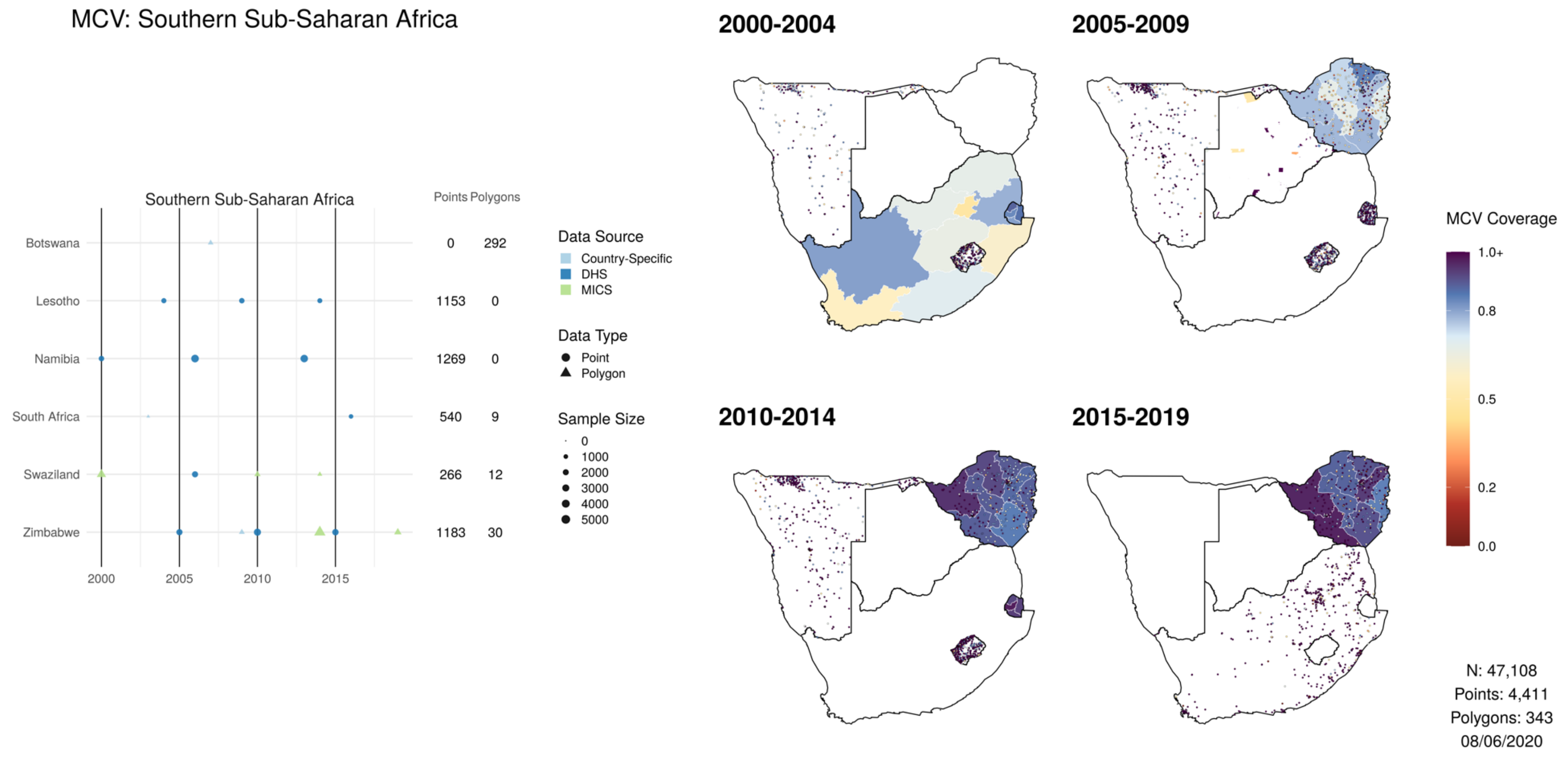


*Supplementary Fig. 11.* Southern Sub-Saharan Africa

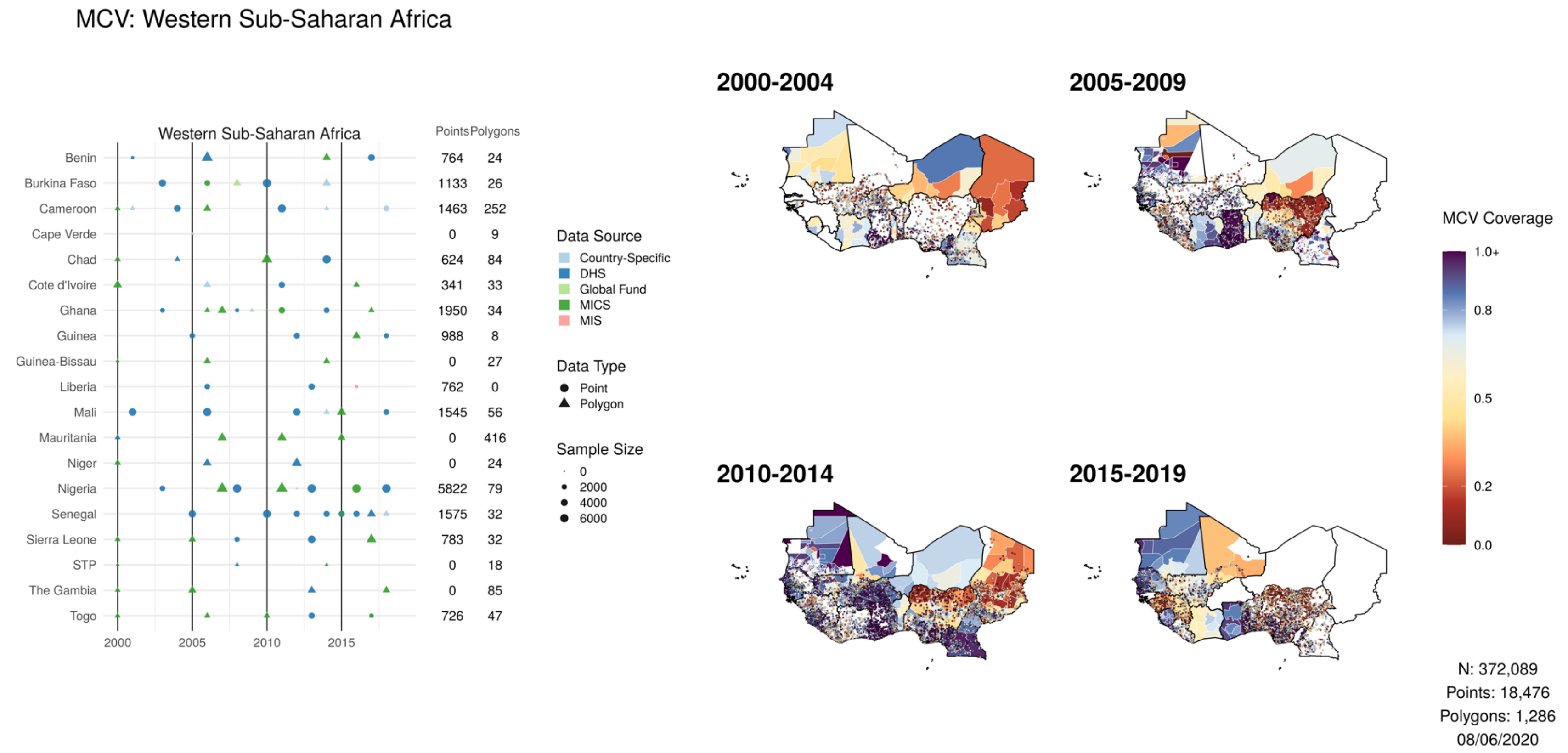


*Supplementary Fig. 1m.* Western Sub-Saharan Africa

a.

b.

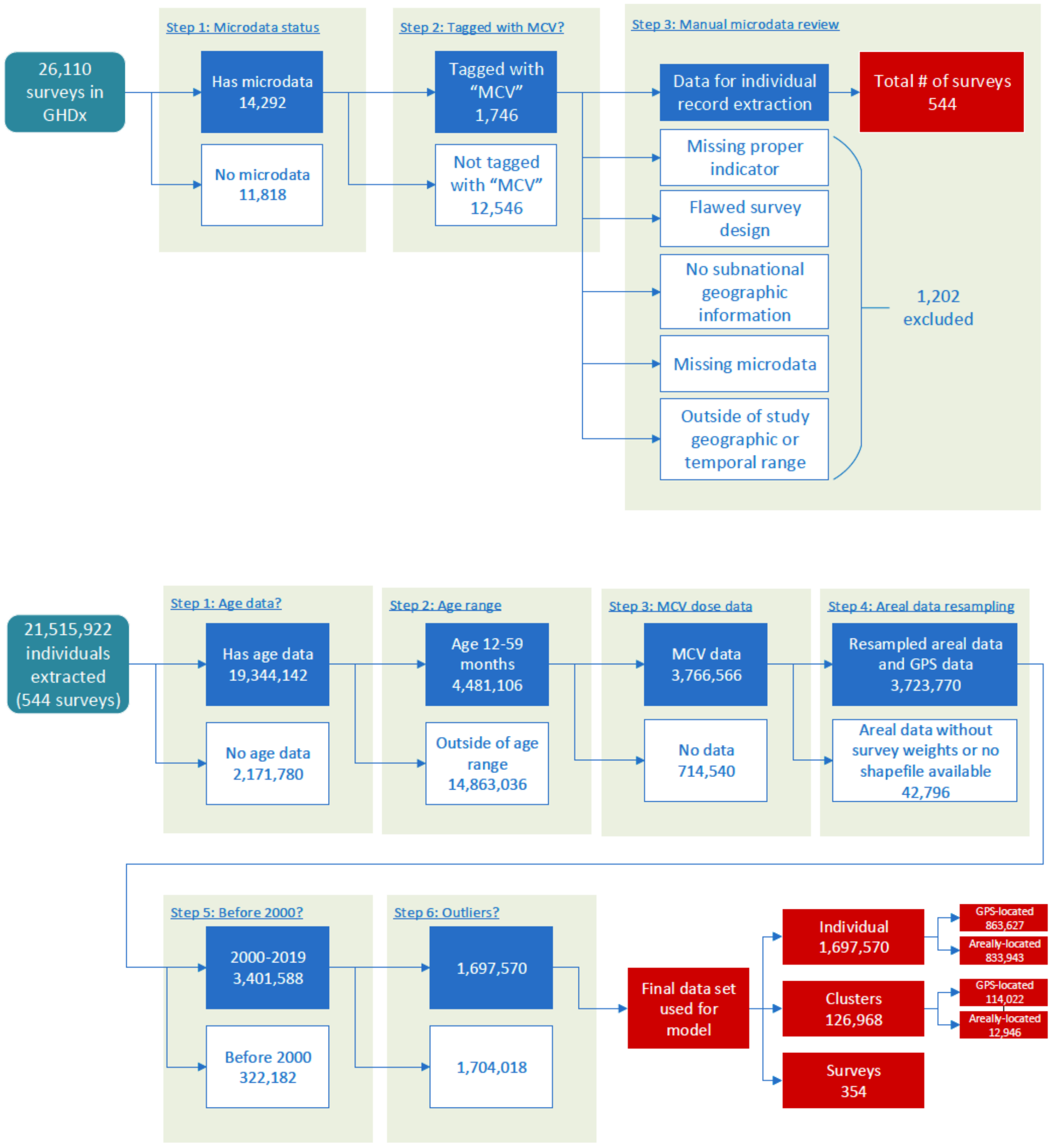


*Supplementary Fig. 2:* Data inclusion flow chart

Survey identification from the GHDx and data loss during cleaning and processing is described below.[11] To be included in final analyses, data had to be within the countries included and within temporal range of the study, meet survey design standards, and contain individual-level microdata on age, MCV1 coverage, and subnational geographic location. Panel a indicates survey-level identification criteria and panel b indicates individual-level details after survey identification.

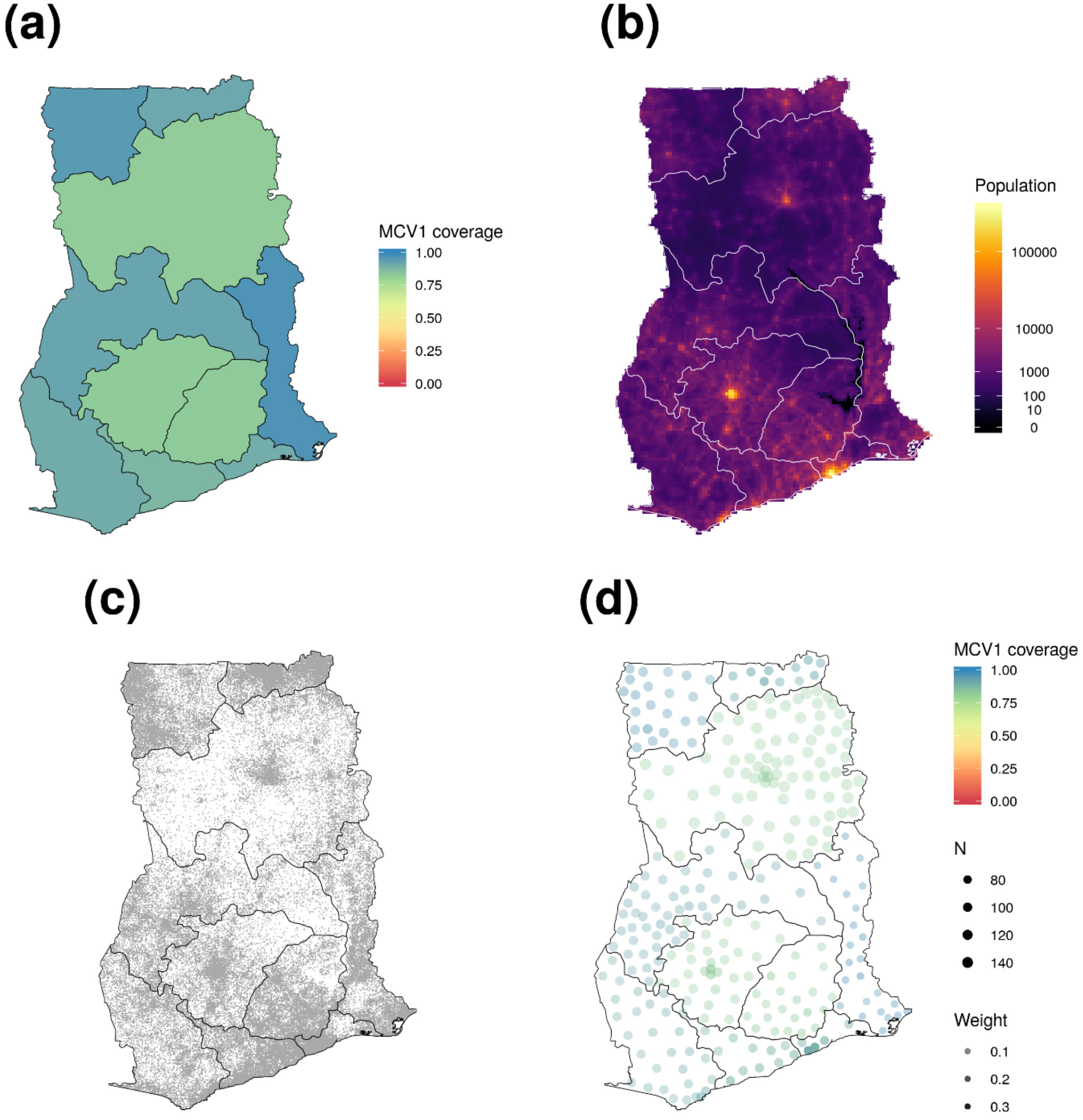


*Supplementary Fig. 3:* Areal data spatial resampling

Areal data was resampled for inclusion in the geospatial models using k-means clustering and underlying population weights, via weighted survey data estimates of MCV1 coverage for each areal unit in a given country-survey-year (panel a), population surface from WorldPop used during the resampling process (panel b), 10,000 randomly sampled points proportional to the underlying population weights of panel b (panel c), and weighted integration points following k-means clustering to be used in the geospatial model (panel d).

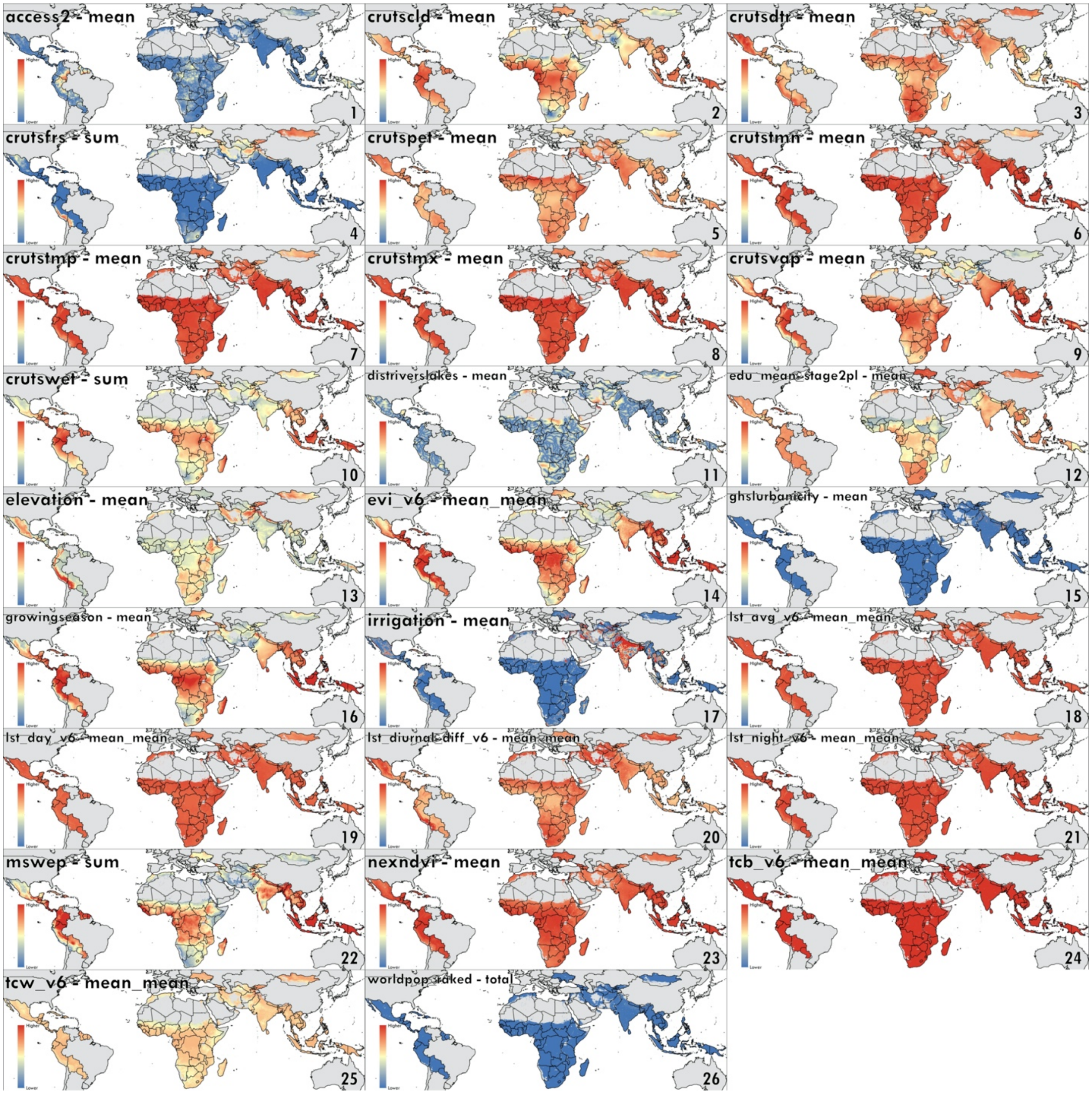


*Supplementary Fig. 4:* Geospatial covariates used in modelling
Twenty-six candidate geospatial layers of covariates possibly related to MCV1 coverage were selected for use in the stacked generalisation modelling process via a variance inflation factor selection algorithm per region. Individual covariates are cited in Supplementary Table 6. Covariates include the following, and are shown in 2019 or the most recent year available: access to roads [*access2*], cloud cover percentage [*crutscld*], mean diurnal temperature range [*crutsdtr*], frost day frequency [*crutsfrs*], mean potential evapotranspiration per day per month [crutspet], average daily minimum temperature [*crutstmn*], average daily mean temperature [*crutstmp*], average daily maximum temperature [*crutstmx*], mean vapour pressure [*crutsvap*], monthly wet day frequency [*crutswet*], distance from rivers or lakes [*distriverslakes*],

mean years of education among 15–49 year-old females (maternal education) [*edu-mean-stage2pl*], elevation [*elevation*], enhanced vegetation index [*evi_v6*], urban or rural [*ghsurbanicity*], length of growing season [*growingseason*], irrigation [*irrigation*], average land surface temperature [*lst_av_v6*], daytime land surface temperature [*lst_day_v6*], difference between daytime and nighttime land surface temperature [*lst_diurnal_v6*], nighttime land surface temperature [*lst_night_v6*], multi-source weighted-ensemble precipitation [*mswep*], normalised difference vegetation index [*nexndvi*], tasseled-cap brightness [*tcb_v6*], tasseled-cap wetness [*tcw_v6*], and total population [*worldpop_raked*]. Pixels that are grey in colour are either not included in the analysis, or have been classified by being "barren or sparsely vegetated" or had fewer than 10 people per 1 × 1-km pixel.[15,26]

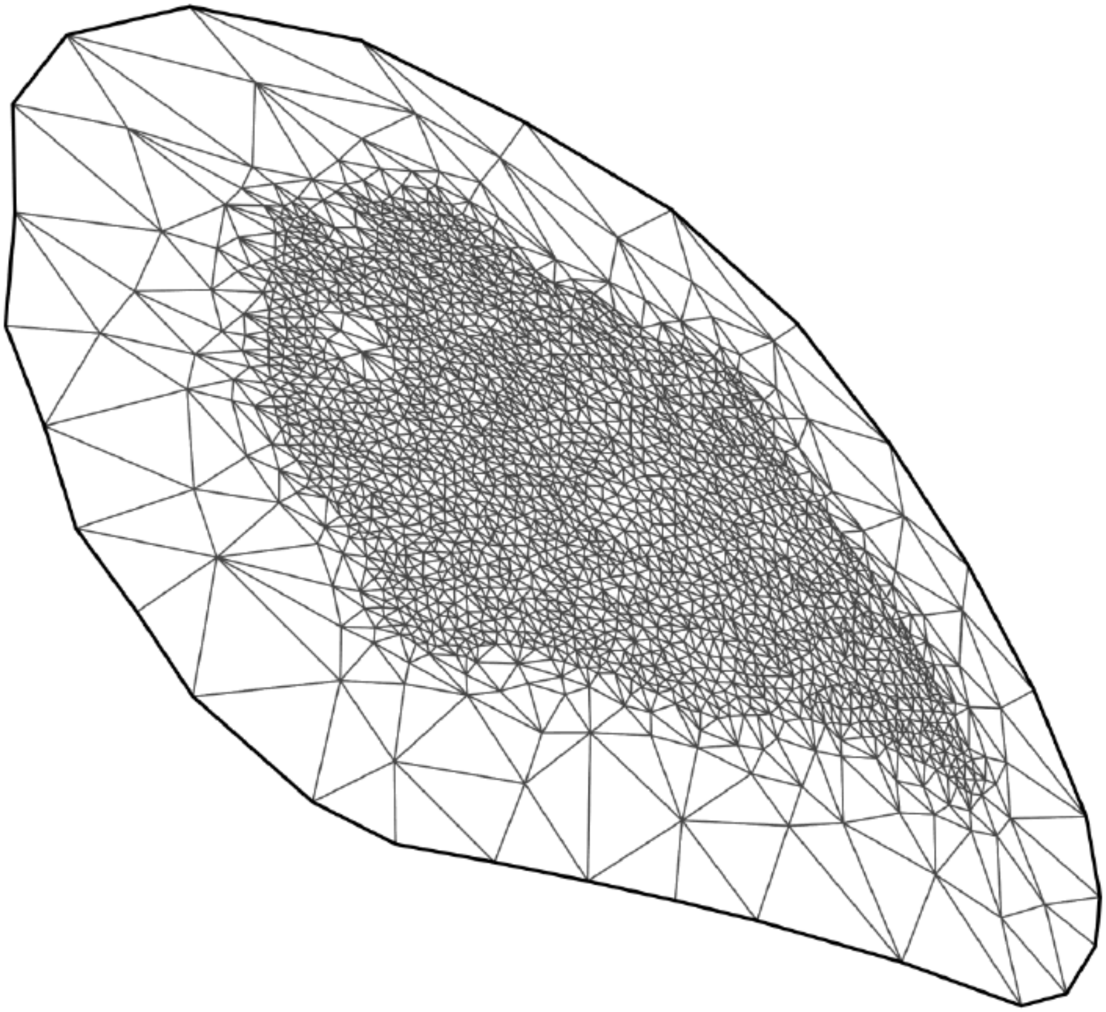

*Supplementary Fig. 5:* Example of finite spatial mesh

For the Southern sub-Saharan Africa region, the fine-scale mesh over land in the modelling region and the coarser buffer region are shown.

*Supplementary Figs 6–7:* First administrative level in-sample validation plots
For each plot, the survey data and mean posterior prediction of coverage are compared. The size per dot is proportional to the sample size in the underlying data. Estimates are shown by region and year. Since there were only data through 2018, the years included only span through 2018. Regions are labelled as the following: Andean South America [*vax_ansa*], Central Asia Eastern Europe [*vax_caeu*], Caribbean [*vax_crbn]*, Central sub-Saharan Africa [*vax_cssa*], Central America [*vax_ctam*], East Asia [*vax_eaas*], East sub-Saharan Africa [*vax_essa*], North Africa and Middle East [*vax_name*], South East Asia, [*vax_seas*], South Asia [*vax_soas*], Southern sub-Saharan Africa [*vax_sssa*], Tropical South America [*vax_trsa*], and Western sub-Saharan Africa [*vax_wssa*].

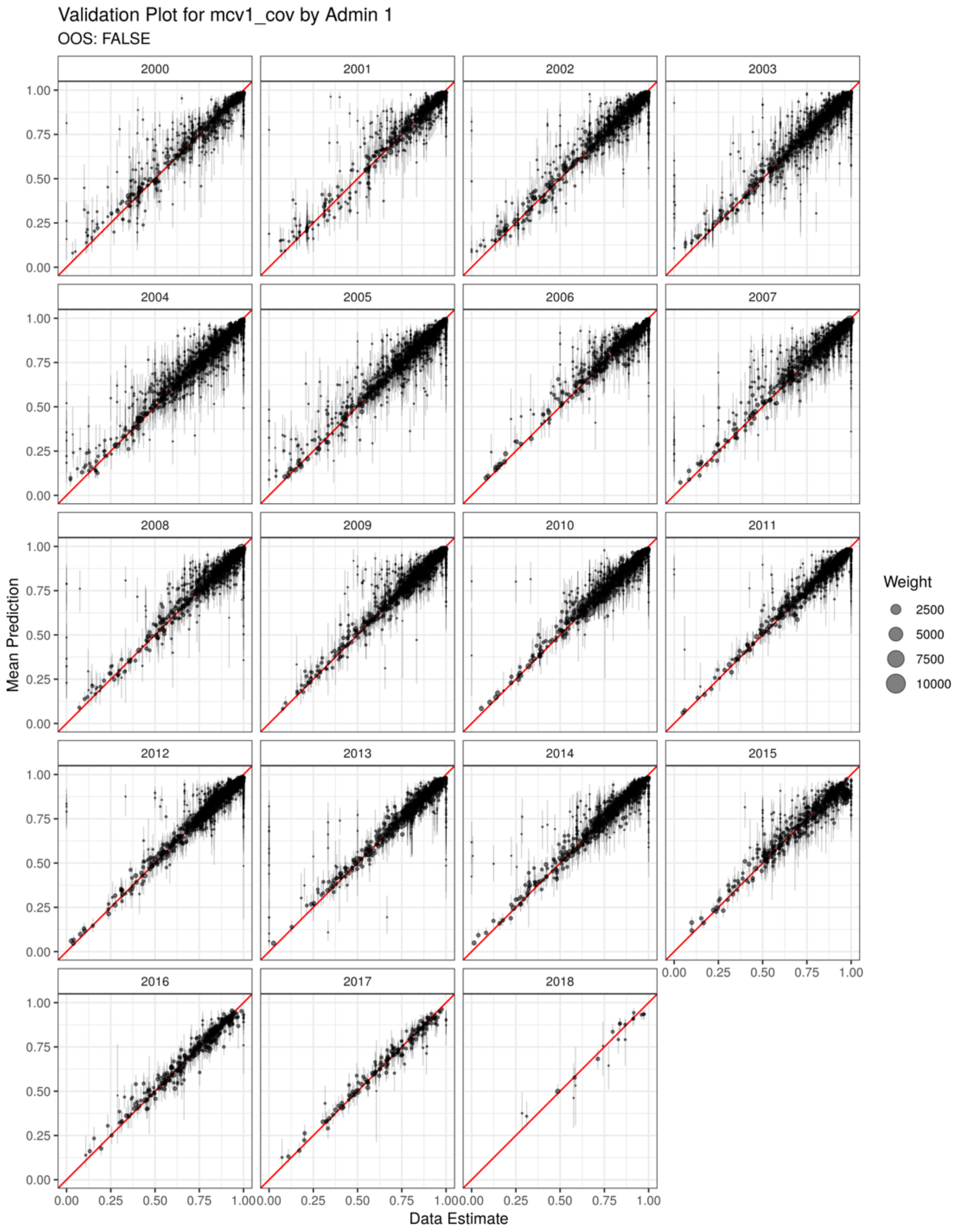


*Supplementary Fig. 6:* First administrative level, in-sample validation plot, by year

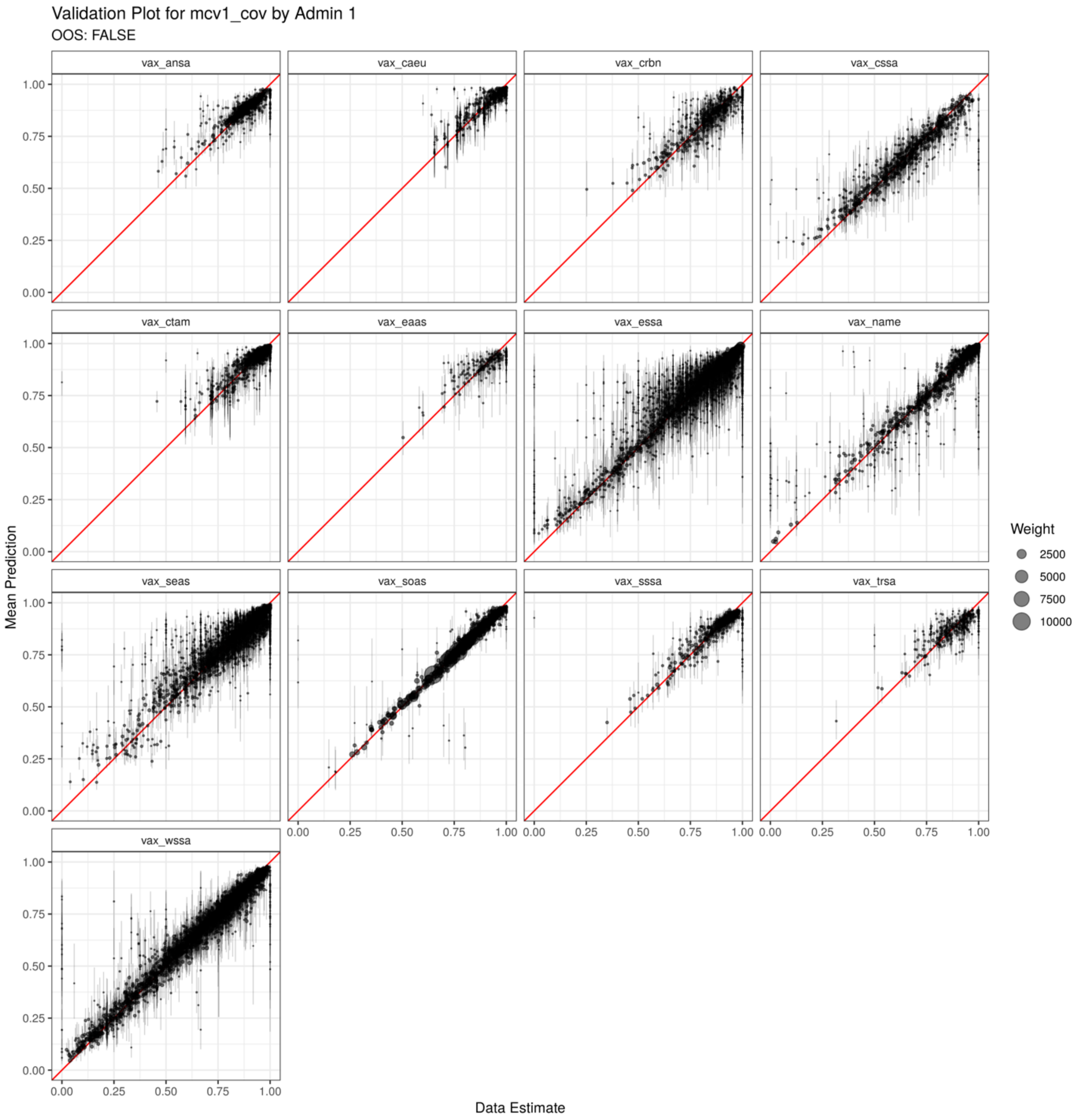


*Supplementary Fig. 7:* First administrative level, in-sample validation plot, by region

*Supplementary Figs 8–9:* Second administrative level in-sample validation plots
For each plot, the survey data and mean posterior prediction of coverage are compared. The size per dot is proportional to the sample size in the underlying data. Estimates are shown by region and year. Since there were only data through 2018, the years included only span through 2018. Regions are labelled as the following: Andean South America [*vax_ansa*], Central Asia Eastern Europe [*vax_caeu*], Caribbean [*vax_crbn]*, Central sub-Saharan Africa [*vax_cssa*], Central America [*vax_ctam*], East Asia [*vax_eaas*], East sub-Saharan Africa [*vax_essa*], North Africa and Middle East [*vax_name*], South East Asia, [*vax_seas*], South Asia [*vax_soas*], Southern sub-Saharan Africa [*vax_sssa*], Tropical South America [*vax_trsa*], and Western sub-Saharan Africa [*vax_wssa*].

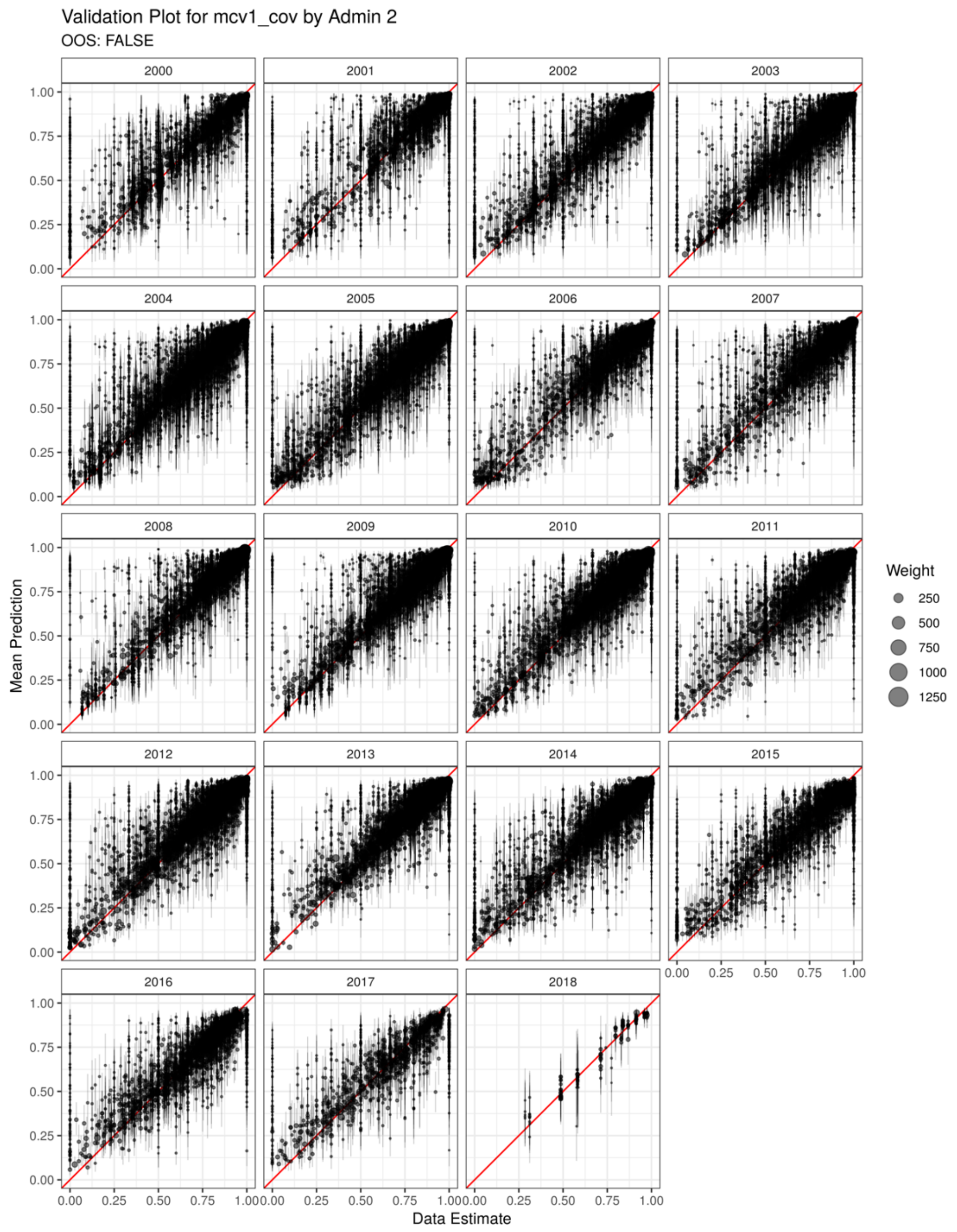


*Supplementary Fig. 8:* Second administrative level, in-sample validation plot, by year

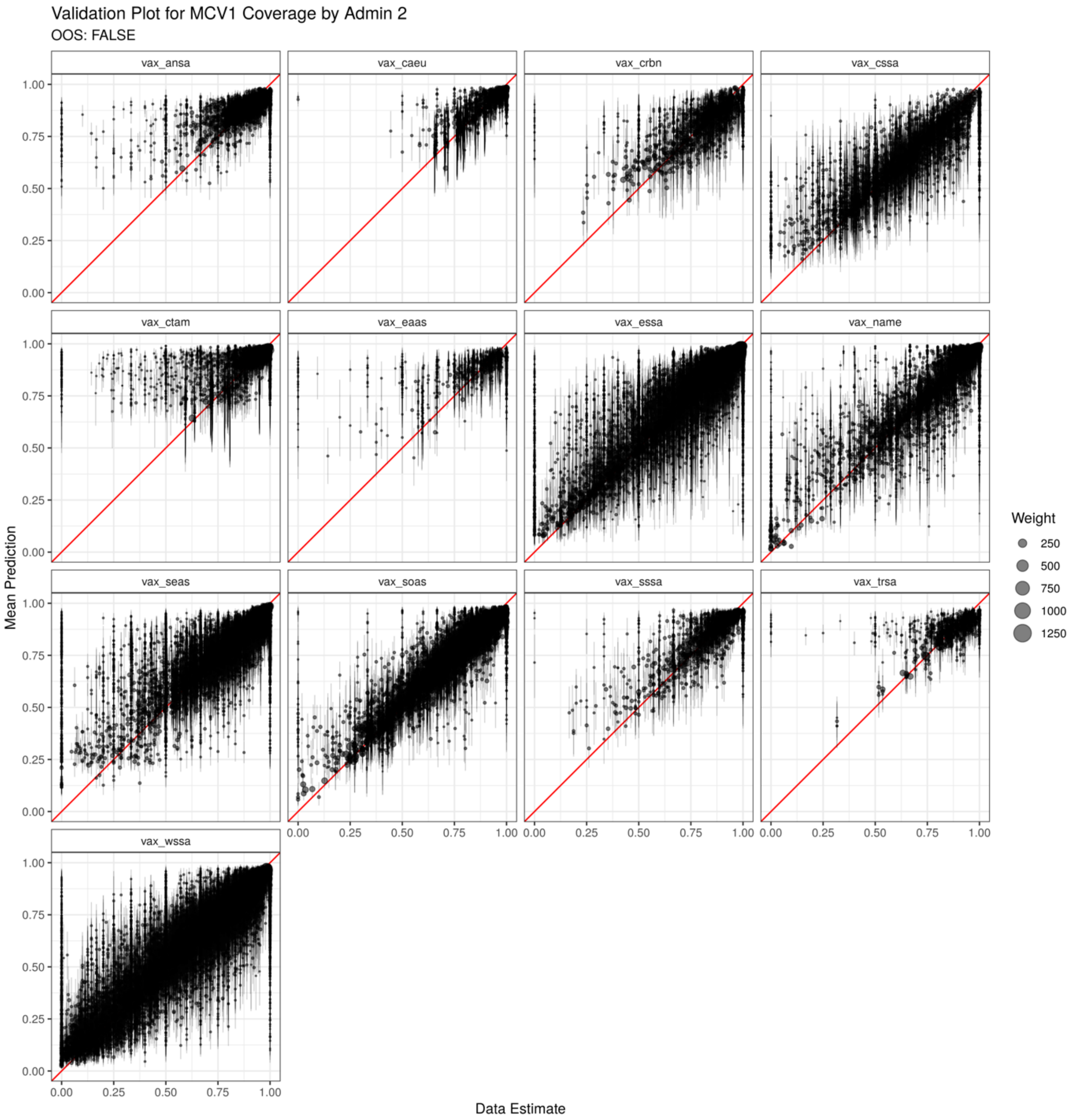


*Supplementary Fig. 9:* Second administrative level, in-sample validation plot, by region

*Supplementary Figs 10–11:* First administrative level out-of-sample validation plots
For each plot, the survey data and mean posterior prediction of coverage are compared. The size per dot is proportional to the sample size in the underlying data. Estimates are shown by region and year. Since there were only data through 2018, the years included only span through 2018. Regions are labelled as the following: Andean South America [*vax_ansa*], Central Asia Eastern Europe [*vax_caeu*], Caribbean [*vax_crbn]*, Central sub-Saharan Africa [*vax_cssa*], Central America [*vax_ctam*], East Asia [*vax_eaas*], East sub-Saharan Africa [*vax_essa*], North Africa and Middle East [*vax_name*], South East Asia, [*vax_seas*], South Asia [*vax_soas*], Southern sub-Saharan Africa [*vax_sssa*], Tropical South America [*vax_trsa*], and Western sub-Saharan Africa [*vax_wssa*].

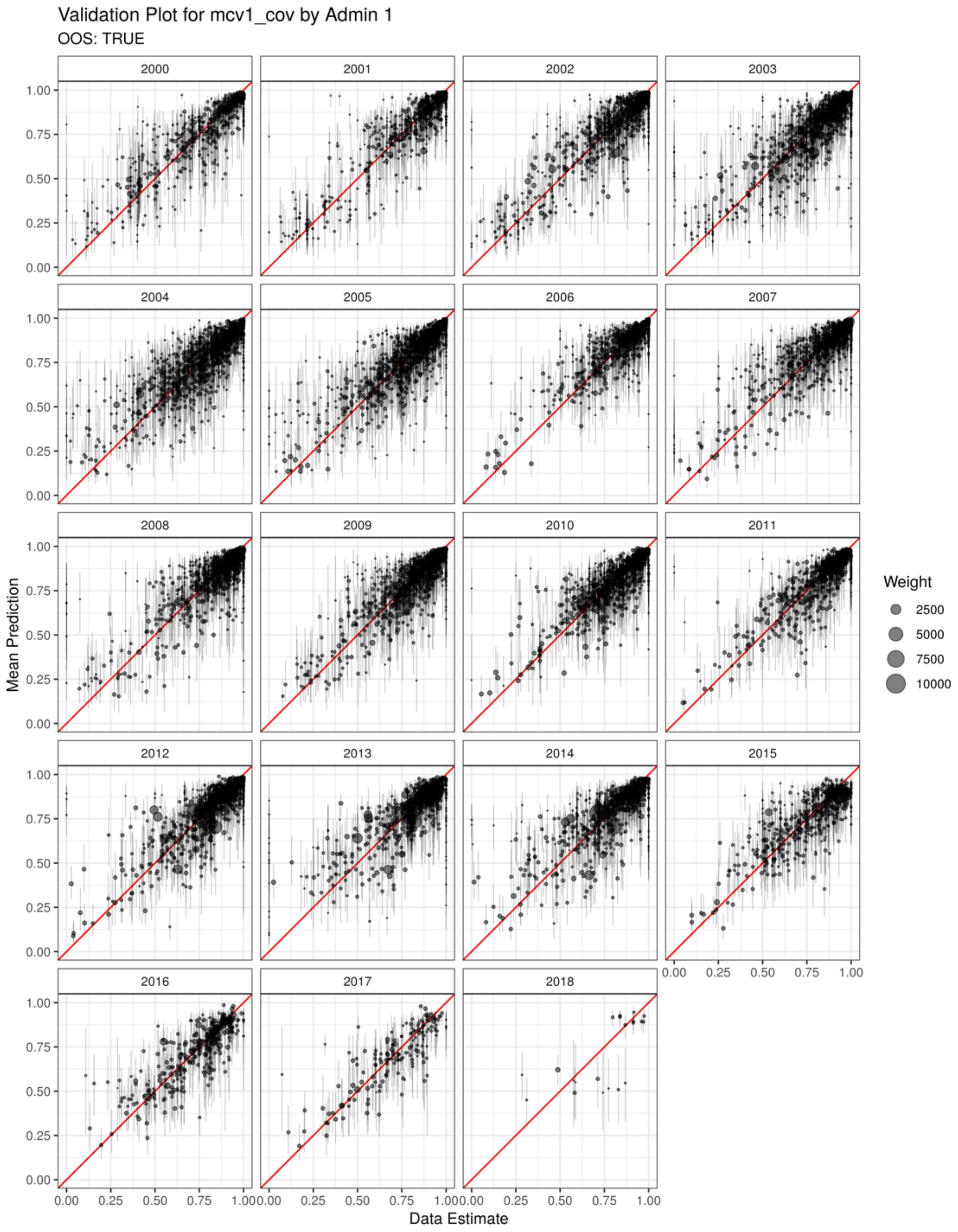


*Supplementary Fig. 10:* First administrative level, out-of-sample validation plot, by year

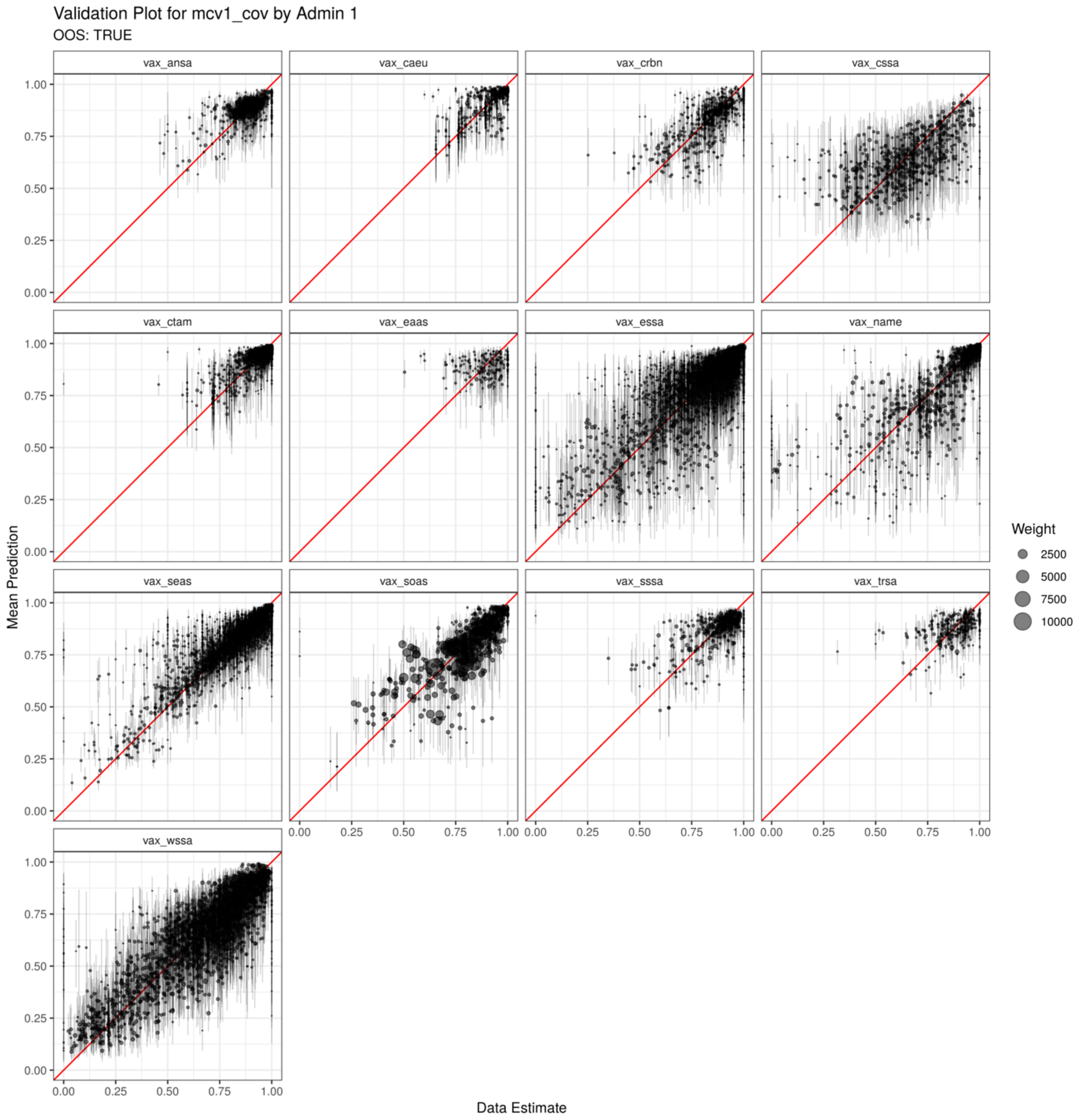


*Supplementary Fig. 11:* First administrative level, out-of-sample validation plot, by region

*Supplementary Figs 12–13:* Second administrative level out-of-sample validation plots

For each plot, the survey data and mean posterior prediction of coverage are compared. The size per dot is proportional to the sample size in the underlying data. Estimates are shown by region and year. Since there were only data through 2018, the years included only span through 2018. Regions are labelled as the following: Andean South America [*vax_ansa*], Central Asia Eastern Europe [*vax_caeu*], Caribbean [*vax_crbn]*, Central sub-Saharan Africa [*vax_cssa*], Central America [*vax_ctam*], East Asia [*vax_eaas*], East sub-Saharan Africa [*vax_essa*], North Africa and Middle East [*vax_name*], South East Asia, [*vax_seas*], South Asia [*vax_soas*], Southern sub-Saharan Africa [*vax_sssa*], Tropical South America [*vax_trsa*], and Western sub-Saharan Africa [*vax_wssa*].

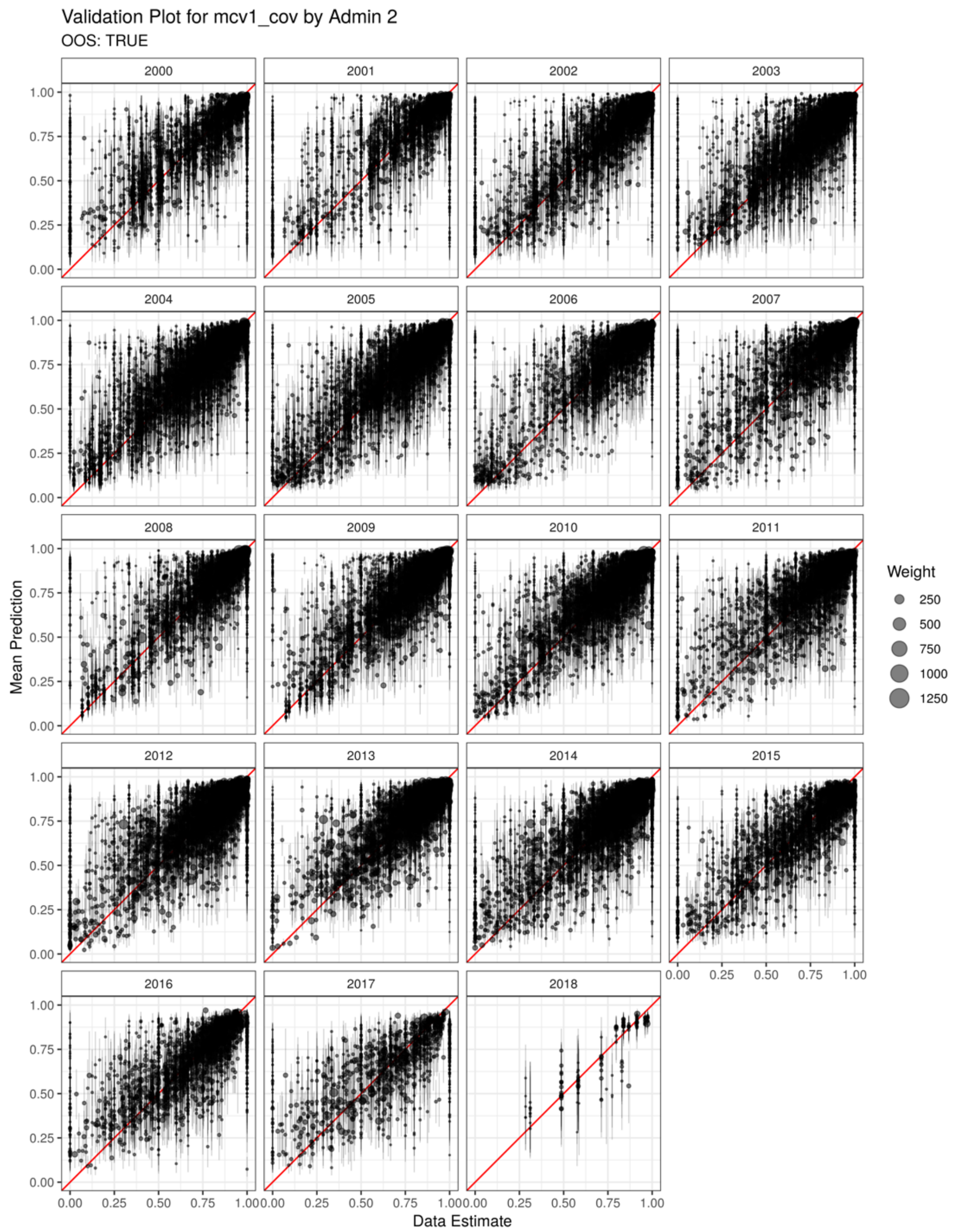


*Supplementary Fig. 12:* Second administrative level, out-of-sample validation plot, by year

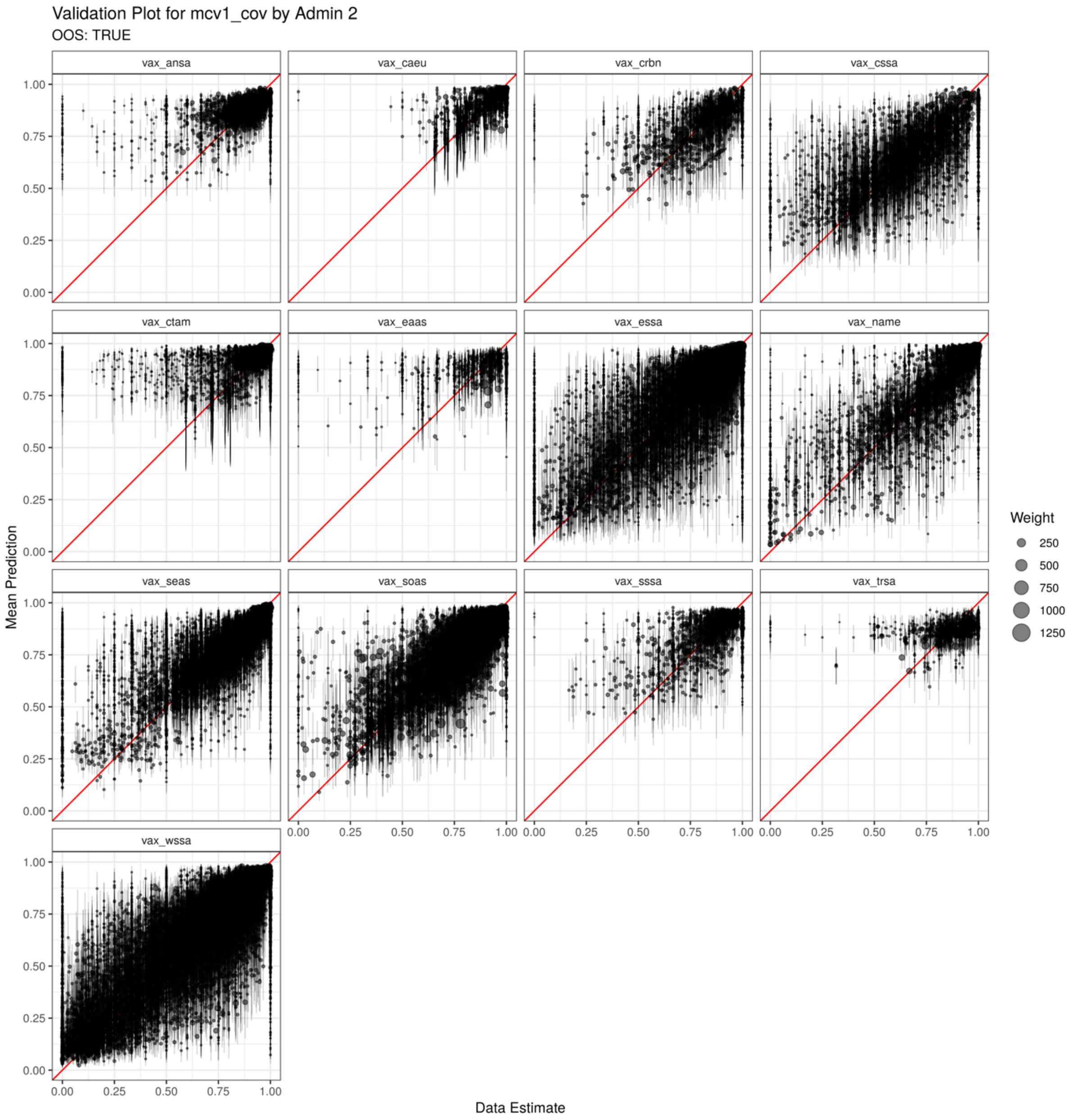


*Supplementary Fig. 13:* Second administrative level, out-of-sample validation plot, by region

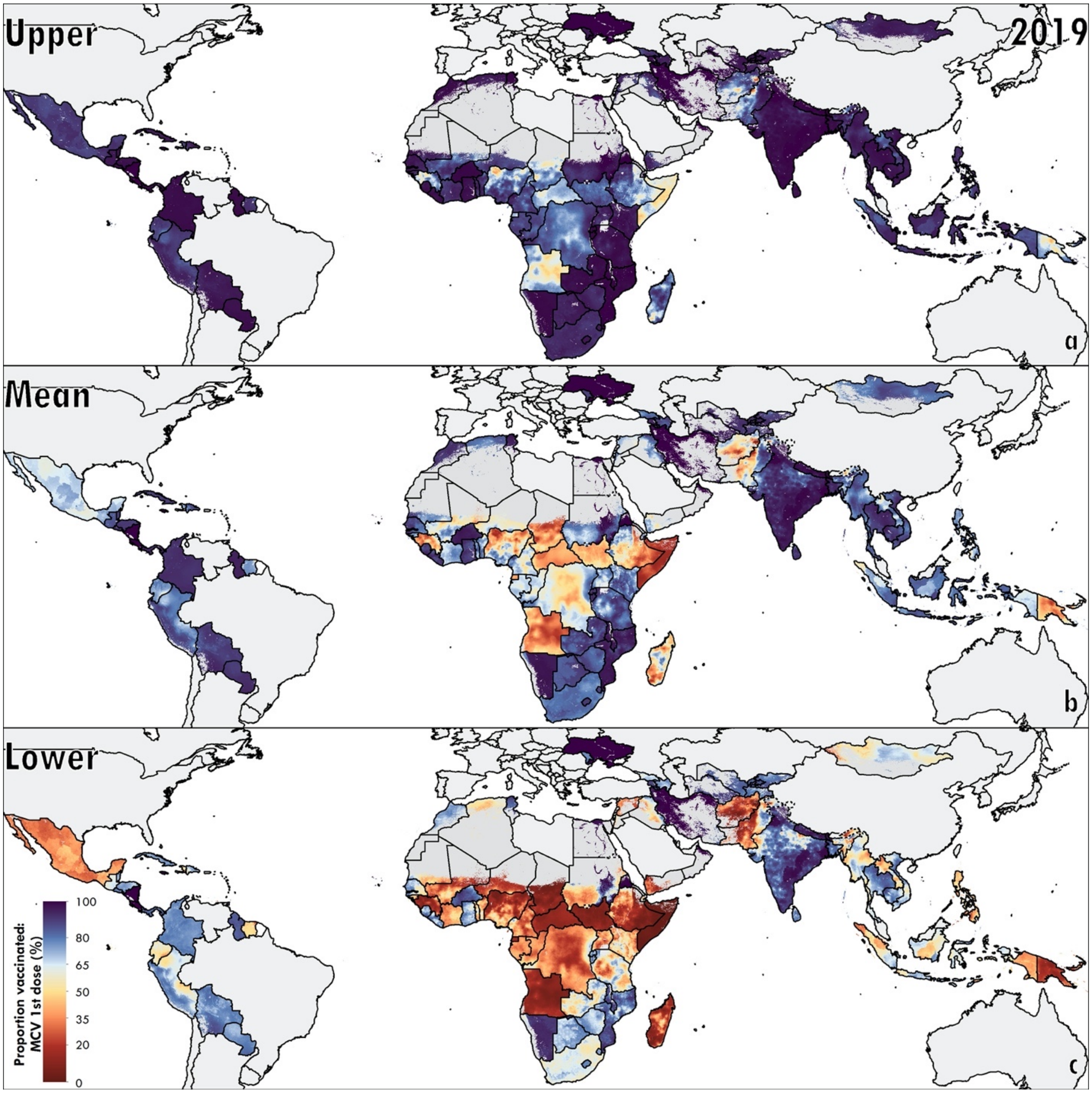


*Supplementary Fig. 14:* Pixel-level mean, lower, and upper uncertainty limits of MCV1 coverage, 2019 Posterior means and 95% uncertainty intervals are shown at the 5 × 5-km pixel level. Pixels that are grey in colour are either not included in the analysis, or have been classified by being “barren or sparsely vegetated” or had fewer than 10 people per 1 × 1-km pixel[15,26].

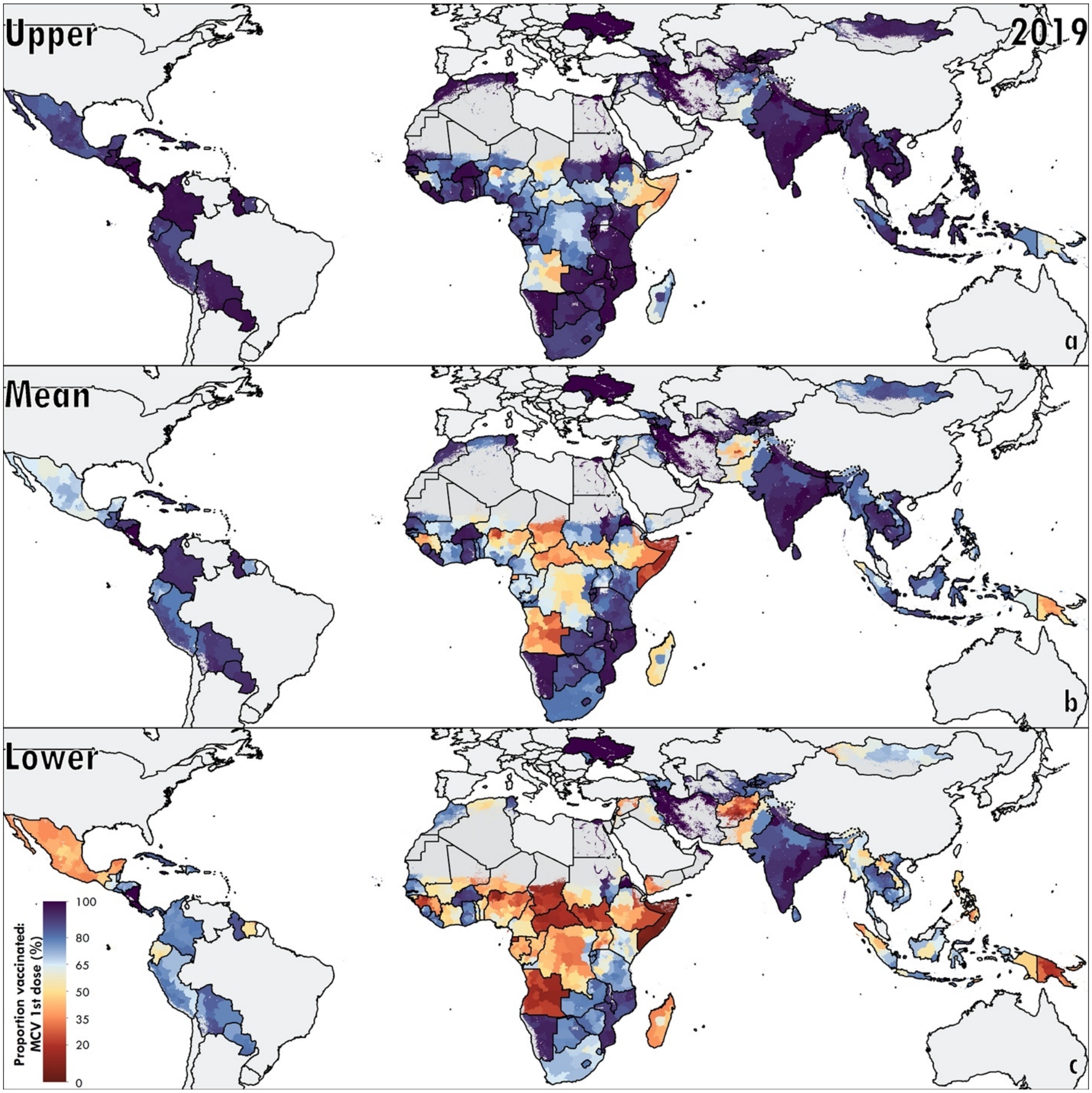


*Supplementary Fig. 15:* First administrative level mean, lower, and upper uncertainty limits of MCV1 coverage, 2019

Posterior means and 95% uncertainty intervals are shown at the first administrative level. Pixels that are grey in colour are either not included in the analysis, or have been classified by being "barren or sparsely vegetated" or had fewer than 10 people per 1 × 1-km pixel[15,26].

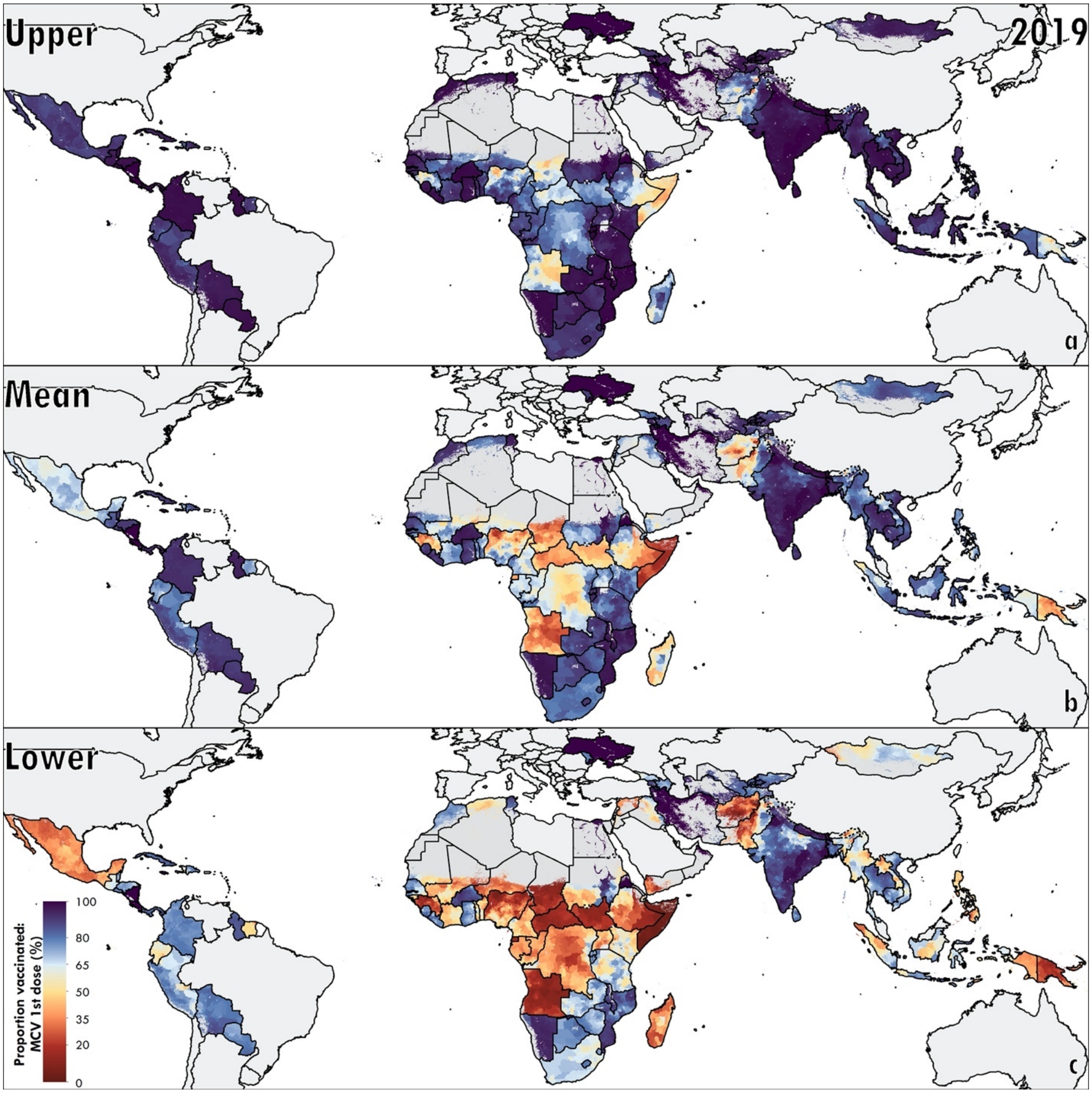


*Supplementary Fig. 16:* Second administrative level mean, lower, and upper uncertainty limits of MCV1 coverage, 2019

Posterior means and 95% uncertainty intervals are shown at the second administrative level. Pixels that are grey in colour are either not included in the analysis, or have been classified by being "barren or sparsely vegetated" or had fewer than 10 people per 1 × 1-km pixel[15,26].

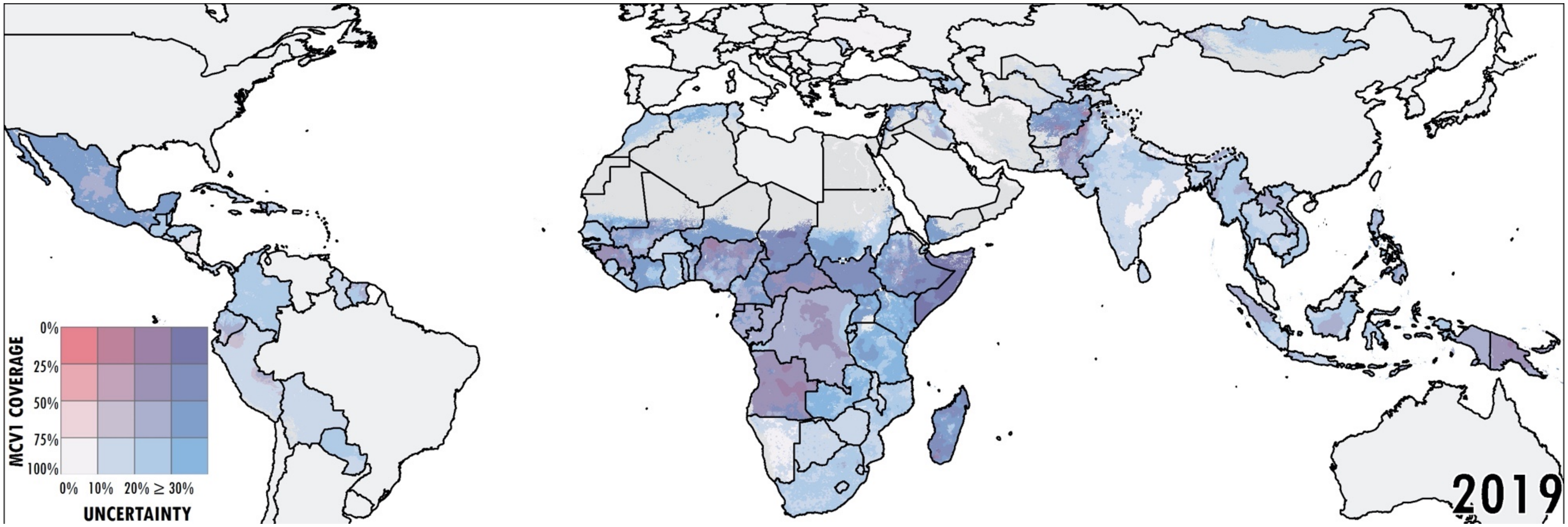


*Supplementary Fig. 17:* District level MCV1 coverage as function of uncertainty via Coffey-Feingold-Bromberg metric, 2019
Intensity of pink indicates areas that have lower uncertainty and lower coverage; intensity of blue indicates areas with higher coverage but also higher uncertainty. Purple indicates areas that have both low coverage and also higher uncertainty. Pixels that are grey in colour are either not included in the analysis, or have been classified by being "barren or sparsely vegetated" or had fewer than 10 people per 1 × 1-km pixel[15,26].

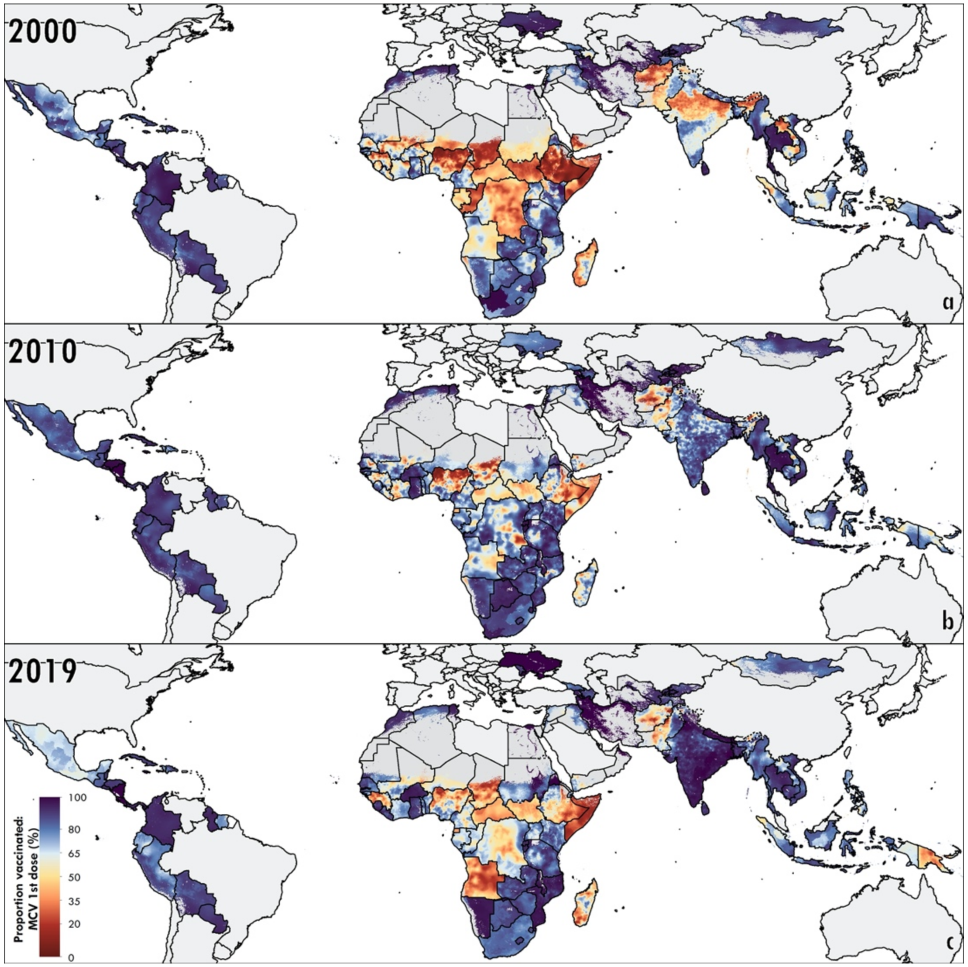


*Supplementary Fig. 18:* Estimated first-dose measles-containing vaccine (MCV1) coverage among districts in 101 low- and middle-income countries, 2000–2019, full age cohort model.

MCV1 coverage among target population in only the target age cohort in districts in 2000 (a), 2010 (b), and 2019 (c). Countries excluded from the analysis and pixels classified as ‘barren or sparsely vegetated’ based on European Space Agency Climate Change Initiative (ESA-CCI) satellite data or with fewer people than ten per 1 × 1-km pixel based on WorldPop estimates are masked in grey[15,26].

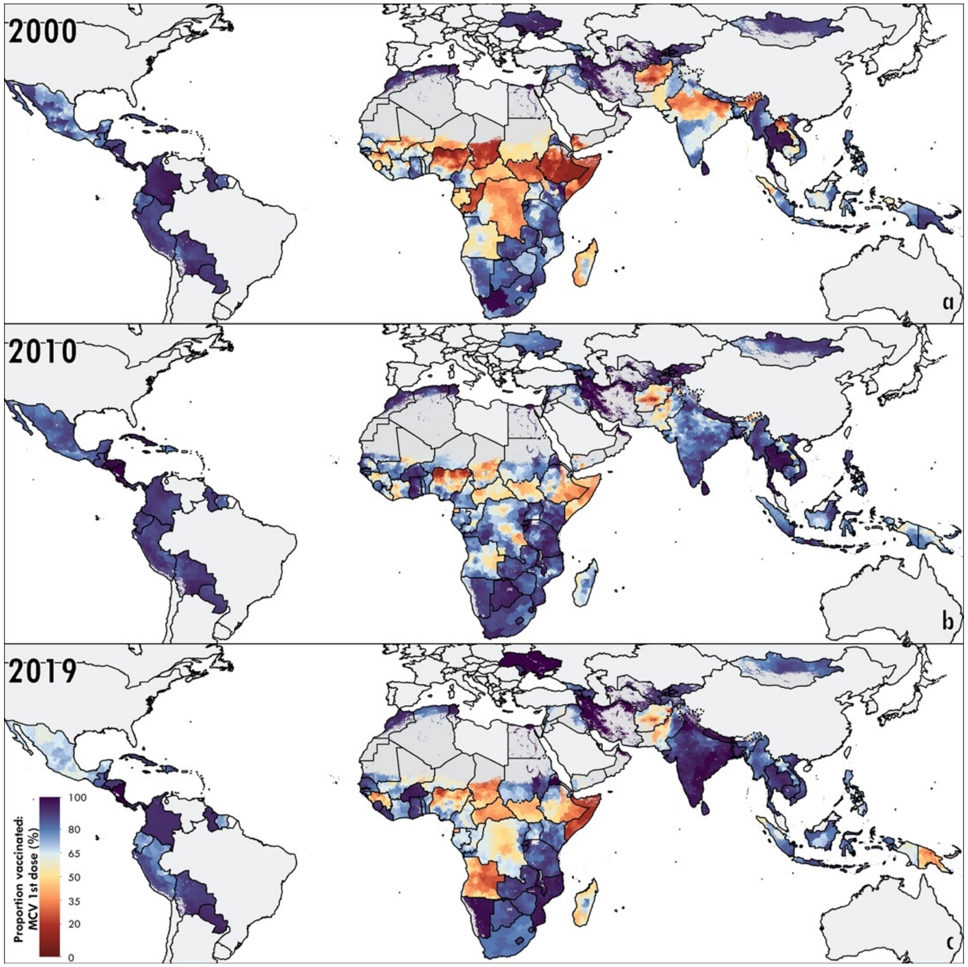


*Supplementary Fig. 19:* Estimated first-dose measles-containing vaccine (MCV1) coverage among districts in 101 low- and middle-income countries, 2000–2019, target age and t+1 cohort model.
MCV1 coverage among target population in only the target age and t+1 cohorts in districts in 2000 (a), 2010 (b), and 2019 (c). Countries excluded from the analysis and pixels classified as 'barren or sparsely vegetated' based on European Space Agency Climate Change Initiative (ESA-CCI) satellite data or with fewer people than ten per 1 × 1-km pixel based on WorldPop estimates are masked in grey[15,26].

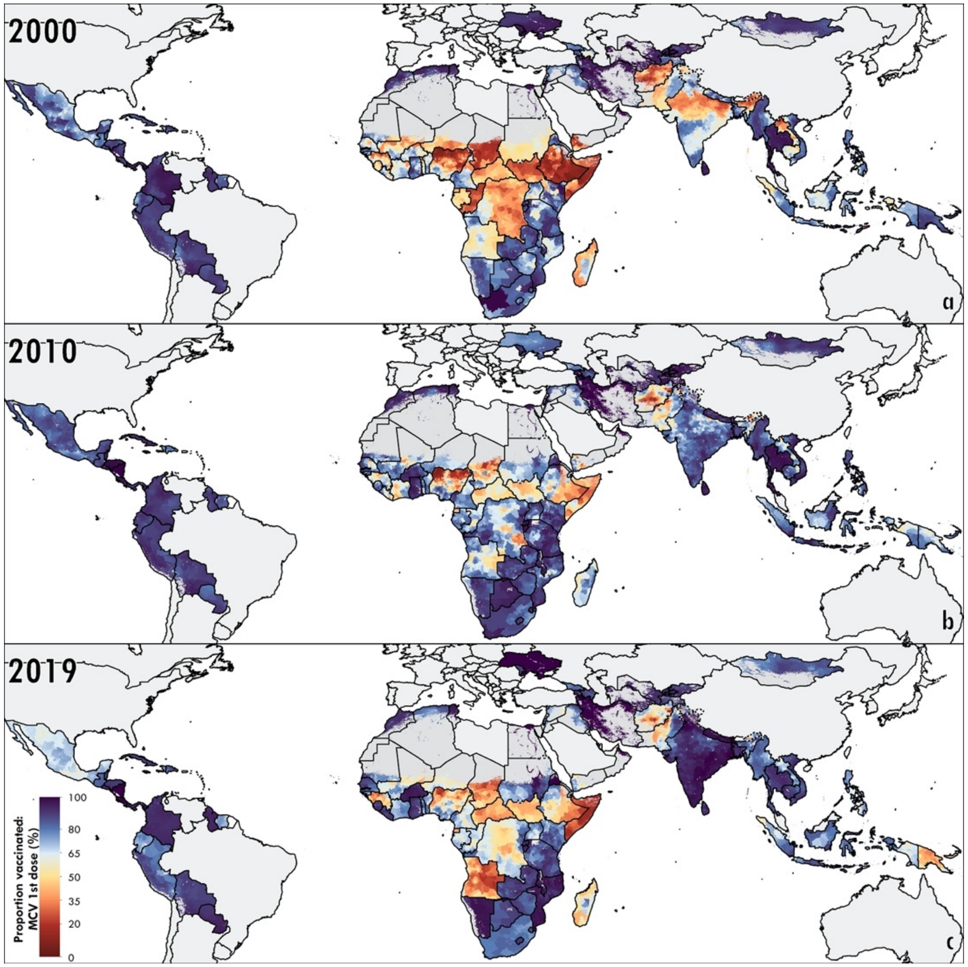


*Supplementary Fig. 20:* Estimated first-dose measles-containing vaccine (MCV1) coverage among districts in 101 low- and middle-income countries, 2000–2019, target age cohort only model.
MCV1 coverage among target population in only the target age cohort only in districts in 2000 (a), 2010 (b), and 2019 (c). Countries excluded from the analysis and pixels classified as 'barren or sparsely vegetated' based on European Space Agency Climate Change Initiative (ESA-CCI) satellite data or with fewer people than ten per 1 × 1-km pixel based on WorldPop estimates are masked in grey[15,26].

## 4.0 Supplementary Tables

*Supplementary Table 1:* GATHER compliance checklist

| **Item number** | **Checklist item** | **Reported on page number(s):** |
|---|---|---|
| **Objectives and funding** | | |
| 1 | Define the indicator(s), populations (including age, sex, and geographic entities), and time period(s) for which estimates were made. | Methods (Overview); Supplementary Section 1.2 |
| 2 | List the funding sources for the work. | Acknowledgements |
| **Data inputs** | | |
| *For all data inputs from multiple sources that are synthesised as part of the study:* | | |
| 3 | Describe how the data were identified and how the data were accessed. | Methods (Data); Supplementary Information 1.3 |
| 4 | Specify the inclusion and exclusion criteria. Identify all ad hoc exclusions. | Methods (Data); Supplementary Information 1.3 |
| 5 | Provide information on all included data sources and their main characteristics. For each data source used, report reference information or contact name/institution, population represented, data collection method, year(s) of data collection, sex and age range, diagnostic criteria or measurement method, and sample size, as relevant. | Supplementary Information 1.3; Supplementary Table 4 |
| 6 | Identify and describe any categories of input data that have potentially important biases (e.g., based on characteristics listed in item 5). | Supplementary Table 4 |
| *For data inputs that contribute to the analysis but were not synthesised as part of the study:* | | |
| 7 | Describe and give sources for any other data inputs. | Supplementary Tables 6-7 |
| *For all data inputs:* | | |
| 8 | Provide all data inputs in a file format from which data can be efficiently extracted (e.g., a spreadsheet rather than a PDF), including all relevant meta-data listed in item 5. For any data inputs that cannot be shared because of ethical or legal reasons, such as third-party ownership, provide a contact name or the name of the institution that retains the right to the data. | Supplementary Table 4; http://ghdx.healthdata.org/lbd-publication-data-input-sources |
| **Data analysis** | | |
| 9 | Provide a conceptual overview of the data analysis method. A diagram might be helpful. | Methods (Overview); Supplementary Information 1.4; Supplementary Fig. 1 |
| 10 | Provide a detailed description of all steps of the analysis, including mathematical formulae. This description should cover, as relevant, data cleaning, data pre-processing, data adjustments and weighting of data sources, and mathematical or statistical model(s). | Methods (Geostatistcial model; Post-estimation; Model validation; Post hoc inequality analyses); Supplementary Information 1.3 – 1.4 |
| 11 | Describe how candidate models were evaluated and how the final model(s) were selected. | Methods (Validation) |

| | | |
|---|---|---|
| 12 | Provide the results of an evaluation of model performance, if done, as well as the results of any relevant sensitivity analysis. | Methods (Validation); Supplementary Figs. 6-13; Supplementary Tables 9-12 |
| 13 | Describe methods for calculating uncertainty of the estimates. State which sources of uncertainty were, and were not, accounted for in the uncertainty analysis. | Methods (Post-estimation) |
| 14 | State how analytic or statistical source used to generate estimates can be accessed. | Data availability; Code availability |
| **Results and discussion** | | |
| 15 | Provide published estimates in a file format from which data can be efficiently extracted. | Data availability |
| 16 | Report a quantitative measure of uncertainty of the estimates (e.g., uncertainty intervals). | Main text (Tracking uneven progress in routine MCV1 coverage); Supplementary Figs. 14-16, 17 |
| 17 | Interpret results in light of existing evidence. If updating a previous set of estimates, describe the reasons for changes in estimates. | Discussion |
| 18 | Discuss limitations that affect interpretation of the estimates. | Methods (Limitations) |

*Supplementary Table 2:* MCV1 vaccination schedule by country
Immunisation schedule for MCV1 per WHO record, for each country in analysis[27].

| Country | Age |
|---|---|
| **Afghanistan** | 9 months |
| **Algeria** | 11 months |
| **Angola** | 9 months |
| **Armenia** | 12 months |
| **Azerbaijan** | 12 months |
| **Bangladesh** | 9 months |
| **Belize** | 12 months |
| **Benin** | 9 months |
| **Bhutan** | 9 months |
| **Bolivia** | 12 months |
| **Botswana** | 9 months |
| **Burkina Faso** | 9 months |
| **Burundi** | 9 months |
| **Cambodia** | 9 months |
| **Cameroon** | 9 months |
| **Cape Verde** | 9 months |
| **Central African Republic** | 9 months |
| **Chad** | 9 months |
| **Colombia** | 12 months |
| **Comoros** | 9 months |
| **Costa Rica** | 15 months |
| **Côte d'Ivoire** | 9 months |
| **Cuba** | 12 months |
| **Democratic Republic of the Congo** | 9 months |
| **Djibouti** | 9 months |
| **Dominican Republic** | 12 months |
| **Ecuador** | 12 months |

| | |
|---|---|
| **Egypt** | 12 months |
| **El Salvador** | 12 months |
| **Equatorial Guinea** | 9 months |
| **Eritrea** | 9 months |
| **Ethiopia** | 9 months |
| **Gabon** | 9 months |
| **Gambia** | 9 months |
| **Ghana** | 18 months |
| **Guatemala** | 12 months |
| **Guinea** | 9 months |
| **Guinea-Bissau** | 9 months |
| **Guyana** | 12 months |
| **Haiti** | 9 months |
| **Honduras** | 12 months |
| **India** | 9 months |
| **Indonesia** | 9 months |
| **Iran** | 12 months |
| **Iraq** | 9 months |
| **Jamaica** | 12 months |
| **Jordan** | 9 months |
| **Kenya** | 9 months |
| **Kyrgyzstan** | 12 months |
| **Laos** | 9 months |
| **Lesotho** | 9 months |
| **Liberia** | 9 months |
| **Madagascar** | 9 months |
| **Malawi** | 9 months |
| **Malaysia** | 6 months |
| **Mali** | 9 months |

| | |
|---|---|
| **Mauritania** | 9 months |
| **Mexico** | 12 months |
| **Moldova** | 12 months |
| **Mongolia** | 9 months |
| **Morocco** | 9 months |
| **Mozambique** | 9 months |
| **Myanmar** | 9 months |
| **Namibia** | 9 months |
| **Nepal** | 9 months |
| **Nicaragua** | 12 months |
| **Niger** | 9 months |
| **Nigeria** | 9 months |
| **Pakistan** | 9 months |
| **Papua New Guinea** | 9 months |
| **Paraguay** | 12 months |
| **Peru** | 12 months |
| **Philippines** | 9 months |
| **Republic of Congo** | 8 months |
| **Rwanda** | 9 months |
| **Sâo Tomé and Príncipe** | 9 months |
| **Senegal** | 9 months |
| **Sierra Leone** | 9 months |
| **Somalia** | 9 months |
| **South Africa** | 6 months |
| **South Sudan** | 9 months |
| **Sri Lanka** | 9 months |
| **Sudan** | 9 months |
| **Suriname** | 12 months |
| **Swaziland** | 9 months |

| | |
|---|---|
| **Syria** | 6 months |
| **Tajikistan** | 12 months |
| **Tanzania** | 9 months |
| **Thailand** | 9 months |
| **Timor-Leste** | 9 months |
| **Togo** | 9 months |
| **Trinidad and Tobago** | 12 months |
| **Turkmenistan** | 12 months |
| **Uganda** | 9 months |
| **Ukraine** | 12 months |
| **Uzbekistan** | 12 months |
| **Vietnam** | 9 months |
| **Yemen** | 9 months |
| **Zambia** | 9 months |
| **Zimbabwe** | 9 months |

*Supplementary Table 3:* List of countries considered for analysis stratified by Socio-Demographic Index (SDI) level and Decade of Vaccine (DoV) status

Countries included in analyses, stratified by SDI are listed. Countries not included in low, low-middle, or middle SDI classifications but are included in DoV priority countries are also listed[9,10]. All countries included in the DoV priority list are indicated by *.

| **Low SDI (0 – 0.455)** | **Low-middle SDI (0.455 – 0.606)** | **Middle SDI (0.606 – 0.688)** | **DoV priority** |
|---|---|---|---|
| Afghanistan* | Angola* | Algeria | Armenia* |
| Benin* | Bangladesh* | Botswana | Azerbaijan* |
| Burkina Faso* | Belize* | Cuba* | Iran* |
| Burundi* | Bolivia* | Colombia | Malaysia* |
| Central African Republic* | Cambodia* | Costa Rica | Moldova* |
| Chad* | Cameroon* | Ecuador | Turkmenistan* |
| Comoros* | Cape Verde | Egypt* | Ukraine* |
| Côte d'Ivoire* | Djibouti* | Equatorial Guinea | |
| Democratic Republic of the Congo* | Dominican Republic | Gabon | |
| Eritrea* | El Salvador* | Guyana* | |
| Ethiopia* | Ghana* | Indonesia* | |
| Guinea* | Guatemala* | Iraq* | |
| Guinea-Bissau* | Honduras* | Jamaica | |
| Haiti* | India* | Mexico | |
| Liberia* | Honduras* | Mongolia* | |
| Madagascar* | India* | Namibia | |
| Malawi* | Kenya* | Panama | |
| Mali* | Kyrgyzstan* | Paraguay* | |
| Mozambique* | Laos* | Peru | |
| Nepal* | Lesotho* | South Africa | |
| Niger* | Mauritania* | Sri Lanka* | |
| Papua New Guinea* | Morocco* | Suriname | |
| Rwanda* | Myanmar* | Syria* | |
| Senegal* | Nicaragua* | Thailand | |
| Sierra Leone* | Nigeria* | Tunisia | |
| Somalia* | Pakistan* | Turkmenistan* | |
| South Sudan* | Philippines* | Uzbekistan* | |
| Tanzania* | Republic of the Congo* | Vietnam* | |
| The Gambia* | Sâo Tomé and Príncipe* | | |
| Togo* | Sudan* | | |
| Uganda* | Swaziland (eSwatini)* | | |
| Yemen* | Tajikistan* | | |
| | Timor-Leste* | | |
| | Zambia* | | |
| | Zimbabwe* | | |

*Supplementary Table 4:* Input sources included in final analysis

List of sources with GHDx identification number included in the final analysis along with number of GPS and areally located clusters is provided. These sources can also be explored following this link to the GHDx input data sources tool: http://ghdx.healthdata.org/lbd-publication-data-input-sources.

| GHDx id | Country | GPS-located clusters | Areally-located clusters | Number of children included | Series | Year(s) | Citation | Link |
|---|---|---|---|---|---|---|---|---|
| **18468** | Afghanistan | 0 | 27 | 1179 | Afghanistan Health Survey 2006 | 2006 | Indian Institute of Health Management Research (IIHMR), Johns Hopkins University, Ministry of Public Health (Afghanistan). Afghanistan Health Survey 2006. | GHDx |
| **157018** | Afghanistan | 0 | 309 | 18436 | Afghanistan Demographic and Health Survey 2015-2016 | 2015-2016 | Central Statistics Organization (Afghanistan), ICF International, Ministry of Public Health (Afghanistan). Afghanistan Demographic and Health Survey 2015-2016. Fairfax, United States of America: ICF International, 2017. | GHDx |
| **627** | Algeria | 0 | 42 | 195 | Algeria Family Health Survey 2002-2003 | 2002-2003 | National Office of Statistics (Algeria), Ministry of Health, Population and Hospital Reform (Algeria), League of Arab States. Algeria Family Health Survey 2002-2003. | GHDx |
| **210614** | Algeria | 0 | 7 | 8761 | Algeria Multiple Indicator Cluster Survey 2012-2013 | 2012-2013 | Ministry of Health and Population (Algeria), United Nations Children's Fund (UNICEF). Algeria Multiple Indicator Cluster Survey 2012-2013. New York, United States of America: United Nations Children's Fund (UNICEF), 2018. | GHDx |
| **687** | Angola | 0 | 18 | 1054 | Angola Multiple Indicator Cluster Survey 2001 | 2001 | National Institute of Statistics (Angola), United Nations Children's Fund (UNICEF). Angola Multiple Indicator Cluster Survey 2001. New York, United States of America: United Nations Children's Fund (UNICEF). | GHDx |
| **30394** | Angola | 0 | 18 | 4840 | Angola Integrated Inquiry into People's Well-Being 2008-2009 | 2008-2009 | National Institute of Statistics (Angola), Oxford Policy Management, United Nations Children's Fund (UNICEF). Angola Integrated Inquiry into People's Well-Being 2008-2009. | GHDx |

| | | | | | | | | |
|---|---|---|---|---|---|---|---|---|
| **218555** | Angola | 624 | 0 | 5841 | Angola Demographic and Health Survey 2015-2016 | 2015-2016 | ICF International, Ministry of Health (Angola), National Institute of Statistics (Angola), United Nations Children's Fund (UNICEF). Angola Demographic and Health Survey 2015-2016. Fairfax, United States of America: ICF International, 2017. | GHDx |
| **31750** | Armenia | 269 | 0 | 1357 | Armenia Demographic and Health Survey 2010 | 2010 | ICF Macro, Ministry of Health (Armenia), National Statistical Service of the Republic of Armenia. Armenia Demographic and Health Survey 2010. Fairfax, United States of America: ICF International, 2015. | GHDx |
| **218563** | Armenia | 198 | 0 | 568 | Armenia Demographic and Health Survey 2015-2016 | 2015-2016 | ICF International, Ministry of Health (Armenia), National Statistical Service of the Republic of Armenia. Armenia Demographic and Health Survey 2015-2016. Fairfax, United States of America: ICF International, 2017. | GHDx |
| **18865** | Azerbaijan | 0 | 9 | 1586 | Azerbaijan Demographic and Health Survey 2006 | 2006 | Macro International, Inc, State Statistical Committee of Azerbaijan. Azerbaijan Demographic and Health Survey 2006. Fairfax, United States of America: ICF International. | GHDx |
| **951** | Bangladesh | 1813 | 0 | 17878 | Bangladesh Multiple Indicator Cluster Survey 2006 | 2006 | Bangladesh Bureau of Statistics (BBS), Mitra and Associates, United Nations Children's Fund (UNICEF). Bangladesh Multiple Indicator Cluster Survey 2006. New York, United States of America: United Nations Children's Fund (UNICEF). | GHDx |
| **18902** | Bangladesh | 359 | 0 | 4004 | Bangladesh Demographic and Health Survey 2004 | 2004 | Mitra and Associates, ORC Macro. Bangladesh Demographic and Health Survey 2004. Fairfax, United States of America: ICF International. | GHDx |
| **18913** | Bangladesh | 361 | 0 | 3641 | Bangladesh Demographic and Health Survey 2007 | 2007 | Macro International, Inc, Mitra and Associates, National Institute of Population Research and Training (NIPORT). Bangladesh Demographic and Health Survey 2007. Fairfax, United States of America: ICF International, 2009. | GHDx |
| **55956** | Bangladesh | 600 | 0 | 5069 | Bangladesh Demographic and Health Survey 2011-2012 | 2011-2012 | ICF Macro, Mitra and Associates, National Institute of Population Research and Training (NIPORT). Bangladesh Demographic and Health Survey 2011-2012. Calverton, United States of America: ICF Macro. | GHDx |

| | | | | | | | | |
|---|---|---|---|---|---|---|---|---|
| **157021** | Bangladesh | 598 | 0 | 4583 | Bangladesh Demographic and Health Survey 2014 | 2014 | ICF International, Mitra and Associates, National Institute of Population Research and Training (NIPORT). Bangladesh Demographic and Health Survey 2014. Fairfax, United States of America: ICF International, 2015. | GHDx |
| **261683** | Bangladesh | 1 | 0 | 149 | Bangladesh - Dhaka Malnutrition and Enteric Disease Study 2009-2014 | 2009-2014 | Fogarty International Center, National Institutes of Health (NIH), Foundation for the National Institutes of Health (FNIH), International Centre for Diarrhoeal Disease Research, Bangladesh (ICDDR,B). Bangladesh - Dhaka Malnutrition and Enteric Disease Study 2009-2014. | GHDx |
| **1089** | Belize | 0 | 6 | 452 | Belize Multiple Indicator Cluster Survey 2006 | 2006 | Statistical Institute of Belize, United Nations Children's Fund (UNICEF). Belize Multiple Indicator Cluster Survey 2006. New York, United States of America: United Nations Children's Fund (UNICEF). | GHDx |
| **76699** | Belize | 0 | 6 | 943 | Belize Multiple Indicator Cluster Survey 2011 | 2011 | Statistical Institute of Belize, United Nations Children's Fund (UNICEF). Belize Multiple Indicator Cluster Survey 2011. New York, United States of America: United Nations Children's Fund (UNICEF), 2013. | GHDx |
| **264910** | Belize | 0 | 6 | 512 | Belize Multiple Indicator Cluster Survey 2015-2016 | 2015-2016 | Government of Belize, Statistical Institute of Belize, UN Resident Coordinator Fund (UN ResCor), United Nations Children's Fund (UNICEF), United Nations Development Programme (UNDP). Belize Multiple Indicator Cluster Survey 2015-2016. New York, United States of America: United Nations Children's Fund (UNICEF), 2018. | GHDx |
| **18950** | Benin | 225 | 0 | 923 | Benin Demographic and Health Survey 2001 | 2001 | National Institute of Statistics and Economic Analysis (INSAE) (Benin), ORC Macro. Benin Demographic and Health Survey 2001. Fairfax, United States of America: ICF International. | GHDx |
| **18959** | Benin | 0 | 12 | 8518 | Benin Demographic and Health Survey 2006 | 2006 | Macro International, Inc, National Institute of Statistics and Economic Analysis (INSAE) (Benin), National Program Against AIDS (PNLS) (Benin). Benin Demographic and Health Survey 2006. Fairfax, United States of America: ICF International. | GHDx |

| | | | | | | | | |
|---|---|---|---|---|---|---|---|---|
| **206075** | Benin | 0 | 12 | 4820 | Benin Multiple Indicator Cluster Survey 2014 | 2014 | National Institute of Statistics and Economic Analysis (INSAE) (Benin), United Nations Children's Fund (UNICEF). Benin Multiple Indicator Cluster Survey 2014. New York, United States of America: United Nations Children's Fund (UNICEF), 2017. | GHDx |
| **218565** | Benin | 539 | 0 | 4720 | Benin Demographic and Health Survey 2017-2018 | 2017-2018 | Hubert Koutoukou Maga National University Hospital Center (CNHU-HKM)(Benin), ICF International, National Institute of Statistics and Economic Analysis (INSAE) (Benin), National Malaria Control Program, Ministry of Health (Benin), Permanent Secretariat of the Food Council and Nutrition (SP-CAN)(Benin). Benin Demographic and Health Survey 2017-2018. Fairfax, United States of America: ICF International, 2018. | GHDx |
| **19001** | Bolivia | 0 | 8 | 4216 | Bolivia Demographic and Health Survey 2003-2004 | 2003-2004 | Macro International, Inc, Ministry of Health and Sports (Bolivia), National Institute of Statistics (Bolivia). Bolivia Demographic and Health Survey 2003-2004. Fairfax, United States of America: ICF International. | GHDx |
| **19016** | Bolivia | 965 | 0 | 5134 | Bolivia Demographic and Health Survey 2008 | 2008 | Macro International, Inc, Ministry of Health and Sports (Bolivia), National Institute of Statistics (Bolivia). Bolivia Demographic and Health Survey 2008. Fairfax, United States of America: ICF International. | GHDx |
| **323944** | Bolivia | 0 | 9 | 1834 | Bolivia Demographic and Health Survey 2016 | 2016 | Ministry of Health (Bolivia), National Institute of Statistics (Bolivia). Bolivia Demographic and Health Survey 2016. La Paz, Bolivia: National Institute of Statistics (Bolivia), 2017. | GHDx |
| **22125** | Botswana | 0 | 292 | 1183 | Botswana Family Health Survey 2007-2008 | 2007-2008 | Central Statistics Office (Botswana). Botswana Family Health Survey 2007-2008. Gaborone, Botswana: Central Statistics Office (Botswana), 2009. | GHDx |
| **1927** | Burkina Faso | 195 | 0 | 3185 | Burkina Faso Multiple Indicator Cluster Survey 2006 | 2006 | National Institute of Statistics and Demography (Burkina Faso), United Nations Children's Fund (UNICEF). Burkina Faso Multiple Indicator Cluster Survey 2006. New York, United States of America: United Nations Children's Fund (UNICEF). | GHDx |

| | | | | | | | | |
|---|---|---|---|---|---|---|---|---|
| **19088** | Burkina Faso | 397 | 0 | 5361 | Burkina Faso Demographic and Health Survey 2003 | 2003 | Macro International, Inc, National Institute of Statistics and Demography (Burkina Faso). Burkina Faso Demographic and Health Survey 2003. Fairfax, United States of America: ICF International. | GHDx |
| **19133** | Burkina Faso | 541 | 0 | 7730 | Burkina Faso Demographic and Health Survey 2010-2011 | 2010-2011 | ICF Macro, Ministry of Health (Burkina Faso), National Institute of Statistics and Demography (Burkina Faso). Burkina Faso Demographic and Health Survey 2010-2011. Fairfax, United States of America: ICF International. | GHDx |
| **26642** | Burkina Faso | 0 | 13 | 4193 | Burkina Faso Global Fund Household Health Coverage Survey 2008 | 2008 | Global Fund to Fight Aids Tuberculosis and Malaria (GFATM). Burkina Faso Global Fund Household Health Coverage Survey 2008. | GHDx |
| **236156** | Burkina Faso | 0 | 13 | 7148 | Burkina Faso Continuous Multisectoral Survey 2014 | 2014 | National Institute of Statistics and Demography (Burkina Faso), World Bank. Burkina Faso Continuous Multisectoral Survey 2014. Washington DC, United States of America: World Bank. | GHDx |
| **1981** | Burundi | 0 | 524 | 3924 | Burundi Multiple Indicator Cluster Survey 2005 | 2005 | United Nations Children's Fund (UNICEF), Burundi Institute of Statistics and Economic Studies, United Nations Population Fund (UNFPA). Burundi Multiple Indicator Cluster Survey 2005. New York, United States: United Nations Children's Fund (UNICEF). | GHDx |
| **30431** | Burundi | 375 | 0 | 4955 | Burundi Demographic and Health Survey 2010-2011 | 2010-2011 | Burundi Institute of Statistics and Economic Studies, ICF International, Ministry of Public Health and the Fight Against AIDS (Burundi). Burundi Demographic and Health Survey 2010-2011. Fairfax, United States of America: ICF International, 2012. | GHDx |
| **286766** | Burundi | 542 | 0 | 3960 | Burundi Demographic and Health Survey 2016-2017 | 2016-2017 | Burundi Institute of Statistics and Economic Studies, ICF International, Ministry of Public Health and the Fight Against AIDS (Burundi). Burundi Demographic and Health Survey 2016-2017. Fairfax, United States of America: ICF International, 2018. | GHDx |
| **19167** | Cambodia | 548 | 0 | 4465 | Cambodia Demographic | 2005-2006 | Macro International, Inc, National Institute of Public Health (Cambodia), National Institute of Statistics | GHDx |

| | | | | | | | | |
|---|---|---|---|---|---|---|---|---|
| | | | | | and Health Survey 2005-2006 | | (Cambodia). Cambodia Demographic and Health Survey 2005-2006. Fairfax, United States of America: ICF International. | |
| **30379** | Cambodia | 607 | 0 | 4726 | Cambodia Demographic and Health Survey 2010-2011 | 2010-2011 | ICF Macro, Ministry of Health (Cambodia), National Institute of Statistics (Cambodia). Cambodia Demographic and Health Survey 2010-2011. Fairfax, United States of America: ICF International. | GHDx |
| **30963** | Cambodia | 239 | 16 | 763 | Cambodia Socio-Economic Survey 2003-2005 | 2003-2005 | National Institute of Statistics (Cambodia), Statistics Sweden. Cambodia Socio-Economic Survey 2003-2005. Phnom Penh, Cambodia: National Institute of Statistics (Cambodia). | GHDx |
| **157024** | Cambodia | 609 | 0 | 4123 | Cambodia Demographic and Health Survey 2014 | 2014 | ICF International, Ministry of Health (Cambodia), National Institute of Statistics (Cambodia). Cambodia Demographic and Health Survey 2014. Fairfax, United States of America: ICF International, 2017. | GHDx |
| **2039** | Cameroon | 0 | 12 | 1403 | Cameroon Household Survey 2001 | 2001 | National Institute of Statistics (Cameroon), Directorate of Statistics and National Accounts, Ministry of Economics and Finance (Cameroon), AFRISTAT. Cameroon Household Survey 2001. Yaounde, Cameroon: National Institute of Statistics (Cameroon). | GHDx |
| **2063** | Cameroon | 0 | 218 | 3661 | Cameroon Multiple Indicator Cluster Survey 2006 | 2006 | United Nations Children's Fund (UNICEF), National Institute of Statistics (Cameroon). Cameroon Multiple Indicator Cluster Survey 2006. New York, United States: United Nations Children's Fund (UNICEF). | GHDx |
| **19211** | Cameroon | 458 | 0 | 4790 | Cameroon Demographic and Health Survey 2004 | 2004 | Macro International, Inc, National Institute of Statistics (Cameroon). Cameroon Demographic and Health Survey 2004. Fairfax, United States of America: ICF International. | GHDx |
| **19274** | Cameroon | 576 | 0 | 7174 | Cameroon Demographic and Health Survey 2011 | 2011 | ICF International, Ministry of Economy, Planning and Regional Development (Cameroon), Ministry of Public Health (Cameroon), National Institute of Statistics (Cameroon), Pasteur Center of Cameroon. Cameroon | GHDx |

| | | | | | | | | |
|---|---|---|---|---|---|---|---|---|
| | | | | | | | Demographic and Health Survey 2011. Fairfax, United States of America: ICF International. | |
| **244455** | Cameroon | 0 | 12 | 1552 | Cameroon Multiple Indicator Cluster Survey 2014 | 2014 | Ministry of Public Health (Cameroon), National Institute of Statistics (Cameroon), United Nations Children's Fund (UNICEF). Cameroon Multiple Indicator Cluster Survey 2014. New York, United States of America: United Nations Children's Fund (UNICEF), 2017. | GHDx |
| **413167** | Cameroon | 429 | 0 | 3550 | Cameroon Demographic and Health Survey 2018-2019 | 2018-2019 | ICF International, Ministry of Public Health (Cameroon), National Institute of Statistics (Cameroon). Cameroon Demographic and Health Survey 2018-2019. Fairfax, United States of America: ICF International. | GHDx |
| **21442** | Cape Verde | 0 | 9 | 436 | Cape Verde Demographic and Health Survey 2005 | 2005 | Macro International, Inc, Ministry of Health (Cape Verde), National Institute of Statistics (Cape Verde). Cape Verde Demographic and Health Survey 2005. | GHDx |
| **2223** | Central African Republic | 0 | 16 | 7446 | Central African Republic Multiple Indicator Cluster Survey 2006 | 2006 | United Nations Children's Fund (UNICEF). Central African Republic Multiple Indicator Cluster Survey 2006. New York, United States: United Nations Children's Fund (UNICEF). | GHDx |
| **82832** | Central African Republic | 0 | 17 | 8245 | Central African Republic Multiple Indicator Cluster Survey 2010-2011 | 2010-2011 | Central African Institute of Statistics, Economic and Social Studies (ICASEES) (Central African Republic), ICF International. Central African Republic Multiple Indicator Cluster Survey 2010-2011. Fairfax, United States of America: ICF International, 2013. | GHDx |
| **19315** | Chad | 0 | 9 | 3346 | Chad Demographic and Health Survey 2004 | 2004 | Macro International, Inc, National Institute for Statistics, Economic and Demographic Studies (INSEED) (Chad). Chad Demographic and Health Survey 2004. Fairfax, United States of America: ICF International. | GHDx |
| **76701** | Chad | 0 | 60 | 11799 | Chad Multiple Indicator | 2010 | Ministry of Planning, Economy, and International Cooperation (Chad), National Institute for Statistics, | GHDx |

| | | | | | | | | |
|---|---|---|---|---|---|---|---|---|
| | | | | | Cluster Survey 2010 | | Economic and Demographic Studies (INSEED) (Chad), United Nations Children's Fund (UNICEF). Chad Multiple Indicator Cluster Survey 2010. New York, United States of America: United Nations Children's Fund (UNICEF), 2014. | |
| **157025** | Chad | 624 | 0 | 11687 | Chad Demographic and Health Survey 2014-2015 | 2014-2015 | ICF International, National Institute for Statistics, Economic and Demographic Studies (INSEED) (Chad). Chad Demographic and Health Survey 2014-2015. Fairfax, United States of America: ICF International, 2016. | GHDx |
| **19324** | Colombia | 0 | 33 | 9349 | Colombia Demographic and Health Survey 2004-2005 | 2004-2005 | Macro International, Inc, Profamilia (Colombia). Colombia Demographic and Health Survey 2004-2005. Fairfax, United States of America: ICF International, 2005. | GHDx |
| **21281** | Colombia | 3972 | 0 | 12067 | Colombia Demographic and Health Survey 2009-2010 | 2009-2010 | ICF Macro, Profamilia (Colombia). Colombia Demographic and Health Survey 2009-2010. Fairfax, United States of America: ICF International, 2011. | GHDx |
| **76850** | Comoros | 239 | 0 | 1672 | Comoros Demographic and Health Survey 2012-2013 | 2012-2013 | General Directorate of Statistics and Forecasting (Comoros), ICF International. Comoros Demographic and Health Survey 2012-2013. Fairfax, United States of America: ICF International. | GHDx |
| **19391** | Congo | 0 | 12 | 968 | Congo Demographic and Health Survey 2005 | 2005 | Macro International, Inc, National Center for Statistics and Economic Studies (Congo, Rep.). Congo Demographic and Health Survey 2005. Fairfax, United States of America: ICF International. | GHDx |
| **56151** | Congo | 0 | 12 | 5702 | Congo Demographic and Health Survey 2011-2012 | 2011-2012 | ICF International, Ministry of Health (Congo, Rep.), National Center for Statistics and Economic Studies (Congo, Rep.). Congo Demographic and Health Survey 2011-2012. Fairfax, United States of America: ICF International. | GHDx |
| **234733** | Congo | 0 | 11 | 3764 | Congo Multiple | 2014-2015 | National Institute of Statistics (INS) (Congo, Rep.), United Nations Children's Fund (UNICEF). Congo Multiple | GHDx |

| | | | | | | | | |
|---|---|---|---|---|---|---|---|---|
| | | | | | Indicator Cluster Survey 2014-2015 | | Indicator Cluster Survey 2014-2015. New York, United States of America: United Nations Children's Fund (UNICEF), 2018. | |
| **125596** | Costa Rica | 0 | 7 | 1349 | Costa Rica Multiple Indicator Cluster Survey 2011 | 2011 | Costa Rican Demographic Association, Ministry of Health (Costa Rica), United Nations Children's Fund (UNICEF). Costa Rica Multiple Indicator Cluster Survey 2011. New York, United States of America: United Nations Children's Fund (UNICEF), 2013. | GHDx |
| **164089** | Costa Rica | 2 | 0 | 2 | Costa Rica Survey of Family Health Services and Expenses 2008 | 2008 | Central American Population Center, University of Costa Rica. Costa Rica Survey of Family Health Services and Expenses 2008. San José, Costa Rica: Central American Population Center, University of Costa Rica. | GHDx |
| **18533** | Cote d'Ivoire | 341 | 0 | 4017 | Côte d'Ivoire Demographic and Health Survey 2011-2012 | 2011-2012 | ICF International, Ministry of the Fight Against AIDS (Côte d'Ivoire), National Institute of Statistics (Côte d'Ivoire). Côte d'Ivoire Demographic and Health Survey 2011-2012. Fairfax, United States of America: ICF International. | GHDx |
| **26433** | Cote d'Ivoire | 0 | 11 | 3587 | Côte d'Ivoire Multiple Indicator Cluster Survey 2006 | 2006 | United Nations Children's Fund (UNICEF), National Institute of Statistics (Côte d'Ivoire). Côte d'Ivoire Multiple Indicator Cluster Survey 2006. New York, United States: United Nations Children's Fund (UNICEF). | GHDx |
| **218611** | Cote d'Ivoire | 0 | 11 | 3003 | Cote d'Ivoire Multiple Indicator Cluster Survey 2016 | 2016 | National Institute of Statistics (Côte d'Ivoire), United Nations Children's Fund (UNICEF). Cote d'Ivoire Multiple Indicator Cluster Survey 2016. New York, United States of America: United Nations Children's Fund (UNICEF), 2018. | GHDx |
| **3310** | Cuba | 0 | 4 | 1840 | Cuba Multiple Indicator Cluster Survey 2006 | 2006 | United Nations Children's Fund (UNICEF), National Bureau of Statistics (Cuba). Cuba Multiple Indicator Cluster Survey 2006. New York, United States: United Nations Children's Fund (UNICEF). | GHDx |
| **60935** | Cuba | 0 | 15 | 3155 | Cuba Multiple Indicator Cluster Survey 2010-2011 | 2010-2011 | Ministry of Public Health (Cuba), United Nations Children's Fund (UNICEF). Cuba Multiple Indicator Cluster Survey 2010-2011. New York, United States of America: United Nations Children's Fund (UNICEF). | GHDx |

| | | | | | | | | |
|---|---|---|---|---|---|---|---|---|
| **169975** | Cuba | 0 | 2 | 1214 | Cuba Multiple Indicator Cluster Survey 2014 | 2014 | Ministry of Public Health (Cuba), National Office of Statistics (Cuba), United Nations Children's Fund (UNICEF). Cuba Multiple Indicator Cluster Survey 2014. New York, United States of America: United Nations Children's Fund (UNICEF), 2005. | GHDx |
| **3161** | Democratic Republic of the Congo | 0 | 11 | 2520 | Democratic Republic of the Congo Multiple Indicator Cluster Survey 2001 | 2001 | Ministry of Planning and Reconstruction (Congo, DR), United Nations Children's Fund (UNICEF). Congo, DR Multiple Indicator Cluster Survey 2001. New York, United States: United Nations Children's Fund (UNICEF). | GHDx |
| **19381** | Democratic Republic of the Congo | 293 | 0 | 4552 | Democratic Republic of the Congo Demographic and Health Survey 2007 | 2007 | Macro International, Inc, Ministry of Planning (Congo, DR). Democratic Republic of the Congo Demographic and Health Survey 2007. Fairfax, United States of America: ICF International. | GHDx |
| **26998** | Democratic Republic of the Congo | 357 | 0 | 6204 | Democratic Republic of the Congo Multiple Indicator Cluster Survey 2010 | 2010 | National Statistical Institute (Congo, DR), Ministry of Planning (Congo, DR), United Nations Children's Fund (UNICEF). Congo, DR Multiple Indicator Cluster Survey 2010. New York, United States: United Nations Children's Fund (UNICEF). | GHDx |
| **76878** | Democratic Republic of the Congo | 492 | 0 | 9472 | Democratic Republic of the Congo Demographic and Health Survey 2013-2014 | 2013-2014 | ICF International, Ministry of Planning and Monitoring Implementation of the Revolution of Modernity (Congo, DR), Ministry of Public Health (Congo, DR), National Institute of Statistics (Congo, DR). Democratic Republic of the Congo Demographic and Health Survey 2013-2014. Fairfax, United States of America: ICF International, 2014. | GHDx |
| **437955** | Democratic Republic of the Congo | 0 | 26 | 7970 | Democratic Republic of the Congo Multiple | 2017-2018 | National Statistical Institute (Congo, DR), Ministry of Planning (Congo, DR), United Nations Children's Fund (UNICEF). Congo, DR Multiple Indicator Cluster Survey | GHDx |

| | | | | | | | | |
|---|---|---|---|---|---|---|---|---|
| | | | | | Indicator Cluster Survey 2017-2018 | | 2010. New York, United States: United Nations Children's Fund (UNICEF). | |
| **3392** | Djibouti | 0 | 1 | 470 | Djibouti Family Health Survey 2002 | 2002 | Department of Statistics and Demographic Studies (Djibouti), League of Arab States, Ministry of Health (Djibouti), Pan Arab Project for Family Health (PAPFAM). Djibouti Family Health Survey 2002. | GHDx |
| **3404** | Djibouti | 35 | 1 | 1362 | Djibouti Multiple Indicator Cluster Survey 2006 | 2006 | Ministry of Economy, Finance, and Planning in charge of Privatization (Djibouti), Ministry of Health (Djibouti), United Nations Children's Fund (UNICEF). Djibouti Multiple Indicator Cluster Survey 2006. New York, United States of America: United Nations Children's Fund (UNICEF). | GHDx |
| **218035** | Djibouti | 0 | 6 | 732 | Djibouti Family Health Survey 2012 | 2012 | Department of Statistics and Demographic Studies (Djibouti), League of Arab States, Ministry of Health (Djibouti), Pan Arab Project for Family Health (PAPFAM). Djibouti Family Health Survey 2012. | GHDx |
| **3441** | Dominican Republic | 0 | 32 | 2630 | Dominican Republic National Multipurpose Household Survey 2007 | 2007 | National Statistics Office (Dominican Republic). Dominican Republic National Multipurpose Household Survey 2007. Santo Domingo, Dominican Republic: National Statistics Office (Dominican Republic). | GHDx |
| **3455** | Dominican Republic | 0 | 32 | 1195 | Dominican Republic National Multipurpose Household Survey 2006 | 2006 | National Statistics Office (Dominican Republic), United Nations Children's Fund (UNICEF). Dominican Republic National Multipurpose Household Survey 2006. Santo Domingo, Dominican Republic: National Statistics Office (Dominican Republic). | GHDx |
| **19444** | Dominican Republic | 0 | 32 | 2243 | Dominican Republic Demographic and Health Survey 2002 | 2002 | Center for Social and Demographic Studies (Dominican Republic) (CESDEM), Macro International, Inc. Dominican Republic Demographic and Health Survey 2002. Fairfax, United States of America: ICF International. | GHDx |
| **19456** | Dominican Republic | 1396 | 0 | 6410 | Dominican Republic | 2007 | Center for Social and Demographic Studies (Dominican Republic) (CESDEM), Macro International, Inc. | GHDx |

| | | | | | | | | |
|---|---|---|---|---|---|---|---|---|
| | | | | | Demographic and Health Survey 2007 | | Dominican Republic Demographic and Health Survey 2007. Fairfax, United States of America: ICF International. | |
| **21198** | Dominican Republic | 0 | 9 | 514 | Dominican Republic Special Demographic and Health Survey 2007 | 2007 | Center for Social and Demographic Studies (Dominican Republic) (CESDEM), Macro International, Inc. Dominican Republic Special Demographic and Health Survey 2007. Fairfax, United States of America: ICF International. | GHDx |
| **77819** | Dominican Republic | 503 | 0 | 2097 | Dominican Republic Demographic and Health Survey 2013 | 2013 | Center for Social and Demographic Studies (Dominican Republic) (CESDEM), ICF International, Ministry of Public Health and Social Assistance (Dominican Republic). Dominican Republic Demographic and Health Survey 2013. Fairfax, United States of America: ICF International, 2014. | GHDx |
| **165645** | Dominican Republic | 108 | 0 | 483 | Dominican Republic Special Demographic and Health Survey 2013 | 2013 | Center for Social and Demographic Studies (Dominican Republic) (CESDEM), ICF International, National Public Health Laboratory (Dominican Republic). Dominican Republic Special Demographic and Health Survey 2013. Fairfax, United States of America: ICF International, 2015. | GHDx |
| **200697** | Dominican Republic | 0 | 10 | 3872 | Dominican Republic Multiple Indicator Cluster Survey 2014 | 2014 | National Statistics Office (Dominican Republic), United Nations Children's Fund (UNICEF). Dominican Republic Multiple Indicator Cluster Survey 2014. New York, United States of America: United Nations Children's Fund (UNICEF), 2016. | GHDx |
| **46924** | Ecuador | 238 | 83 | 4184 | Ecuador Living Conditions Survey 2005-2006 | 2005-2006 | National Institute of Statistics and Censuses (Ecuador), Inter-American Development Bank (IDB). Ecuador Living Conditions Survey 2005-2006. Quito, Ecuador: National Institute of Statistics and Censuses (Ecuador). | GHDx |
| **153674** | Ecuador | 0 | 52 | 1411 | Ecuador National Health and | 2012 | Ministry of Public Health (Ecuador), National Institute of Statistics and Censuses (Ecuador). Ecuador National Health and Nutrition Survey 2012. | GHDx |

| | | | | | | | | |
|---|---|---|---|---|---|---|---|---|
| | | | | | Nutrition Survey 2012 | | | |
| **19521** | Egypt | 1264 | 0 | 7602 | Egypt Demographic and Health Survey 2005 | 2005 | El-Zanaty and Associates, Macro International, Inc, Ministry of Health and Population (Egypt), Population Council (Egypt). Egypt Demographic and Health Survey 2005. Fairfax, United States of America: ICF International. | GHDx |
| **19529** | Egypt | 763 | 0 | 2223 | Egypt Interim Demographic and Health Survey 2003 | 2003 | El-Zanaty and Associates, Macro International, Inc, Ministry of Health and Population (Egypt), Population Council (Egypt). Egypt Interim Demographic and Health Survey 2003. Fairfax, United States of America: ICF International. | GHDx |
| **26842** | Egypt | 1181 | 0 | 5834 | Egypt Demographic and Health Survey 2008 | 2008 | El-Zanaty and Associates, Macro International, Inc, Ministry of Health and Population (Egypt). Egypt Demographic and Health Survey 2008. Fairfax, United States of America: ICF International. | GHDx |
| **154897** | Egypt | 1714 | 0 | 11980 | Egypt Demographic and Health Survey 2014 | 2014 | El-Zanaty and Associates, ICF International, Ministry of Health and Population (Egypt). Egypt Demographic and Health Survey 2014. Fairfax, United States of America: ICF International. | GHDx |
| **200636** | El Salvador | 0 | 14 | 1562 | El Salvador Multiple Indicator Cluster Survey 2014 | 2014 | General Administration of Statistics and Censuses (El Salvador), Ministry of Health (El Salvador), United Nations Children's Fund (UNICEF). El Salvador Multiple Indicator Cluster Survey 2014. New York, United States of America: United Nations Children's Fund (UNICEF), 2017. | GHDx |
| **76884** | Equatorial Guinea | 0 | 2 | 962 | Equatorial Guinea Demographic and Health Survey 2011 | 2011 | ICF International, Ministry of Health and Social Welfare (Equatorial Guinea), Ministry of Planning, Economic Development and Public Investment (Equatorial Guinea). Equatorial Guinea Demographic and Health Survey 2011. Fairfax, United States of America: ICF International, 2012. | GHDx |
| **19539** | Eritrea | 0 | 6 | 2300 | Eritrea Demographic and Health Survey 2002 | 2002 | Macro International, Inc, National Statistics and Evaluation Office (Eritrea). Eritrea Demographic and Health Survey 2002. Fairfax, United States of America: ICF International. | GHDx |

| | | | | | | | | |
|---|---|---|---|---|---|---|---|---|
| **224938** | Eritrea | 0 | 6 | 2058 | Eritrea National EPI Coverage Survey 2013 | 2013 | Ministry of Health (Eritrea), United Nations Children's Fund (UNICEF), World Health Organization (WHO). Eritrea National EPI Coverage Survey 2013. | GHDx |
| **249999** | Eritrea | 0 | 6 | 1574 | Eritrea Population and Health Survey 2010 | 2010 | Kenya Medical Research Institute (KEMRI), National Statistics Office (Eritrea), The Fafo Research Foundation. Eritrea Population and Health Survey 2010. | GHDx |
| **19557** | Ethiopia | 524 | 0 | 5126 | Ethiopia Demographic and Health Survey 2005 | 2005 | Macro International, Inc, Population and Housing Census Commissions Office (PHCCO). Ethiopia Demographic and Health Survey 2005. Fairfax, United States of America: ICF International. | GHDx |
| **21301** | Ethiopia | 570 | 0 | 6425 | Ethiopia Demographic and Health Survey 2010-2011 | 2010-2011 | Central Statistical Agency (Ethiopia), ICF Macro, Ministry of Health (Ethiopia). Ethiopia Demographic and Health Survey 2010-2011. Fairfax, United States of America: ICF International. | GHDx |
| **218568** | Ethiopia | 611 | 0 | 3683 | Ethiopia Demographic and Health Survey 2016 | 2016 | Central Statistical Agency (Ethiopia), ICF International. Ethiopia Demographic and Health Survey 2016. Fairfax, United States of America: ICF International, 2017. | GHDx |
| **416582** | Ethiopia | 0 | 11 | 1025 | Ethiopia Special Demographic and Health Survey 2019 | 2019 | Central Statistical Agency (Ethiopia), Ethiopian Public Health Institute (EPHI), ICF International, Ministry of Health (Ethiopia). Ethiopia Special Demographic and Health Survey 2019. | GHDx |
| **76706** | Gabon | 328 | 0 | 3618 | Gabon Demographic and Health Survey 2012 | 2012 | General Directorate of Statistics (Gabon), ICF International, Ministry of Economy, Employment and Sustainable Development (Gabon), Ministry of Health (Gabon). Gabon Demographic and Health Survey 2012. Fairfax, United States of America: ICF International, 2013. | GHDx |
| **3935** | Gambia | 0 | 35 | 3927 | Gambia Multiple Indicator Cluster Survey 2005-2006 | 2005-2006 | Gambia Bureau of Statistics (GBOS), United Nations Children's Fund (UNICEF). Gambia Multiple Indicator Cluster Survey 2005-2006. New York, United States of America: United Nations Children's Fund (UNICEF). | GHDx |

| | | | | | | | | |
|---|---|---|---|---|---|---|---|---|
| **77384** | Gambia | 0 | 36 | 4235 | Gambia Demographic and Health Survey 2013 | 2013 | Gambia Bureau of Statistics (GBOS), ICF International, Ministry of Health and Social Welfare (The Gambia). Gambia Demographic and Health Survey 2013. Fairfax, United States of America: ICF International, 2015. | GHDx |
| **424884** | Gambia | 0 | 7 | 3602 | Gambia Multiple Indicator Cluster Survey 2018 | 2018 | Gambia Bureau of Statistics (GBOS), United Nations Children's Fund (UNICEF). Gambia Multiple Indicator Cluster Survey 2018. New York, United States of America: United Nations Children's Fund (UNICEF), 2019. | GHDx |
| **4694** | Ghana | 0 | 10 | 2059 | Ghana Multiple Indicator Cluster Survey 2006 | 2006 | Ministry of Health (MOH) (Ghana), Ghana Statistical Service and United Nations Children's Fund (UNICEF). Ghana Multiple Indicator Cluster Survey 2006. New York, United States: United Nations Children's Fund (UNICEF). | GHDx |
| **19627** | Ghana | 402 | 0 | 2116 | Ghana Demographic and Health Survey 2003 | 2003 | Ghana Statistical Service, Macro International, Inc. Ghana Demographic and Health Survey 2003. Fairfax, United States of America: ICF International. | GHDx |
| **21188** | Ghana | 391 | 0 | 1548 | Ghana Demographic and Health Survey 2008 | 2008 | Ghana Statistical Service, Macro International, Inc, Ministry of Health (Ghana). Ghana Demographic and Health Survey 2008. Fairfax, United States of America: ICF International. | GHDx |
| **56241** | Ghana | 5 | 0 | 258 | Ghana - Accra Multiple Indicator Cluster Survey 2010-2011 | 2010-2011 | Institute of Statistical, Social and Economic Research, University of Ghana, United Nations Children's Fund (UNICEF). Ghana - Accra Multiple Indicator Cluster Survey 2010-2011. New York, United States of America: United Nations Children's Fund (UNICEF), 2014. | GHDx |
| **63993** | Ghana | 730 | 0 | 4182 | Ghana Multiple Indicator Cluster Survey 2011 | 2011 | Centers for Disease Control and Prevention (CDC), Ghana Statistical Service, Government of Japan, ICF Macro, Ministry of Health (Ghana), Navrongo Health Research Centre, United Nations Children's Fund (UNICEF), United Nations Population Fund (UNFPA), United States Agency for International Development (USAID). Ghana Multiple Indicator Cluster Survey 2011. New York, United States of America: United Nations Children's Fund (UNICEF), 2013. | GHDx |

| | | | | | | | | |
|---|---|---|---|---|---|---|---|---|
| **157027** | Ghana | 422 | 0 | 3299 | Ghana Demographic and Health Survey 2014 | 2014 | Ghana Health Service, Ghana Statistical Service, ICF International. Ghana Demographic and Health Survey 2014. Fairfax, United States of America: ICF International, 2016. | GHDx |
| **160576** | Ghana | 0 | 4 | 5033 | Ghana District Multiple Indicator Cluster Survey 2007-2008 | 2007-2008 | Ghana Statistical Service, Ministry of Health (Ghana), United Nations Children's Fund (UNICEF). Ghana District Multiple Indicator Cluster Survey 2007-2008. | GHDx |
| **236205** | Ghana | 0 | 10 | 1365 | Ghana Socioeconomic Panel Survey 2009-2010 | 2009-2010 | Economic Growth Center, Yale University, Institute of Statistical, Social and Economic Research, University of Ghana. Ghana Socioeconomic Panel Survey 2009-2010. Washington DC, United States: World Bank. | GHDx |
| **437993** | Ghana | 0 | 10 | 3357 | Ghana Multiple Indicator Cluster Survey 2017-2018 | 2017-2018 | Centers for Disease Control and Prevention (CDC), Ghana Statistical Service, Government of Japan, ICF Macro, Ministry of Health (Ghana), Navrongo Health Research Centre, United Nations Children's Fund (UNICEF), United Nations Population Fund (UNFPA), United States Agency for International Development (USAID). Ghana Multiple Indicator Cluster Survey 2017-2018. New York, United States of America: United Nations Children's Fund (UNICEF), 2013. | GHDx |
| **4779** | Guatemala | 0 | 22 | 4150 | Guatemala Reproductive Health Survey 2008-2009 | 2008-2009 | Guatemala Ministry of Health and Social Assistance, University of Valle and Division of Reproductive Health-Centers for Disease Control and Prevention (CDC). Guatemala Reproductive Health Survey 2008-2009. Atlanta, United States: Centers for Disease Control and Prevention (CDC). | GHDx |
| **27563** | Guatemala | 326 | 0 | 1054 | Guatemala Reproductive Health Survey 2002 | 2002 | Guatemala Ministry of Health and Social Assistance, University of Valle, Division of Reproductive Health-Centers for Disease Control and Prevention (CDC). (2003) Guatemala Reproductive Health Survey 2002. Atlanta, United States: Centers for Disease Control and Prevention (CDC). | GHDx |

| | | | | | | | | |
|---|---|---|---|---|---|---|---|---|
| **19683** | Guinea | 291 | 0 | 3028 | Guinea Demographic and Health Survey 2005 | 2005 | Macro International, Inc, National Statistics Directorate (Guinea). Guinea Demographic and Health Survey 2005. Fairfax, United States of America: ICF International. | GHDx |
| **69761** | Guinea | 300 | 0 | 3738 | Guinea Demographic and Health Survey 2012 | 2012 | ICF Macro, Ministry of Health and Public Hygiene (Guinea), National Institute of Statistics (Guinea). Guinea Demographic and Health Survey 2012. Fairfax, United States of America: ICF International. | GHDx |
| **303458** | Guinea | 0 | 8 | 4324 | Guinea Multiple Indicator Cluster Survey 2016 | 2016 | National Institute of Public Health (NPHI) (Guinea), National Institute of Statistics (Guinea), National Malaria Control Program (Guinea), United Nations Children's Fund (UNICEF). Guinea Multiple Indicator Cluster Survey 2016. New York, United States of America: United Nations Children's Fund (UNICEF), 2018. | GHDx |
| **396957** | Guinea | 397 | 0 | 2626 | Guinea Demographic and Health Survey 2018 | 2018 | Ministry of Health (Guinea), Ministry of Planning and Economic Development (Guinea), National Institute of Statistics (Guinea). Guinea Demographic and Health Survey 2018. Fairfax, United States of America: ICF International, 2019. | GHDx |
| **4818** | Guinea-Bissau | 0 | 9 | 3426 | Guinea-Bissau Multiple Indicator Cluster Survey 2006 | 2006 | United Nations Children's Fund (UNICEF), Government of Guinea-Bissau. Guinea-Bissau Multiple Indicator Cluster Survey 2006. New York, United States: United Nations Children's Fund (UNICEF). | GHDx |
| **174049** | Guinea-Bissau | 0 | 9 | 3003 | Guinea-Bissau Multiple Indicator Cluster Survey 2014 | 2014 | National Statistics Institute (Guinea-Bissau), United Nations Children's Fund (UNICEF). Guinea-Bissau Multiple Indicator Cluster Survey 2014. New York, United States of America: United Nations Children's Fund (UNICEF), 2016. | GHDx |
| **4926** | Guyana | 0 | 10 | 1849 | Guyana Multiple Indicator Cluster Survey 2006-2007 | 2006-2007 | United Nations Children's Fund (UNICEF), Bureau of Statistics (Guyana). Guyana Multiple Indicator Cluster Survey 2006. New York, United States: United Nations Children's Fund (UNICEF). | GHDx |
| **21348** | Guyana | 294 | 0 | 1348 | Guyana Demographic | 2009 | Bureau of Statistics (Guyana), ICF Macro, Ministry of Health (Guyana). Guyana Demographic and Health Survey | GHDx |

| | | | | | | | | |
|---|---|---|---|---|---|---|---|---|
| | | | | | and Health Survey 2009 | | 2009. Fairfax, United States of America: ICF International, 2011. | |
| **200598** | Guyana | 244 | 0 | 1326 | Guyana Multiple Indicator Cluster Survey 2014 | 2014 | Bureau of Statistics (Guyana), Ministry of Health (Guyana), United Nations Children's Fund (UNICEF). Guyana Multiple Indicator Cluster Survey 2014. New York, United States of America: United Nations Children's Fund (UNICEF), 2016. | GHDx |
| **19720** | Haiti | 332 | 0 | 3146 | Haiti Demographic and Health Survey 2005-2006 | 2005-2006 | Haitian Institute of Childhood (IHE), Haitian Institute of Statistics and Informatics, Macro International, Inc. Haiti Demographic and Health Survey 2005-2006. Fairfax, United States of America: ICF International. | GHDx |
| **26680** | Haiti | 0 | 9 | 1481 | Haiti Global Fund Household Survey 2008 | 2008 | Global Fund to Fight Aids Tuberculosis and Malaria (GFATM). Haiti Global Fund Household Survey 2008. | GHDx |
| **65118** | Haiti | 437 | 0 | 3904 | Haiti Demographic and Health Survey 2012 | 2012 | Centers for Disease Control and Prevention (CDC), Haitian Institute of Childhood (IHE), Haitian Institute of Statistics and Informatics, Macro International, Inc. Haiti Demographic and Health Survey 2012. Fairfax, United States of America: ICF International. | GHDx |
| **218574** | Haiti | 441 | 0 | 2421 | Haiti Demographic and Health Survey 2016-2017 | 2016-2017 | Haitian Institute of Childhood (IHE), Haitian Institute of Statistics and Informatics, ICF International, Ministry of Public Health and Population (Haiti). Haiti Demographic and Health Survey 2016-2017. Fairfax, United States of America: ICF International. | GHDx |
| **19728** | Honduras | 0 | 16 | 6277 | Honduras Demographic and Health Survey 2005-2006 | 2005-2006 | Macro International, Inc, National Institute of Statistics (Honduras), Secretary of Health (Honduras). Honduras Demographic and Health Survey 2005-2006. Fairfax, United States of America: ICF International. | GHDx |
| **95440** | Honduras | 1111 | 0 | 5974 | Honduras Demographic and Health Survey 2011-2012 | 2011-2012 | ICF Macro, National Institute of Statistics (Honduras). Honduras Demographic and Health Survey 2011-2012. Fairfax, United States of America: ICF International. | GHDx |

| | | | | | | | | |
|---|---|---|---|---|---|---|---|---|
| **19963** | India | 0 | 29 | 32394 | India Demographic and Health Survey 2005-2006 | 2005-2006 | International Institute for Population Sciences (India), Macro International, Inc. India Demographic and Health Survey 2005-2006. Fairfax, United States of America: ICF International. | GHDx |
| **157050** | India | 27634 | 18 | 162073 | India Demographic and Health Survey 2015-2016 | 2015-2016 | ICF International, International Institute for Population Sciences (India), Ministry of Health and Family Welfare (India). India Demographic and Health Survey 2015-2016. Fairfax, United States of America: ICF International, 2018. | GHDx |
| **261875** | India | 1 | 0 | 129 | India - Vellore Malnutrition and Enteric Disease Study 2009-2014 | 2009-2014 | Fogarty International Center, National Institutes of Health (NIH), Foundation for the National Institutes of Health (FNIH). India - Vellore Malnutrition and Enteric Disease Study 2009-2014. | GHDx |
| **6464** | Indonesia | 0 | 15 | 2183 | Indonesia Family Life Survey 2007-2008 | 2007-2008 | Center for Population and Policy Studies, Gadjah Mada University (Indonesia), RAND Corporation, SurveyMETER. Indonesia Family Life Survey 2007-2008. Santa Monica, United States of America: RAND Corporation. | GHDx |
| **20011** | Indonesia | 1284 | 0 | 6055 | Indonesia Demographic and Health Survey 2002-2003 | 2002-2003 | Macro International, Inc, Ministry of Health (Indonesia), National Family Planning Coordinating Board (Indonesia), Statistics Indonesia. Indonesia Demographic and Health Survey 2002-2003. Fairfax, United States of America: ICF International. | GHDx |
| **20021** | Indonesia | 0 | 33 | 10349 | Indonesia Demographic and Health Survey 2007 | 2007 | Macro International, Inc, Ministry of Health (Indonesia), National Family Planning Coordinating Board (Indonesia), Statistics Indonesia. Indonesia Demographic and Health Survey 2007. Fairfax, United States of America: ICF International. | GHDx |
| **76705** | Indonesia | 0 | 33 | 10226 | Indonesia Demographic and Health Survey 2012 | 2012 | ICF International, Ministry of Health (Indonesia), National Population and Family Planning Board (Indonesia), Statistics Indonesia. Indonesia Demographic and Health Survey 2012. Fairfax, United States of America: ICF International. | GHDx |

| | | | | | | | | |
|---|---|---|---|---|---|---|---|---|
| **104042** | Indonesia | 0 | 3 | 832 | Indonesia - West Papua Multiple Indicator Cluster Survey 2011 | 2011 | Ministry of Home Affairs (Indonesia), National Development Planning Agency (BAPPENAS) (Indonesia), Statistics Indonesia, United Nations Children's Fund (UNICEF). Indonesia - West Papua Multiple Indicator Cluster Survey 2011. New York, United States of America: United Nations Children's Fund (UNICEF), 2013. | GHDx |
| **104043** | Indonesia | 0 | 3 | 885 | Indonesia - Papua Multiple Indicator Cluster Survey 2011 | 2011 | Ministry of Home Affairs (Indonesia), National Development Planning Agency (BAPPENAS) (Indonesia), Statistics Indonesia, United Nations Children's Fund (UNICEF). Indonesia - Papua Multiple Indicator Cluster Survey 2011. New York, United States of America: United Nations Children's Fund (UNICEF), 2013. | GHDx |
| **264956** | Indonesia | 0 | 932 | 3107 | Indonesia Family Life Survey 2014-2015 | 2014-2015 | RAND Corporation, SurveyMETER. Indonesia Family Life Survey 2014-2015. Santa Monica, United States of America: RAND Corporation, 2016. | GHDx |
| **286781** | Indonesia | 0 | 33 | 6794 | Indonesia Demographic and Health Survey 2017 | 2017 | Ministry of Health (Indonesia), National Population and Family Planning Board (Indonesia), Statistics Indonesia. Indonesia Demographic and Health Survey 2017. Fairfax, United States of America: ICF International, 2019. | GHDx |
| **395694** | Indonesia | 0 | 34 | 17816 | Indonesia National Socioeconomic Survey 2017 | 2017 | Central Bureau of Statistics (Indonesia). Indonesia National Socioeconomic Survey 2017. Jakarta, Indonesia: Central Bureau of Statistics (Indonesia), 2018. | GHDx |
| **81416** | Iran | 0 | 30 | 5681 | Iran Multiple Indicator Demographic and Health Survey 2010 | 2010 | Ministry of Health and Medical Education (Iran), Statistical Centre of Iran. Iran Multiple Indicator Demographic and Health Survey 2010. | GHDx |
| **7028** | Iraq | 0 | 18 | 9666 | Iraq Multiple Indicator Cluster Survey 2006 | 2006 | United Nations Children's Fund (UNICEF), Central Organization for Statistics and Information Technology (Iraq), Kurdistan Regional Statistics Office. Iraq Multiple Indicator Cluster Survey 2006. New York, United States: United Nations Children's Fund (UNICEF). | GHDx |
| **76707** | Iraq | 0 | 18 | 7485 | Iraq Multiple Indicator | 2011 | Central Organization for Statistics and Information Technology (Iraq), Kurdistan Regional Statistics Office, | GHDx |

| | | | | | | | | |
|---|---|---|---|---|---|---|---|---|
| | | | | | Cluster Survey 2011 | | Ministry of Health (Iraq), United Nations Children's Fund (UNICEF). Iraq Multiple Indicator Cluster Survey 2011. New York, United States of America: United Nations Children's Fund (UNICEF), 2013. | |
| **385708** | Iraq | 0 | 190 | 6098 | Iraq Multiple Indicator Cluster Survey 2018 | 2018 | Central Statistical Organization (Iraq), United Nations Children's Fund (UNICEF). Iraq Multiple Indicator Cluster Survey 2018. New York, United States of America: United Nations Children's Fund (UNICEF), 2019. | GHDx |
| **7149** | Jamaica | 0 | 14 | 850 | Jamaica Multiple Indicator Cluster Survey 2005 | 2005 | Statistical Institute of Jamaica (STATIN) and United Nations Children's Fund (UNICEF). Jamaica Multiple Indicator Cluster Survey 2005. New York, United States: United Nations Children's Fund (UNICEF). | GHDx |
| **141336** | Jamaica | 0 | 14 | 979 | Jamaica Multiple Indicator Cluster Survey 2011 | 2011 | Statistical Institute of Jamaica, United Nations Children's Fund (UNICEF). Jamaica Multiple Indicator Cluster Survey 2011. New York, United States of America: United Nations Children's Fund (UNICEF), 2015. | GHDx |
| **20073** | Jordan | 489 | 0 | 2381 | Jordan Demographic and Health Survey 2002 | 2002 | Department of Statistics (Jordan), Macro International, Inc. Jordan Demographic and Health Survey 2002. Fairfax, United States of America: ICF International. | GHDx |
| **20083** | Jordan | 916 | 0 | 6058 | Jordan Demographic and Health Survey 2007 | 2007 | Department of Statistics (Jordan), Macro International, Inc. Jordan Demographic and Health Survey 2007. Fairfax, United States of America: ICF International. | GHDx |
| **77517** | Jordan | 802 | 0 | 6137 | Jordan Demographic and Health Survey 2012 | 2012 | Department of Statistics (Jordan), ICF International. Jordan Demographic and Health Survey 2012. Fairfax, United States of America: ICF International. | GHDx |
| **356955** | Jordan | 929 | 0 | 4006 | Jordan Demographic and Health Survey 2017-2018 | 2017-2018 | Department of Statistics (Jordan), ICF International. Jordan Demographic and Health Survey 2017-2018. Fairfax, United States of America: ICF International, 2019. | GHDx |

| | | | | | | | | |
|---|---|---|---|---|---|---|---|---|
| **7375** | Kenya | 1134 | 0 | 3372 | Kenya Integrated Household Budget Survey 2005-2006 | 2005-2006 | Central Bureau of Statistics (Kenya), UK Department for International Development (DFID), United States Agency for International Development (USAID), European Union (EU), Danish International Development Agency (DANIDA), World Bank (WB), United Nations Development Programme (UNDP). Kenya Integrated Household Budget Survey 2005-2006. Nairobi, Kenya: Central Bureau of Statistics (Kenya). | GHDx |
| **7401** | Kenya | 590 | 0 | 8563 | Kenya - Eastern Province Multiple Indicator Cluster Survey 2008 | 2008 | Kenya National Bureau of Statistics, United Nations Children's Fund (UNICEF). Kenya - Eastern Province Multiple Indicator Cluster Survey 2008. Nairobi, Kenya: Kenya National Bureau of Statistics. | GHDx |
| **21365** | Kenya | 396 | 0 | 3328 | Kenya Demographic and Health Survey 2008-2009 | 2008-2009 | ICF Macro, Kenya Medical Research Institute (KEMRI), Kenya National Bureau of Statistics, Ministry of Public Health and Sanitation (Kenya), National AIDS and STI Control Programme (NASCOP) (Kenya), National Aids Control Council (NACC), National Coordinating Agency for Population and Development (Kenya). Kenya Demographic and Health Survey 2008-2009. Fairfax, United States of America: ICF International. | GHDx |
| **56420** | Kenya | 0 | 1 | 277 | Kenya - Coast Multiple Indicator Cluster Survey 2009 | 2009 | Kenya National Bureau of Statistics, United Nations Children's Fund (UNICEF). Kenya - Coast Multiple Indicator Cluster Survey 2009. New York, United States of America: United Nations Children's Fund (UNICEF), 2014. | GHDx |
| **135416** | Kenya | 289 | 0 | 2894 | Kenya - Nyanza Province Multiple Indicator Cluster Survey 2011 | 2011 | Kenya National Bureau of Statistics, United Nations Children's Fund (UNICEF). Kenya - Nyanza Province Multiple Indicator Cluster Survey 2011. Nairobi, Kenya: Kenya National Bureau of Statistics. | GHDx |

| | | | | | | | | |
|---|---|---|---|---|---|---|---|---|
| **157057** | Kenya | 1581 | 0 | 12164 | Kenya Demographic and Health Survey 2014 | 2014 | ICF International, Kenya Medical Research Institute (KEMRI), Kenya National Bureau of Statistics, Ministry of Health (Kenya), National AIDS Control Council (Kenya), National Council for Population and Development (Kenya). Kenya Demographic and Health Survey 2014. Fairfax, United States of America: ICF International. | GHDx |
| **203654** | Kenya | 40 | 0 | 520 | Kenya - Bungoma County Multiple Indicator Survey 2013-2014 | 2013-2014 | Kenya National Bureau of Statistics, Population Studies and Research Institute, University of Nairobi (Kenya), United Nations Children's Fund (UNICEF). Kenya - Bungoma County Multiple Indicator Survey 2013-2014. New York, United States of America: United Nations Children's Fund (UNICEF), 2015. | GHDx |
| **203663** | Kenya | 48 | 0 | 474 | Kenya - Kakamega County Multiple Indicator Survey 2013-2014 | 2013-2014 | Kenya National Bureau of Statistics, Population Studies and Research Institute, University of Nairobi (Kenya), United Nations Children's Fund (UNICEF). Kenya - Kakamega County Multiple Indicator Survey 2013-2014. New York, United States of America: United Nations Children's Fund (UNICEF), 2015. | GHDx |
| **203664** | Kenya | 49 | 0 | 596 | Kenya - Turkana County Multiple Indicator Survey 2013-2014 | 2013-2014 | Kenya National Bureau of Statistics, Population Studies and Research Institute, University of Nairobi (Kenya), United Nations Children's Fund (UNICEF). Kenya - Turkana County Multiple Indicator Survey 2013-2014. New York, United States of America: United Nations Children's Fund (UNICEF), 2015. | GHDx |
| **7540** | Kyrgyzstan | 182 | 2 | 1677 | Kyrgyzstan Multiple Indicator Cluster Survey 2005-2006 | 2005-2006 | United Nations Children's Fund (UNICEF), National Statistical Committee of the Kyrgyz Republic. Kyrgyzstan Multiple Indicator Cluster Survey 2005-2006. New York, United States: United Nations Children's Fund (UNICEF). | GHDx |
| **77518** | Kyrgyzstan | 312 | 0 | 2456 | Kyrgyzstan Demographic and Health Survey 2012 | 2012 | ICF International, Ministry of Health (Kyrgyzstan), National Statistical Committee of the Kyrgyz Republic. Kyrgyzstan Demographic and Health Survey 2012. Fairfax, United States of America: ICF International. | GHDx |

| | | | | | | | | |
|---|---|---|---|---|---|---|---|---|
| **408226** | Kyrgyzstan | 188 | 3 | 1273 | Kyrgyzstan Multiple Indicator Cluster Survey 2018 | 2018 | National Statistical Committee of the Kyrgyz Republic, United Nations Children's Fund (UNICEF). Kyrgyzstan Multiple Indicator Cluster Survey 2018. New York, United States of America: United Nations Children's Fund (UNICEF), 2019. | GHDx |
| **7629** | Laos | 298 | 0 | 2724 | Laos Multiple Indicator Cluster Survey 2006 | 2006 | United Nations Children's Fund (UNICEF), Department of Statistics (Laos), Ministry of Health (Laos). Laos Multiple Indicator Cluster Survey 2006. New York, United States: United Nations Children's Fund (UNICEF). | GHDx |
| **103973** | Laos | 0 | 17 | 7503 | Laos Multiple Indicator Cluster Survey 2011-2012 | 2011-2012 | Ministry of Education and Sports (Laos), Ministry of Health (Laos), Ministry of Planning and Investment (Laos). Laos Multiple Indicator Cluster Survey 2011-2012. New York, United States of America: United Nations Children's Fund (UNICEF), 2013. | GHDx |
| **375362** | Laos | 0 | 18 | 5047 | Laos Multiple Indicator Cluster Survey 2017 | 2017 | Lao Statistics Bureau, Ministry of Education and Sports (Laos), Ministry of Health (Laos), United Nations Children's Fund (UNICEF). Laos Multiple Indicator Cluster Survey 2017. New York, United States of America: United Nations Children's Fund (UNICEF), 2018. | GHDx |
| **20167** | Lesotho | 371 | 0 | 1864 | Lesotho Demographic and Health Survey 2004-2005 | 2004-2005 | Bureau of Statistics (Lesotho), Macro International, Inc, Ministry of Health and Social Welfare (Lesotho). Lesotho Demographic and Health Survey 2004-2005. Fairfax, United States of America: ICF International. | GHDx |
| **21382** | Lesotho | 392 | 0 | 2077 | Lesotho Demographic and Health Survey 2009-2010 | 2009-2010 | ICF Macro, Ministry of Health and Social Welfare (Lesotho). Lesotho Demographic and Health Survey 2009-2010. Fairfax, United States of America: ICF International. | GHDx |
| **157058** | Lesotho | 390 | 0 | 1713 | Lesotho Demographic and Health Survey 2014 | 2014 | ICF International, Ministry of Health and Social Welfare (Lesotho). Lesotho Demographic and Health Survey 2014. Fairfax, United States of America: ICF International. | GHDx |
| **20191** | Liberia | 291 | 0 | 3102 | Liberia Demographic and Health | 2006-2007 | Liberia Institute for Statistics and Geo-information Services (LISGIS), Macro International, Inc. Liberia | GHDx |

| | | | | | | | | |
|---|---|---|---|---|---|---|---|---|
| | | | | | Survey 2006-2007 | | Demographic and Health Survey 2006-2007. Fairfax, United States of America: ICF International. | |
| **77385** | Liberia | 322 | 0 | 4107 | Liberia Demographic and Health Survey 2013 | 2013 | ICF International, Liberia Institute for Statistics and Geo-information Services (LISGIS), National AIDS and STI Control Program (NACP), Ministry of Health and Social Welfare (Liberia). Liberia Demographic and Health Survey 2013. Fairfax, United States of America: ICF International. | GHDx |
| **286768** | Liberia | 149 | 0 | 1101 | Liberia Malaria Indicator Survey 2016 | 2016 | ICF International, Liberia Institute for Statistics and Geo-information Services (LISGIS), National Malaria Control Program (Liberia). Liberia Malaria Indicator Survey 2016. Fairfax, United States of America: ICF International, 2017. | GHDx |
| **20223** | Madagascar | 0 | 6 | 3022 | Madagascar Demographic and Health Survey 2003-2004 | 2003-2004 | Macro International, Inc, National Institute of Statistics (Madagascar). Madagascar Demographic and Health Survey 2003-2004. Fairfax, United States of America: ICF International. | GHDx |
| **21409** | Madagascar | 585 | 0 | 6672 | Madagascar Demographic and Health Survey 2008-2009 | 2008-2009 | ICF Macro, National Institute of Statistics (Madagascar). Madagascar Demographic and Health Survey 2008-2009. Fairfax, United States of America: ICF International. | GHDx |
| **125594** | Madagascar | 127 | 0 | 1705 | Madagascar - South Multiple Indicator Cluster Survey 2012 | 2012 | National Institute of Statistics (Madagascar), United Nations Children's Fund (UNICEF). Madagascar - South Multiple Indicator Cluster Survey 2012. New York, United States of America: United Nations Children's Fund (UNICEF), 2015. | GHDx |
| **399853** | Madagascar | 766 | 0 | 5012 | Madagascar Multiple Indicator Cluster Survey 2018 | 2018 | National Institute of Statistics (Madagascar), United Nations Children's Fund (UNICEF). Madagascar Multiple Indicator Cluster Survey 2018. 2019. | GHDx |
| **7919** | Malawi | 0 | 26 | 14466 | Malawi Multiple Indicator Cluster Survey 2006 | 2006 | United Nations Children's Fund (UNICEF), National Statistics Office (Malawi). Malawi Multiple Indicator Cluster Survey 2006. New York, United States: United Nations Children's Fund (UNICEF). | GHDx |

| | | | | | | | | |
|---|---|---|---|---|---|---|---|---|
| **20263** | Malawi | 520 | 0 | 5657 | Malawi Demographic and Health Survey 2004-2005 | 2004-2005 | Macro International, Inc, National Statistical Office of Malawi. Malawi Demographic and Health Survey 2004-2005. Fairfax, United States of America: ICF International. | GHDx |
| **21393** | Malawi | 826 | 0 | 10826 | Malawi Demographic and Health Survey 2010 | 2010 | ICF Macro, National Statistical Office of Malawi. Malawi Demographic and Health Survey 2010. Fairfax, United States of America: ICF International. | GHDx |
| **161662** | Malawi | 0 | 31 | 7573 | Malawi Multiple Indicator Cluster Survey 2013-2014 | 2013-2014 | National Statistical Office of Malawi, United Nations Children's Fund (UNICEF). Malawi Multiple Indicator Cluster Survey 2013-2014. New York, United States of America: United Nations Children's Fund (UNICEF), 2015. | GHDx |
| **218581** | Malawi | 849 | 0 | 6489 | Malawi Demographic and Health Survey 2015-2016 | 2015-2016 | Emory University and Centers for Disease Control & Prevention Collaboration, ICF International, Ministry of Health (Malawi), National Statistical Office of Malawi. Malawi Demographic and Health Survey 2015-2016. Fairfax, United States of America: ICF International, 2017. | GHDx |
| **327852** | Malawi | 0 | 32 | 4519 | Malawi Integrated Household Survey 2016-2017 | 2016-2017 | National Statistical Office. Malawi - Fourth Integrated Household Survey 2016-2017, Ref. MWI_2016_IHS-IV_v02_M. Dataset downloaded from [http://microdata.worldbank.org/index.php/catalog/2936] on [December 18, 2017]. | GHDx |
| **284410** | Malaysia | 0 | 16 | 10878 | Malaysia National Health and Morbidity Survey 2016 | 2016 | Institute for Public Health, Ministry of Health (Malaysia), National Institutes of Health (NIH). Malaysia National Health and Morbidity Survey 2016. | GHDx |
| **20274** | Mali | 405 | 0 | 7116 | Mali Demographic and Health Survey 2006 | 2006 | Macro International, Inc, Ministry of Health (Mali), National Directorate of Statistics and Informatics (DNSI) (Mali). Mali Demographic and Health Survey 2006. Fairfax, United States of America: ICF International. | GHDx |
| **20315** | Mali | 399 | 0 | 2170 | Mali Demographic | 2001 | Macro International, Inc, National Directorate of Statistics and Informatics (DNSI) (Mali), Planning and Statistics Unit, Ministry of Health (Mali). Mali Demographic and | GHDx |

| | | | | | | | | |
|---|---|---|---|---|---|---|---|---|
| | | | | | and Health Survey 2001 | | Health Survey 2001. Fairfax, United States of America: ICF International. | |
| **77388** | Mali | 413 | 0 | 5605 | Mali Demographic and Health Survey 2012-2013 | 2012-2013 | ICF International, INFO-STAT (Mali), Ministry of Health (Mali), National Institute of Statistics (INSTAT) (Mali), Planning and Statistics Unit, Ministry of Health (Mali). Mali Demographic and Health Survey 2012-2013. Fairfax, United States of America: ICF International, 2014. | GHDx |
| **248224** | Mali | 0 | 8 | 6261 | Mali Multiple Indicator Cluster Survey 2015 | 2015 | Ministry of Health (Mali), Ministry of Planning (Mali), National Institute of Statistics (INSTAT) (Mali), United Nations Children's Fund (UNICEF). Mali Multiple Indicator Cluster Survey 2015. New York, United States of America: United Nations Children's Fund (UNICEF), 2017. | GHDx |
| **260407** | Mali | 0 | 48 | 2800 | Mali Agricultural Integrated Economic Survey 2014-2015 | 2014-2015 | Ministry of Rural Development (Mali), National Institute of Statistics (INSTAT) (Mali), World Bank. Mali Agricultural Integrated Economic Survey 2014-2015. Washington DC, United States of America: World Bank. | GHDx |
| **398033** | Mali | 328 | 0 | 3479 | Mali Demographic and Health Survey 2018 | 2018 | ICF International, National Institute of Statistics (INSTAT) (Mali). Mali Demographic and Health Survey 2018. Fairfax, United States of America: ICF International, 2019. | GHDx |
| **8115** | Mauritania | 0 | 196 | 5660 | Mauritania Multiple Indicator Cluster Survey 2007 | 2007 | National Office of Statistics (Mauritania), United Nations Children's Fund (UNICEF). Mauritania Multiple Indicator Cluster Survey 2007. New York, United States of America: United Nations Children's Fund (UNICEF). | GHDx |
| **152783** | Mauritania | 0 | 194 | 5357 | Mauritania Multiple Indicator Cluster Survey 2011 | 2011 | National Office of Statistics (Mauritania), United Nations Children's Fund (UNICEF). Mauritania Multiple Indicator Cluster Survey 2011. New York, United States of America: United Nations Children's Fund (UNICEF), 2015. | GHDx |
| **267343** | Mauritania | 0 | 13 | 4606 | Mauritania Multiple Indicator | 2015 | National Office of Statistics (Mauritania), United Nations Children's Fund (UNICEF). Mauritania Multiple Indicator | GHDx |

| | | | | | | | | |
|---|---|---|---|---|---|---|---|---|
| | | | | | Cluster Survey 2015 | | Cluster Survey 2015. New York, United States of America: United Nations Children's Fund (UNICEF), 2018. | |
| **8442** | Mexico | 0 | 126 | 3889 | Mexico Family Life Survey 2002 | 2002 | Center for Research and Teaching in Economics (CIDE) (Mexico), Ibero-American University, National Institute of Perinatology (Mexico), National Institute of Statistics and Geography (INEGI) (Mexico). Mexico Family Life Survey 2002. | GHDx |
| **8618** | Mexico | 0 | 578 | 8852 | Mexico National Survey of Health and Nutrition 2005-2006 | 2005-2006 | National Institute of Public Health (Mexico). Mexico National Survey of Health and Nutrition 2005-2006. Cuernavaca, Mexico: National Institute of Public Health (Mexico). | GHDx |
| **8684** | Mexico | 0 | 1 | 10 | Mexico National Performance Evaluation Survey 2002-2003 | 2002-2003 | National Institute of Public Health (Mexico), Secretariat of Health (Mexico), World Health Organization (WHO). Mexico National Performance Evaluation Survey 2002-2003. | GHDx |
| **81748** | Mexico | 0 | 689 | 5067 | Mexico National Survey of Health and Nutrition 2011-2012 | 2011-2012 | National Institute of Public Health (Mexico). Mexico National Survey of Health and Nutrition 2011-2012. Cuernavaca, Mexico: National Institute of Public Health (Mexico). | GHDx |
| **264590** | Mexico | 0 | 5 | 3157 | Mexico Multiple Indicator Cluster Survey 2015 | 2015 | National Institute of Public Health (Mexico), United Nations Children's Fund (UNICEF). Mexico Multiple Indicator Cluster Survey 2015. New York, United States: United Nations Children's Fund (UNICEF), 2017. | GHDx |
| **20339** | Moldova | 342 | 0 | 845 | Moldova Demographic and Health Survey 2005 | 2005 | Macro International, Inc, National Scientific and Applied Center for Preventive Medicine (Moldova). Moldova Demographic and Health Survey 2005. Fairfax, United States of America: ICF International. | GHDx |
| **140200** | Moldova | 0 | 4 | 1042 | Moldova Multiple | 2012 | Ministry of Health (Moldova), National Bureau of Statistics (Moldova), United Nations Children's Fund | GHDx |

| | | | | | | | | |
|---|---|---|---|---|---|---|---|---|
| | | | | | Indicator Cluster Survey 2012 | | (UNICEF). Moldova Multiple Indicator Cluster Survey 2012. New York, United States of America: United Nations Children's Fund (UNICEF), 2015. | |
| **8777** | Mongolia | 0 | 22 | 2089 | Mongolia Multiple Indicator Cluster Survey 2005 | 2005 | National Statistical Office of Mongolia, United Nations Children's Fund (UNICEF). Mongolia Multiple Indicator Cluster Survey 2005. New York, United States of America: United Nations Children's Fund (UNICEF). | GHDx |
| **76704** | Mongolia | 0 | 217 | 2448 | Mongolia Multiple Indicator Cluster Survey 2010 | 2010 | National Statistical Office of Mongolia, United Nations Children's Fund (UNICEF). Mongolia Multiple Indicator Cluster Survey 2010. New York, United States of America: United Nations Children's Fund (UNICEF), 2013. | GHDx |
| **189045** | Mongolia | 0 | 23 | 503 | Mongolia - Khuvsgul Multiple Indicator Cluster Survey 2012 | 2012 | National Statistical Office of Mongolia, Statistics Department of Khuvsgul Aimag (Mongolia), United Nations Children's Fund (UNICEF). Mongolia - Khuvsgul Multiple Indicator Cluster Survey 2012. New York, United States of America: United Nations Children's Fund (UNICEF), 2015. | GHDx |
| **189048** | Mongolia | 0 | 1 | 259 | Mongolia - Nalaikh District Multiple Cluster Indicator Survey 2012 | 2012 | National Statistical Office of Mongolia, Statistics Department, Governor’s Office of the Nalaikh (Mongolia), United Nations Children's Fund (UNICEF). Mongolia - Nalaikh District Multiple Cluster Indicator Survey 2012. New York, United States of America: United Nations Children's Fund (UNICEF), 2017. | GHDx |
| **335994** | Mongolia | 0 | 80 | 432 | Mongolia - Khuvsgul Multiple Indicator Cluster Survey 2016 | 2016 | National Statistical Office of Mongolia, United Nations Children's Fund (UNICEF). Mongolia - Khuvsgul Multiple Indicator Cluster Survey 2016. New York, United States of America: United Nations Children's Fund (UNICEF), 2018. | GHDx |
| **336042** | Mongolia | 0 | 7 | 144 | Mongolia - Nalaikh District Multiple | 2016 | National Statistical Office of Mongolia, United Nations Children's Fund (UNICEF). Mongolia - Nalaikh District Multiple Indicator Cluster Survey 2016. New York, United | GHDx |

| | | | | | | | | |
|---|---|---|---|---|---|---|---|---|
| | | | | | Indicator Cluster Survey 2016 | | States of America: United Nations Children's Fund (UNICEF), 2018. | |
| **427952** | Mongolia | 0 | 447 | 2194 | Mongolia Multiple Indicator Cluster Survey 2018 | 2018 | Government of Mongolia, National Statistical Office of Mongolia, United Nations Children's Fund (UNICEF), United Nations Population Fund (UNFPA). Mongolia Multiple Indicator Cluster Survey 2018. New York, United States of America: United Nations Children's Fund (UNICEF), 2019. | GHDx |
| **20361** | Morocco | 476 | 0 | 3548 | Morocco Demographic and Health Survey 2003-2004 | 2003-2004 | League of Arab States, Macro International, Inc, Ministry of Health (Morocco). Morocco Demographic and Health Survey 2003-2004. Fairfax, United States of America: ICF International. | GHDx |
| **126909** | Morocco | 0 | 59 | 1691 | Morocco National Survey on Population and Family Health 2010-2011 | 2010-2011 | Ministry of Health (Morocco), Pan Arab Project for Family Health (PAPFAM), United Nations Children's Fund (UNICEF), United Nations Population Fund (UNFPA), World Health Organization (WHO). Morocco National Survey on Population and Family Health 2010-2011. | GHDx |
| **20394** | Mozambique | 0 | 11 | 6833 | Mozambique Demographic and Health Survey 2003-2004 | 2003-2004 | Macro International, Inc, National Institute of Statistics (INE) (Mozambique). Mozambique Demographic and Health Survey 2003-2004. Fairfax, United States of America: ICF International. | GHDx |
| **27031** | Mozambique | 67 | 593 | 8027 | Mozambique Multiple Indicator Cluster Survey 2008-2009 | 2008-2009 | United Nations Children's Fund (UNICEF), National Statistics Institute (Mozambique). Mozambique Multiple Indicator Cluster Survey 2008-2009. New York, United States: United Nations Children's Fund (UNICEF). | GHDx |
| **55975** | Mozambique | 609 | 0 | 7803 | Mozambique Demographic and Health Survey 2011 | 2011 | ICF Macro, Manhica Health Research Center (CISM), Ministry of Health (Mozambique), National Institute of Statistics (INE) (Mozambique). Mozambique Demographic and Health Survey 2011. Fairfax, United States of America: ICF International. | GHDx |

| | | | | | | | | |
|---|---|---|---|---|---|---|---|---|
| **157060** | Mozambique | 305 | 0 | 3648 | Mozambique AIDS Indicator Survey 2015 | 2015 | Centers for Disease Control and Prevention (CDC), ICF International, Ministry of Health (Mozambique), National Institute of Health (Mozambique), National Institute of Statistics (INE) (Mozambique). Mozambique AIDS Indicator Survey 2015. Fairfax, United States of America: ICF International, 2018. | GHDx |
| **90696** | Myanmar | 0 | 17 | 10601 | Myanmar Multiple Indicator Cluster Survey 2009-2010 | 2009-2010 | Ministry of Health (Myanmar), Ministry of National Planning and Economic Development (Myanmar), United Nations Children's Fund (UNICEF). Myanmar Multiple Indicator Cluster Survey 2009-2010. | GHDx |
| **141910** | Myanmar | 0 | 17 | 9368 | Myanmar Multiple Indicator Cluster Survey 2003 | 2003 | Ministry of Health (Myanmar), United Nations Children's Fund (UNICEF). Myanmar Multiple Indicator Cluster Survey 2003. | GHDx |
| **157061** | Myanmar | 434 | 0 | 3374 | Myanmar Demographic and Health Survey 2015-2016 | 2015-2016 | ICF International, Ministry of Health and Sports (Myanmar). Myanmar Demographic and Health Survey 2015-2016. Fairfax, United States of America: ICF International, 2017. | GHDx |
| **20428** | Namibia | 483 | 0 | 4520 | Namibia Demographic and Health Survey 2006-2007 | 2006-2007 | Macro International, Inc, Ministry of Health and Social Services (Namibia). Namibia Demographic and Health Survey 2006-2007. Fairfax, United States of America: ICF International. | GHDx |
| **150382** | Namibia | 530 | 0 | 4331 | Namibia Demographic and Health Survey 2013 | 2013 | ICF International, Ministry of Health and Social Services (Namibia), Namibia Institute of Pathology, Namibia Statistics Agency. Namibia Demographic and Health Survey 2013. Fairfax, United States of America: ICF International. | GHDx |
| **20450** | Nepal | 241 | 0 | 1724 | Nepal Demographic and Health Survey 2001 | 2001 | Macro International, Inc, Ministry of Health and Population (Nepal), New ERA. Nepal Demographic and Health Survey 2001. Fairfax, United States of America: ICF International. | GHDx |

| | | | | | | | | |
|---|---|---|---|---|---|---|---|---|
| **20462** | Nepal | 260 | 0 | 4497 | Nepal Demographic and Health Survey 2006 | 2006 | Macro International, Inc, Ministry of Health and Population (Nepal), New ERA. Nepal Demographic and Health Survey 2006. Fairfax, United States of America: ICF International. | GHDx |
| **21240** | Nepal | 289 | 0 | 4200 | Nepal Demographic and Health Survey 2011 | 2011 | ICF Macro, Ministry of Health and Population (Nepal), New ERA. Nepal Demographic and Health Survey 2011. Fairfax, United States of America: ICF International. | GHDx |
| **39999** | Nepal | 0 | 24 | 2137 | Nepal Multiple Indicator Cluster Survey 2010 | 2010 | United Nations Children's Fund (UNICEF), Central Bureau of Statistics (Nepal). Nepal Multiple Indicator Cluster Survey 2010. New York, United States: United Nations Children's Fund (UNICEF). | GHDx |
| **162317** | Nepal | 483 | 0 | 2904 | Nepal Multiple Indicator Cluster Survey 2014 | 2014 | Central Bureau of Statistics (Nepal), United Nations Children's Fund (UNICEF). Nepal Multiple Indicator Cluster Survey 2014. New York, United States of America: United Nations Children's Fund (UNICEF), 2015. | GHDx |
| **286782** | Nepal | 378 | 0 | 2504 | Nepal Demographic and Health Survey 2016-2017 | 2016-2017 | ICF International, Ministry of Health (Nepal), New ERA. Nepal Demographic and Health Survey 2016-2017. Fairfax, United States of America: ICF International, 2017. | GHDx |
| **9270** | Nicaragua | 619 | 0 | 3140 | Nicaragua Reproductive Health Survey 2006-2007 | 2006-2007 | Division of Reproductive Health, Centers for Disease Control and Prevention (CDC), National Institute for Development Information (Nicaragua). Nicaragua Reproductive Health Survey 2006-2007. Managua, Nicaragua: National Institute for Development Information (Nicaragua). | GHDx |
| **20499** | Niger | 0 | 8 | 4923 | Niger Demographic and Health Survey 2006 | 2006 | Department of Statistics and National Accounts (Niger), Macro International, Inc. Niger Demographic and Health Survey 2006. Fairfax, United States of America: ICF International. | GHDx |
| **74393** | Niger | 0 | 8 | 6906 | Niger Demographic and Health Survey 2012 | 2012 | ICF International, Ministry of Public Health (Niger), National Institute of Statistics (Niger). Niger Demographic and Health Survey 2012. Fairfax, United States of America: ICF International. | GHDx |

| | | | | | | | | |
|---|---|---|---|---|---|---|---|---|
| **9516** | Nigeria | 0 | 37 | 9891 | Nigeria Multiple Indicator Cluster Survey 2007 | 2007 | United Nations Children's Fund (UNICEF), National Bureau of Statistics (Nigeria). Nigeria Multiple Indicator Cluster Survey 2007. New York, United States: United Nations Children's Fund (UNICEF). | GHDx |
| **20567** | Nigeria | 357 | 0 | 2961 | Nigeria Demographic and Health Survey 2003 | 2003 | Department for International Development (DFiD) (United Kingdom), National Population Commission of Nigeria, ORC Macro, United Nations Children's Fund (UNICEF), United Nations Population Fund (UNFPA). Nigeria Demographic and Health Survey 2003. Fairfax, United States of America: ICF International. | GHDx |
| **21433** | Nigeria | 885 | 0 | 14647 | Nigeria Demographic and Health Survey 2008 | 2008 | Macro International, Inc, National Population Commission of Nigeria. Nigeria Demographic and Health Survey 2008. Fairfax, United States of America: ICF International, 2009. | GHDx |
| **24890** | Nigeria | 0 | 5 | 7 | Nigeria General Household Survey 2007 | 2007 | Central Bank of Nigeria, National Bureau of Statistics (Nigeria), Nigerian Communications Commission (NCC). Nigeria General Household Survey 2007. Abuja, Nigeria: National Bureau of Statistics (Nigeria). | GHDx |
| **76703** | Nigeria | 0 | 37 | 14503 | Nigeria Multiple Indicator Cluster Survey 2011 | 2011 | National Bureau of Statistics (Nigeria), United Nations Children's Fund (UNICEF). Nigeria Multiple Indicator Cluster Survey 2011. New York, United States of America: United Nations Children's Fund (UNICEF), 2013. | GHDx |
| **77390** | Nigeria | 889 | 0 | 16651 | Nigeria Demographic and Health Survey 2013 | 2013 | ICF International, National Population Commission of Nigeria. Nigeria Demographic and Health Survey 2013. Fairfax, United States of America: ICF International. | GHDx |
| **151797** | Nigeria | 178 | 0 | 261 | Nigeria General Household Survey 2012-2013 | 2012-2013 | National Bureau of Statistics (Nigeria). Nigeria General Household Survey 2012-2013. Washington DC, United States of America: World Bank. | GHDx |
| **218613** | Nigeria | 2164 | 0 | 10935 | Nigeria Multiple Indicator | 2016-2017 | National Agency for the Control of AIDS (Nigeria), National Bureau of Statistics (Nigeria), National Primary Health Care Development Agency (NPHCDA) (Nigeria), | GHDx |

| | | | | | | | | |
|---|---|---|---|---|---|---|---|---|
| | | | | | Cluster Survey with National Immunization Coverage Survey Supplement 2016-2017 | | United Nations Children's Fund (UNICEF). Nigeria Multiple Indicator Cluster Survey with National Immunization Coverage Survey Supplement 2016-2017. New York, United States of America: United Nations Children's Fund (UNICEF), 2018. | |
| **408484** | Nigeria | 1349 | 0 | 11525 | Nigeria Demographic and Health Survey 2018 | 2018 | Federal Ministry of Health (Nigeria), ICF International, National Population Commission (NPC). Nigeria Demographic and Health Survey 2018. Fairfax, United States of America: ICF International, 2019. | GHDx |
| **9720** | Pakistan | 0 | 6 | 2304 | Pakistan Integrated Household Survey/ Household Integrated Economic Survey 2001-2002 | 2001-2002 | Federal Bureau of Statistics (Pakistan). Pakistan Integrated Household Survey 2001-2002. Islamabad, Pakistan: Federal Bureau of Statistics (Pakistan). | GHDx |
| **9951** | Pakistan | 0 | 4 | 10128 | Pakistan Social and Living Standards Measurement Survey/ Household Integrated Economic Survey 2004-2005 | 2004-2005 | Federal Bureau of Statistics (Pakistan). Pakistan Social and Living Standards Measurement Survey 2004-2005. Islamabad, Pakistan: Federal Bureau of Statistics (Pakistan). | GHDx |
| **20595** | Pakistan | 929 | 0 | 5797 | Pakistan Demographic and Health Survey 2006-2007 | 2006-2007 | Macro International, Inc, National Institute of Population Studies (Pakistan). Pakistan Demographic and Health Survey 2006-2007. Fairfax, United States of America: ICF International. | GHDx |

| | | | | | | | | |
|---|---|---|---|---|---|---|---|---|
| **24818** | Pakistan | 0 | 4 | 10788 | Pakistan Social and Living Standards Measurement Survey/ Household Integrated Economic Survey 2005-2006 | 2005-2006 | Federal Bureau of Statistics (Pakistan). Pakistan Social and Living Standards Measurement Survey 2005-2006. Islamabad, Pakistan: Federal Bureau of Statistics (Pakistan). | GHDx |
| **30634** | Pakistan | 0 | 4 | 10111 | Pakistan Social and Living Standards Measurement Survey/ Household Integrated Economic Survey 2007-2008 | 2007-2008 | Federal Bureau of Statistics (Pakistan). Pakistan Social and Living Standards Measurement Survey 2007-2008. Islamabad, Pakistan: Federal Bureau of Statistics (Pakistan). | GHDx |
| **77521** | Pakistan | 0 | 119 | 8150 | Pakistan Demographic and Health Survey 2012-2013 | 2012-2013 | ICF International, National Institute of Population Studies (Pakistan), Pakistan Bureau of Statistics. Pakistan Demographic and Health Survey 2012-2013. Fairfax, United States of America: ICF International. | GHDx |
| **104236** | Pakistan | 0 | 145 | 25306 | Pakistan - Punjab Multiple Indicator Cluster Survey 2011 | 2011 | Bureau of Statistics Punjab (Pakistan), United Nations Children's Fund (UNICEF), United Nations Development Programme (UNDP). Pakistan - Punjab Multiple Indicator Cluster Survey 2011. New York, United States of America: United Nations Children's Fund (UNICEF), 2013. | GHDx |
| **232763** | Pakistan | 0 | 28 | 5887 | Pakistan - Sindh Multiple Indicator Cluster Survey 2014 | 2014 | Bureau of Statistics, Planning and Development Department, Government of Sindh (Pakistan), Global Alliance for Improved Nutrition (GAIN), Pakistan Council of Research in Water Resource (PCRWR), United Nations Children's Fund (UNICEF). Pakistan - Sindh Multiple | GHDx |

| | | | | | | | | |
|---|---|---|---|---|---|---|---|---|
| | | | | | | | Indicator Cluster Survey 2014. Fairfax, United States of America: ICF International, 2016. | |
| **236266** | Pakistan | 0 | 36 | 10479 | Pakistan - Punjab Multiple Indicator Cluster Survey 2014 | 2014 | Bureau of Statistics Punjab (Pakistan), United Nations Children's Fund (UNICEF). Pakistan - Punjab Multiple Indicator Cluster Survey 2014. New York, United States of America: United Nations Children's Fund (UNICEF), 2015. | GHDx |
| **261883** | Pakistan | 1 | 0 | 256 | Pakistan - Naushahro Feroze Malnutrition and Enteric Disease Study 2009-2014 | 2009-2014 | Aga Khan University, Fogarty International Center, National Institutes of Health (NIH), Foundation for the National Institutes of Health (FNIH). Pakistan - Naushahro Feroze Malnutrition and Enteric Disease Study 2009-2014. | GHDx |
| **286783** | Pakistan | 556 | 0 | 6058 | Pakistan Demographic and Health Survey 2017-2018 | 2017-2018 | ICF International, Ministry of National Health Services, Regulations & Coordination (Pakistan), National Institute of Population Studies (Pakistan). Pakistan Demographic and Health Survey 2017-2018. Fairfax, United States of America: ICF International, 2018. | GHDx |
| **9999** | Palestine | 0 | 16 | 6136 | Palestine Family Health Survey 2006-2007 | 2006-2007 | League of Arab States, Palestinian Central Bureau of Statistics, United Nations Children's Fund (UNICEF). Palestine Family Health Survey 2006-2007. | GHDx |
| **125591** | Palestine | 0 | 16 | 3909 | Palestine Multiple Indicator Cluster Survey 2010 | 2010 | Ministry of Health (Palestine), Palestinian Central Bureau of Statistics, United Nations Children's Fund (UNICEF), United Nations Population Fund (UNFPA). Palestine Multiple Indicator Cluster Survey 2010. New York, United States of America: United Nations Children's Fund (UNICEF), 2014. | GHDx |
| **161590** | Palestine | 0 | 16 | 1540 | Palestine Multiple Indicator Cluster Survey 2014 | 2014 | Ministry of Health (Palestine), Palestinian Central Bureau of Statistics, United Nations Children's Fund (UNICEF). Palestine Multiple Indicator Cluster Survey 2014. New York, United States of America: United Nations Children's Fund (UNICEF), 2015. | GHDx |

| | | | | | | | | |
|---|---|---|---|---|---|---|---|---|
| **161587** | Panama | 0 | 509 | 4458 | Panama Multiple Indicator Cluster Survey 2013 | 2013 | National Institute of Statistics and Census (Panama), United Nations Children's Fund (UNICEF). Panama Multiple Indicator Cluster Survey 2013. New York, United States of America: United Nations Children's Fund (UNICEF). | GHDx |
| **44870** | Papua New Guinea | 0 | 4 | 1255 | Papua New Guinea Demographic and Health Survey 2006-2007 | 2006-2007 | National Statistical Office (Papua New Guinea), National Statistics Office (Philippines). Papua New Guinea Demographic and Health Survey 2006-2007. | GHDx |
| **426238** | Papua New Guinea | 706 | 0 | 3540 | Papua New Guinea Demographic and Health Survey 2016-2018 | 2016-2018 | ICF International, National Statistical Office (Papua New Guinea). Papua New Guinea Demographic and Health Survey 2016-2018. Fairfax, United States of America: ICF International, 2019. | GHDx |
| **10370** | Paraguay | 0 | 16 | 1534 | Paraguay Reproductive Health Survey 2004 | 2004 | Division of Reproductive Health-Centers for Disease Control and Prevention (CDC). (2005): Paraguay Reproductive Health Survey 2004. Asunción, Paraguay, Paraguayan Center for Population Studies (CEPEP). | GHDx |
| **27525** | Paraguay | 0 | 55 | 939 | Paraguay Reproductive Health Survey 2008 | 2008 | Paraguay Center for Population Studies (CEPEP). Paraguay Reproductive Health Survey 2008. Asunción, Paraguay: Paraguayan Center for Population Studies (CEPEP). | GHDx |
| **41830** | Paraguay | 0 | 371 | 1694 | Paraguay Permanent Household Survey 2005 | 2005 | Department of Statistics, Surveys and Censuses (Paraguay). Paraguay Permanent Household Survey 2005. Asunción, Paraguay: Department of Statistics, Surveys and Censuses (Paraguay). | GHDx |
| **41837** | Paraguay | 0 | 13 | 812 | Paraguay Permanent Household Survey 2006 | 2006 | Department of Statistics, Surveys and Censuses (Paraguay). Paraguay Permanent Household Survey 2006. Asunción, Paraguay: Department of Statistics, Surveys and Censuses (Paraguay). | GHDx |
| **324470** | Paraguay | 0 | 9 | 896 | Paraguay Multiple Indicator | 2016 | General Directorate of Statistics, Surveys and Censuses (Paraguay), Ministry of Public Health and Social Welfare (Paraguay), United Nations Children's Fund (UNICEF). | GHDx |

| | | | | | | | | |
|---|---|---|---|---|---|---|---|---|
| | | | | | Cluster Survey 2016 | | Paraguay Multiple Indicator Cluster Survey 2016. New York, United States of America: United Nations Children's Fund (UNICEF), 2017. | |
| **210182** | Peru | 1150 | 0 | 5566 | Peru Continuous Demographic and Health Survey 2014 - INEI | 2014 | ICF International, Ministry of Health (Peru), National Institute of Statistics and Informatics (Peru), National Police of Peru (PNP). Peru Continuous Demographic and Health Survey 2014 - INEI. Lima, Peru: National Institute of Statistics and Informatics (Peru), 2015. | GHDx |
| **210231** | Peru | 0 | 608 | 5504 | Peru Continuous Demographic and Health Survey 2013 - INEI | 2013 | ICF International, National Institute of Statistics and Informatics (Peru). Peru Continuous Demographic and Health Survey 2013 - INEI. Lima, Peru: National Institute of Statistics and Informatics (Peru), 2014. | GHDx |
| **270404** | Peru | 1103 | 0 | 6127 | Peru Continuous Demographic and Health Survey 2009 | 2009 | National Institute of Statistics and Informatics (Peru), ORC Macro. Peru Continuous Demographic and Health Survey 2009. Fairfax, United States of America: ICF International. | GHDx |
| **270469** | Peru | 0 | 24 | 7344 | Peru Continuous Demographic and Health Survey 2010 | 2010 | National Institute of Statistics and Informatics (Peru). Peru Continuous Demographic and Health Survey 2010. Fairfax, United States of America: ICF International. | GHDx |
| **270470** | Peru | 0 | 24 | 7275 | Peru Continuous Demographic and Health Survey 2011 | 2011 | Macro International, Inc, National Institute of Statistics and Informatics (Peru). Peru Continuous Demographic and Health Survey 2011. Fairfax, United States of America: ICF International. | GHDx |
| **270471** | Peru | 0 | 24 | 7614 | Peru Continuous Demographic and Health Survey 2012 | 2012 | Macro International, Inc, National Institute of Statistics and Informatics (Peru). Peru Continuous Demographic and Health Survey 2012. Fairfax, United States of America: ICF International. | GHDx |

| | | | | | | | | |
|---|---|---|---|---|---|---|---|---|
| **303663** | Peru | 1573 | 0 | 11290 | Peru Demographic and Family Health Survey 2015 | 2015 | National Institute of Statistics and Informatics (Peru). Peru Demographic and Family Health Survey 2015. Lima, Peru: National Institute of Statistics and Informatics (Peru), 2017. | GHDx |
| **303664** | Peru | 19 | 0 | 105 | Peru Demographic and Family Health Survey 2016 | 2016 | National Institute of Statistics and Informatics (Peru). Peru Demographic and Family Health Survey 2016. Lima, Peru: National Institute of Statistics and Informatics (Peru), 2017. | GHDx |
| **407869** | Peru | 3681 | 0 | 14487 | Peru Demographic and Family Health Survey 2018 | 2018 | National Institute of Statistics and Informatics (Peru). Peru Demographic and Family Health Survey 2018. Lima, Peru: National Institute of Statistics and Informatics (Peru), 2019. | GHDx |
| **20699** | Philippines | 788 | 0 | 4173 | Philippines Demographic and Health Survey 2003 | 2003 | Macro International, Inc, National Statistics Office (Philippines). Philippines Demographic and Health Survey 2003. Fairfax, United States of America: ICF International. | GHDx |
| **21421** | Philippines | 739 | 0 | 3831 | Philippines Demographic and Health Survey 2008 | 2008 | Macro International, Inc, National Statistics Office (Philippines). Philippines Demographic and Health Survey 2008. Fairfax, United States of America: ICF International, 2010. | GHDx |
| **142943** | Philippines | 0 | 17 | 4202 | Philippines Demographic and Health Survey 2013 | 2013 | ICF International, Philippines Statistics Authority. Philippines Demographic and Health Survey 2013. Fairfax, United States of America: ICF International, 2014. | GHDx |
| **20740** | Rwanda | 456 | 0 | 8235 | Rwanda Demographic and Health Survey 2005 | 2005 | Macro International, Inc, National Institute of Statistics of Rwanda. Rwanda Demographic and Health Survey 2005. Fairfax, United States of America: ICF International. | GHDx |
| **21222** | Rwanda | 246 | 0 | 5328 | Rwanda Interim Demographic and Health | 2007-2008 | Macro International, Inc, Ministry of Health (Rwanda), National Institute of Statistics of Rwanda. Rwanda Interim Demographic and Health Survey 2007-2008. Fairfax, United States of America: ICF International. | GHDx |

| | | | | | | | | |
|---|---|---|---|---|---|---|---|---|
| | | | | | Survey 2007-2008 | | | |
| **56040** | Rwanda | 492 | 0 | 8877 | Rwanda Demographic and Health Survey 2010-2011 | 2010-2011 | ICF Macro, Ministry of Health (Rwanda), National Institute of Statistics of Rwanda. Rwanda Demographic and Health Survey 2010-2011. Fairfax, United States of America: ICF International. | GHDx |
| **151437** | Rwanda | 0 | 5 | 10988 | Rwanda Integrated Household Living Conditions Survey 2010-2011 | 2010-2011 | National Institute of Statistics of Rwanda. Rwanda Integrated Household Living Conditions Survey 2010-2011. Kigali, Rwanda: National Institute of Statistics of Rwanda. | GHDx |
| **157063** | Rwanda | 492 | 0 | 8052 | Rwanda Demographic and Health Survey 2014-2015 | 2014-2015 | ICF International, Ministry of Health (Rwanda), National Institute of Statistics of Rwanda. Rwanda Demographic and Health Survey 2014-2015. Fairfax, United States of America: ICF International, 2016. | GHDx |
| **26866** | Sao Tome and Principe | 0 | 7 | 1125 | Sao Tome and Principe Demographic and Health Survey 2008-2009 | 2008-2009 | ICF Macro, Ministry of Health (Sao Tome and Principe), National Institute of Statistics (Sao Tome and Principe). Sao Tome and Principe Demographic and Health Survey 2008-2009. Fairfax, United States of America: ICF International. | GHDx |
| **214640** | Sao Tome and Principe | 0 | 7 | 786 | Sao Tome and Principe Multiple Indicator Cluster Survey 2014 | 2014 | Global Fund to Fight Aids Tuberculosis and Malaria (GFATM), ICF International, National Center for Endemic Diseases (CNE) (Sao Tome and Principe), National Institute of Statistics (Sao Tome and Principe), United Nations Children's Fund (UNICEF), United Nations Development Programme (UNDP). Sao Tome and Principe Multiple Indicator Cluster Survey 2014. New York, United States of America: United Nations Children's Fund (UNICEF), 2016. | GHDx |
| **26855** | Senegal | 366 | 0 | 5667 | Senegal Demographic | 2005 | Ministry of Health and Prevention (Senegal), Research Center for Human Development (Senegal). Senegal | GHDx |

| | | | | | | | | |
|---|---|---|---|---|---|---|---|---|
| | | | | | and Health Survey 2005 | | Demographic and Health Survey 2005. Fairfax, United States of America: ICF International. | |
| **56063** | Senegal | 385 | 0 | 6884 | Senegal Demographic and Health Survey 2010-2011 | 2010-2011 | Center for Research in Human Development (CRDH), Cheikh Anta Diop University, Hospital Aristide Le Dantec, ICF Macro, National Agency of Statistics and Demography (Senegal). Senegal Demographic and Health Survey 2010-2011. Fairfax, United States of America: ICF International. | GHDx |
| **111432** | Senegal | 200 | 0 | 3969 | Senegal Continuous Demographic and Health Survey 2012-2013 | 2012-2013 | ICF International, Ministry of Health and Social Action (Senegal), National Agency of Statistics and Demography (Senegal). Senegal Continuous Demographic and Health Survey 2012-2013. Fairfax, United States of America: ICF International. | GHDx |
| **191270** | Senegal | 196 | 0 | 3891 | Senegal Continuous Demographic and Health Survey 2014 | 2014 | Cheikh Anta Diop University, ICF International, National Agency of Statistics and Demography (Senegal). Senegal Continuous Demographic and Health Survey 2014. Fairfax, United States of America: ICF International. | GHDx |
| **218592** | Senegal | 214 | 0 | 3969 | Senegal Continuous Demographic and Health Survey 2015 | 2015 | Cheikh Anta Diop University, ICF International, National Agency of Statistics and Demography (Senegal). Senegal Continuous Demographic and Health Survey 2015. Fairfax, United States of America: ICF International, 2016. | GHDx |
| **286772** | Senegal | 214 | 0 | 3856 | Senegal Continuous Demographic and Health Survey 2016 | 2016 | ICF International, Ministry of Health and Social Action (Senegal), National Agency of Statistics and Demography (Senegal). Senegal Continuous Demographic and Health Survey 2016. Fairfax, United States of America: ICF International, 2017. | GHDx |
| **287639** | Senegal | 0 | 4 | 1683 | Senegal - Dakar Urban Multiple Indicator Cluster Survey 2015-2016 | 2015-2016 | National Agency of Statistics and Demography (Senegal), United Nations Children's Fund (UNICEF). Senegal - Dakar Urban Multiple Indicator Cluster Survey 2015-2016. New York, United States of America: United Nations Children's Fund (UNICEF), 2018. | GHDx |
| **353526** | Senegal | 0 | 14 | 4601 | Senegal Continuous | 2017 | ICF International, Ministry of Health and Social Action (Senegal), National Agency of Statistics and Demography | GHDx |

| | | | | | | | | |
|---|---|---|---|---|---|---|---|---|
| | | | | | Demographic and Health Survey 2017 | | (Senegal), Unit for the Fight Against Malnutrition (Senegal). Senegal Continuous Demographic and Health Survey 2017. Fairfax, United States of America: ICF International, 2018. | |
| **450419** | Senegal | 0 | 14 | 2534 | Senegal Continuous Demographic and Health Survey 2018 | 2018 | Directorate of Forecasting and Statistics, Ministry of the Economy, Finance and Planning (Senegal), ICF International, Ministry of Health and Social Action (Senegal), United States Agency for International Development (USAID). Senegal Continuous Demographic and Health Survey 2018. Fairfax, United States of America: ICF International, 2020. | GHDx |
| **11649** | Sierra Leone | 0 | 14 | 3216 | Sierra Leone Multiple Indicator Cluster Survey 2005 | 2005 | United Nations Children's Fund (UNICEF), Statistics Sierra Leone. Sierra Leone Multiple Indicator Cluster Survey 2005. New York, United States: United Nations Children's Fund (UNICEF). | GHDx |
| **21258** | Sierra Leone | 348 | 0 | 2823 | Sierra Leone Demographic and Health Survey 2008 | 2008 | Macro International, Inc, Statistics Sierra Leone. Sierra Leone Demographic and Health Survey 2008. Fairfax, United States of America: ICF International. | GHDx |
| **131467** | Sierra Leone | 435 | 0 | 6222 | Sierra Leone Demographic and Health Survey 2013 | 2013 | ICF International, Ministry of Health and Sanitation (Sierra Leone), Statistics Sierra Leone. Sierra Leone Demographic and Health Survey 2013. Fairfax, United States of America: ICF International, 2014. | GHDx |
| **218619** | Sierra Leone | 0 | 14 | 6874 | Sierra Leone Multiple Indicator Cluster Survey 2017 | 2017 | Statistics Sierra Leone, United Nations Children's Fund (UNICEF). Sierra Leone Multiple Indicator Cluster Survey 2017. New York, United States of America: United Nations Children's Fund (UNICEF), 2018. | GHDx |
| **11774** | Somalia | 0 | 18 | 3554 | Somalia Multiple Indicator Cluster Survey 2006 | 2006 | Pan Arab Project for Family Health (PAPFAM), United Nations Children's Fund (UNICEF). Somalia Multiple Indicator Cluster Survey 2006. New York, United States of America: United Nations Children's Fund (UNICEF). | GHDx |
| **91507** | Somalia | 244 | 0 | 2562 | Somalia - Somaliland | 2011 | Ministry of National Planning and Development (Somaliland), United Nations Children's Fund (UNICEF). | GHDx |

| | | | | | | | | |
|---|---|---|---|---|---|---|---|---|
| | | | | | Multiple Indicator Cluster Survey 2011 | | Somalia - Somaliland Multiple Indicator Cluster Survey 2011. New York, United States of America: United Nations Children's Fund (UNICEF), 2015. | |
| **91508** | Somalia | 0 | 3 | 792 | Somalia - Northeast Zone Multiple Indicator Cluster Survey 2011 | 2011 | Puntland Ministry of Planning and International Cooperation (Somalia), United Nations Children's Fund (UNICEF). Somalia - Northeast Zone Multiple Indicator Cluster Survey 2011. New York, United States of America: United Nations Children's Fund (UNICEF), 2015. | GHDx |
| **20798** | South Africa | 0 | 9 | 436 | South Africa Demographic and Health Survey 2003-2004 | 2003-2004 | Department of Health (South Africa), Macro International, Inc, South African Medical Research Council. South Africa Demographic and Health Survey 2003-2004. | GHDx |
| **157064** | South Africa | 540 | 0 | 1379 | South Africa Demographic and Health Survey 2016 | 2016 | Department of Health (South Africa), ICF International, South African Medical Research Council, Statistics South Africa. South Africa Demographic and Health Survey 2016. Fairfax, United States of America: ICF International, 2019. | GHDx |
| **18815** | Sri Lanka | 1436 | 342 | 4223 | Sri Lanka Demographic and Health Survey 2006-2007 | 2006-2007 | Department of Census and Statistics (Sri Lanka), Macro International, Inc. Sri Lanka Demographic and Health Survey 2006-2007. 2009. | GHDx |
| **326837** | Sri Lanka | 0 | 25 | 4883 | Sri Lanka Demographic and Health Survey 2016 | 2016 | Department of Census and Statistics (Sri Lanka), ICF International. Sri Lanka Demographic and Health Survey 2016. | GHDx |
| **32189** | Sudan | 0 | 10 | 1420 | Sudan - South Multiple Indicator Cluster Survey 2010 | 2010 | Central Bureau of Statistics (Sudan), Federal Ministry of Health (Sudan), Government of Sudan, Ministry of Health (South Sudan), Southern Sudan Centre for Census, Statistics and Evaluation. Sudan - South Multiple Indicator Cluster Survey 2010. New York, United States of America: United Nations Children's Fund (UNICEF), 2015. | GHDx |

| | | | | | | | | |
|---|---|---|---|---|---|---|---|---|
| **153643** | Sudan | 0 | 15 | 3369 | Sudan - North Multiple Indicator Cluster Survey 2010 | 2010 | Central Bureau of Statistics (Sudan), Ministry of Health (South Sudan). Sudan - North Multiple Indicator Cluster Survey 2010. New York, United States of America: United Nations Children's Fund (UNICEF), 2015. | GHDx |
| **200617** | Sudan | 0 | 18 | 6835 | Sudan Multiple Indicator Cluster Survey 2014 | 2014 | Central Bureau of Statistics (Sudan), Federal Ministry of Health (Sudan), United Nations Children's Fund (UNICEF). Sudan Multiple Indicator Cluster Survey 2014. New York, United States of America: United Nations Children's Fund (UNICEF), 2016. | GHDx |
| **12289** | Suriname | 0 | 5 | 1333 | Suriname Multiple Indicator Cluster Survey 2006 | 2006 | General Statistical Office (Suriname), United Nations Children's Fund (UNICEF). Suriname Multiple Indicator Cluster Survey 2006. New York, United States of America: United Nations Children's Fund (UNICEF). | GHDx |
| **81203** | Suriname | 0 | 10 | 3384 | Suriname Multiple Indicator Cluster Survey 2010 | 2010 | General Bureau of Statistics (Suriname), Ministry of Planning and Development Cooperation (Suriname), Ministry of Social Affairs and Housing (Suriname), United Nations Children's Fund (UNICEF). Suriname Multiple Indicator Cluster Survey 2010. New York, United States of America: United Nations Children's Fund (UNICEF), 2013. | GHDx |
| **427983** | Suriname | 0 | 10 | 1613 | Suriname Multiple Indicator Cluster Survey 2018 | 2018 | General Bureau of Statistics (Suriname), Ministry of Planning and Development Cooperation (Suriname), Ministry of Social Affairs and Housing (Suriname), United Nations Children's Fund (UNICEF). Suriname Multiple Indicator Cluster Survey 2018. New York, United States of America: United Nations Children's Fund (UNICEF), 2019. | GHDx |
| **20829** | Swaziland | 266 | 0 | 2768 | Swaziland Demographic and Health Survey 2006-2007 | 2006-2007 | Central Statistical Office (Swaziland), Macro International, Inc. Swaziland Demographic and Health Survey 2006-2007. Fairfax, United States of America: ICF International. | GHDx |
| **30325** | Swaziland | 0 | 4 | 1555 | Swaziland Multiple | 2010 | Central Statistical Office (Swaziland), United Nations Children's Fund (UNICEF). Swaziland Multiple Indicator | GHDx |

| | | | | | | | | |
|---|---|---|---|---|---|---|---|---|
| | | | | | Indicator Cluster Survey 2010 | | Cluster Survey 2010. New York, United States of America: United Nations Children's Fund (UNICEF). | |
| **200707** | Swaziland | 0 | 4 | 2090 | Swaziland Multiple Indicator Cluster Survey 2014 | 2014 | Central Statistical Office (Swaziland), United Nations Children's Fund (UNICEF), United Nations Educational, Scientific and Cultural Organization (UNESCO), United Nations Population Fund (UNFPA). Swaziland Multiple Indicator Cluster Survey 2014. New York, United States of America: United Nations Children's Fund (UNICEF), 2016. | GHDx |
| **12379** | Syria | 0 | 1 | 494 | Syria Family Health Survey 2001 | 2001 | Central Bureau of Statistics (Syria), League of Arab States. Syria Family Health Survey 2001. | GHDx |
| **12399** | Syria | 0 | 14 | 1152 | Syria Multiple Indicator Cluster Survey 2006 | 2006 | United Nations Children's Fund (UNICEF), Central Bureau of Statistics (Syria), Ministry of Health (Syria), Pan Arab Project for Family Health (PAPFAM). Syria Multiple Indicator Cluster Survey 2006. New York, United States: United Nations Children's Fund (UNICEF). | GHDx |
| **12608** | Tajikistan | 0 | 5 | 3251 | Tajikistan Multiple Indicator Cluster Survey 2005 | 2005 | United Nations Children's Fund (UNICEF), State Committee on Statistics of the Republic of Tajikistan. Tajikistan Multiple Indicator Cluster Survey 2005. New York, United States: United Nations Children's Fund (UNICEF). | GHDx |
| **74460** | Tajikistan | 340 | 0 | 3835 | Tajikistan Demographic and Health Survey 2012 | 2012 | ICF International, Ministry of Health (Tajikistan), Statistical Agency under the President of the Republic of Tajikistan. Tajikistan Demographic and Health Survey 2012. Fairfax, United States of America: ICF International, 2013. | GHDx |
| **341838** | Tajikistan | 338 | 0 | 1784 | Tajikistan Demographic and Health Survey 2017 | 2017 | ICF International, Statistical Agency under the President of the Republic of Tajikistan. Tajikistan Demographic and Health Survey 2017. Fairfax, United States of America: ICF International, 2018. | GHDx |
| **20875** | Tanzania | 0 | 26 | 4889 | Tanzania Demographic and Health | 2004-2005 | Macro International, Inc, National Bureau of Statistics (Tanzania). Tanzania Demographic and Health Survey 2004-2005. Fairfax, United States of America: ICF International. | GHDx |

| | | | | | | | | |
|---|---|---|---|---|---|---|---|---|
| | | | | | Survey 2004-2005 | | | |
| **21331** | Tanzania | 457 | 0 | 4592 | Tanzania Demographic and Health Survey 2009-2010 | 2009-2010 | ICF Macro, National Bureau of Statistics (Tanzania). Tanzania Demographic and Health Survey 2009-2010. Fairfax, United States of America: ICF International. | GHDx |
| **218593** | Tanzania | 605 | 0 | 4226 | Tanzania Demographic and Health Survey 2015-2016 | 2015-2016 | ICF International, Ministry of Health (Zanzibar), Ministry of Health, Community Development, Gender, Elderly and Children (MoHCDEC) (Tanzania), National Bureau of Statistics (Tanzania), Office of the Chief Government Statistician (OCGS) (Zanzibar). Tanzania Demographic and Health Survey 2015-2016. Fairfax, United States of America: ICF International, 2016. | GHDx |
| **12732** | Thailand | 0 | 4 | 5626 | Thailand Multiple Indicator Cluster Survey 2005-2006 | 2005-2006 | National Statistical Office (Thailand), United Nations Children's Fund (UNICEF). Thailand Multiple Indicator Cluster Survey 2005-2006. New York, United States of America: United Nations Children's Fund (UNICEF). | GHDx |
| **148649** | Thailand | 0 | 5 | 6171 | Thailand Multiple Indicator Cluster Survey 2012 | 2012 | College of Population Studies, Chulalongkorn University (Thailand), Institute for Population and Social Research, Mahidol University (Thailand), International Health Policy Program (Thailand), Ministry of Education (Thailand), Ministry of Public Health (Thailand), Ministry of Social Development and Human Security (MSDHS) (Thailand), National Health Security Office (Thailand), National Statistical Office (Thailand), Thai Health Promotion Foundation, United Nations Children's Fund (UNICEF). Thailand Multiple Indicator Cluster Survey 2012. New York, United States of America: United Nations Children's Fund (UNICEF), 2016. | GHDx |
| **296646** | Thailand | 0 | 5 | 8084 | Thailand Multiple Indicator Cluster Survey 2015-2016 | 2015-2016 | National Health Security Office (Thailand), National Statistical Office (Thailand), United Nations Children's Fund (UNICEF). Thailand Multiple Indicator Cluster Survey 2015-2016. New York, United States of America: United Nations Children's Fund (UNICEF), 2018. | GHDx |

| | | | | | | | | |
|---|---|---|---|---|---|---|---|---|
| **331377** | Thailand | 0 | 1 | 822 | Thailand - Bangkok Small Community Multiple Indicator Cluster Survey 2016 | 2016 | National Health Security Office (Thailand), National Statistical Office (Thailand), United Nations Children's Fund (UNICEF). Thailand - Bangkok Small Community Multiple Indicator Cluster Survey 2016. New York, United States of America: United Nations Children's Fund (UNICEF), 2018. | GHDx |
| **20888** | Timor-Leste | 0 | 163 | 3131 | Timor-Leste Demographic and Health Survey 2003 | 2003 | ACIL Australia Pty Ltd., Australian National University, Ministry of Health (Timor-Leste), National Statistics Directorate (Timor-Leste), University of Newcastle (Australia). Timor-Leste Demographic and Health Survey 2003. Newcastle, Australia: University of Newcastle (Australia). | GHDx |
| **21274** | Timor-Leste | 0 | 13 | 5673 | Timor-Leste Demographic and Health Survey 2009-2010 | 2009-2010 | ICF Macro, Ministry of Finance (Timor-Leste), National Statistics Directorate (Timor-Leste). Timor-Leste Demographic and Health Survey 2009-2010. Fairfax, United States of America: ICF International. | GHDx |
| **286785** | Timor-Leste | 452 | 0 | 2761 | Timor-Leste Demographic and Health Survey 2016 | 2016 | ICF International, National Statistics Directorate (Timor-Leste). Timor-Leste Demographic and Health Survey 2016. Fairfax, United States of America: ICF International, 2018. | GHDx |
| **12896** | Togo | 0 | 6 | 2453 | Togo Multiple Indicator Cluster Survey 2006 | 2006 | Directorate General of Statistics and National Accounting (Togo), United Nations Children's Fund (UNICEF). Togo Multiple Indicator Cluster Survey 2006. New York, United States: United Nations Children's Fund (UNICEF). | GHDx |
| **40021** | Togo | 0 | 6 | 2801 | Togo Multiple Indicator Cluster Survey 2010 | 2010 | Directorate General of Statistics and National Accounting (Togo), United Nations Children's Fund (UNICEF). Togo Multiple Indicator Cluster Survey 2010. New York, United States: United Nations Children's Fund (UNICEF). | GHDx |
| **77515** | Togo | 330 | 0 | 3934 | Togo Demographic and Health Survey 2013-2014 | 2013-2014 | Directorate General of Statistics and National Accounts (Togo), ICF International, Ministry of Health (Togo), Ministry of Planning, Development and Zoning (Togo). Togo Demographic and Health Survey 2013-2014. Fairfax, United States of America: ICF International, 2015. | GHDx |

| | | | | | | | | |
|---|---|---|---|---|---|---|---|---|
| **393869** | Togo | 0 | 6 | 1262 | Incomplete immunization among children aged 12-23 months in Togo: a multilevel analysis of individual and contextual factors | 2017 | Ekouevi DK, Gbeasor-Komlanvi FA, Yaya I, Zida-Compaore WI, Boko A, Sewu E, Lacle A, Ndibu N, Toke Y, Landoh DE. Incomplete immunization among children aged 12-23 months in Togo: a multilevel analysis of individual and contextual factors. BMC Public Health. 2018; 18(1): 952. | GHDx |
| **429991** | Togo | 396 | 0 | 1943 | Togo Multiple Indicator Cluster Survey 2017 | 2017 | Directorate General of Statistics and National Accounting (Togo), United Nations Children's Fund (UNICEF). Togo Multiple Indicator Cluster Survey 2010. New York, United States: United Nations Children's Fund (UNICEF). | GHDx |
| **12950** | Trinidad and Tobago | 0 | 15 | 1294 | Trinidad and Tobago Multiple Indicator Cluster Survey 2006 | 2006 | Central Statistical Office (Trinidad and Tobago) and United Nations Children's Fund (UNICEF). Trinidad and Tobago Multiple Indicator Cluster Survey 2006. New York, United States: United Nations Children's Fund (UNICEF). | GHDx |
| **76709** | Tunisia | 0 | 9 | 3179 | Tunisia Multiple Indicator Cluster Survey 2011-2012 | 2011-2012 | Ministry of Regional Development and Planning (Tunisia), National Institute of Statistics (Tunisia), United Nations Children's Fund (UNICEF). Tunisia Multiple Indicator Cluster Survey 2011-2012. New York, United States of America: United Nations Children's Fund (UNICEF), 2014. | GHDx |
| **13064** | Turkmenistan | 0 | 6 | 1479 | Turkmenistan Multiple Indicator Cluster Survey 2006 | 2006 | Ministry of Foreign Affairs (Turkmenistan), Ministry of Health and Medical Industry (Turkmenistan), National Institute of State Statistics and Information (Turkmenistan), United Nations Children's Fund (UNICEF). Turkmenistan Multiple Indicator Cluster Survey 2006. New York, United States of America: United Nations Children's Fund (UNICEF), 2016. | GHDx |
| **264583** | Turkmenistan | 0 | 6 | 955 | Turkmenistan Multiple | 2015-2016 | State Committee on Statistics of Turkmenistan, United Nations Children's Fund (UNICEF). Turkmenistan | GHDx |

| | | | | | | | | |
|---|---|---|---|---|---|---|---|---|
| | | | | | Indicator Cluster Survey 2015-2016 | | Multiple Indicator Cluster Survey 2015-2016. New York, United States of America: United Nations Children's Fund (UNICEF), 2017. | |
| **21014** | Uganda | 336 | 0 | 5324 | Uganda Demographic and Health Survey 2006 | 2006 | Macro International, Inc, Uganda Bureau of Statistics. Uganda Demographic and Health Survey 2006. Fairfax, United States of America: ICF International. | GHDx |
| **56021** | Uganda | 400 | 0 | 5567 | Uganda Demographic and Health Survey 2011 | 2011 | ICF Macro, Uganda Bureau of Statistics. Uganda Demographic and Health Survey 2011. Fairfax, United States of America: ICF International. | GHDx |
| **81004** | Uganda | 230 | 4 | 611 | Uganda Living Standards Measurement Survey - Integrated Survey on Agriculture 2009-2010 | 2009-2010 | Uganda Bureau of Statistics. Uganda Living Standards Measurement Survey - Integrated Survey on Agriculture 2009-2010. Washington DC, United States of America: World Bank. | GHDx |
| **93320** | Uganda | 0 | 80 | 5997 | Uganda National Service Delivery Survey 2008 | 2008 | Ministry of Public Service (Uganda), Uganda Bureau of Statistics. Uganda National Service Delivery Survey 2008. | GHDx |
| **142934** | Uganda | 264 | 0 | 427 | Uganda Living Standards Measurement Survey - Integrated Survey on Agriculture 2010-2011 | 2010-2011 | Uganda Bureau of Statistics. Uganda Living Standards Measurement Survey - Integrated Survey on Agriculture 2010-2011. Washington DC, United States of America: World Bank. | GHDx |
| **142935** | Uganda | 307 | 0 | 649 | Uganda Living Standards Measurement Survey - | 2011-2012 | Uganda Bureau of Statistics. Uganda Living Standards Measurement Survey - Integrated Survey on Agriculture 2011-2012. Washington DC, United States of America: World Bank. | GHDx |

| | | | | | | | | |
|---|---|---|---|---|---|---|---|---|
| | | | | | Integrated Survey on Agriculture 2011-2012 | | | |
| **264959** | Uganda | 0 | 287 | 505 | Uganda Living Standards Measurement Survey - Integrated Survey on Agriculture 2013-2014 | 2013-2014 | Government of the Netherlands, Uganda Bureau of Statistics, World Bank. Uganda Living Standards Measurement Survey - Integrated Survey on Agriculture 2013-2014. Washington DC, United States of America: World Bank. | GHDx |
| **286780** | Uganda | 684 | 0 | 7479 | Uganda Demographic and Health Survey 2016 | 2016 | ICF International, Uganda Bureau of Statistics. Uganda Demographic and Health Survey 2016. Fairfax, United States of America: ICF International, 2018. | GHDx |
| **132739** | Ukraine | 0 | 5 | 2574 | Ukraine Multiple Indicator Cluster Survey 2012 | 2012 | StatInform Consulting, State Statistics Service (Ukraine), Ukrainian Center for Social Reforms (UCSR), United Nations Children's Fund (UNICEF). Ukraine Multiple Indicator Cluster Survey 2012. New York, United States of America: United Nations Children's Fund (UNICEF), 2014. | GHDx |
| **13445** | Uzbekistan | 0 | 6 | 3087 | Uzbekistan Multiple Indicator Cluster Survey 2006 | 2006 | United Nations Children's Fund (UNICEF), State Committee of the Republic of Uzbekistan on Statistics. Uzbekistan Multiple Indicator Cluster Survey 2006. New York, United States: United Nations Children's Fund (UNICEF). | GHDx |
| **13719** | Vietnam | 0 | 8 | 1760 | Vietnam Multiple Indicator Cluster Survey 2006 | 2006 | General Statistics Office (Vietnam), United Nations Children's Fund (UNICEF). Vietnam Multiple Indicator Cluster Survey 2006. New York, United States of America: United Nations Children's Fund (UNICEF). | GHDx |
| **21058** | Vietnam | 0 | 41 | 982 | Vietnam Demographic and Health Survey 2002 | 2002 | General Statistics Office (Vietnam), Macro International, Inc. Vietnam Demographic and Health Survey 2002. Fairfax, United States of America: ICF International. | GHDx |

| | | | | | | | | |
|---|---|---|---|---|---|---|---|---|
| **57999** | Vietnam | 0 | 568 | 2262 | Vietnam Multiple Indicator Cluster Survey 2010-2011 | 2010-2011 | General Statistics Office (Vietnam), United Nations Children's Fund (UNICEF). Vietnam Multiple Indicator Cluster Survey 2010-2011. New York, United States of America: United Nations Children's Fund (UNICEF). | GHDx |
| **152735** | Vietnam | 0 | 476 | 1497 | Vietnam | 2013-2014 | General Statistics Office (Vietnam), United Nations Children's Fund (UNICEF). Vietnam Multiple Indicator Cluster Survey 2013-2014. New York, United States of America: United Nations Children's Fund (UNICEF), 2015. | GHDx |
| **13795** | Yemen | 0 | 418 | 2159 | Yemen Family Health Survey 2003 | 2003 | Central Statistical Organization (Yemen), League of Arab States, Ministry of Public Health and Population (Yemen), Pan Arab Project for Family Health (PAPFAM). Yemen Family Health Survey 2003. | GHDx |
| **13816** | Yemen | 0 | 21 | 2169 | Yemen Multiple Indicator Cluster Survey 2006 | 2006 | Ministry of Health (Yemen) and United Nations Children's Fund (UNICEF). Yemen Multiple Indicator Cluster Survey 2006. New York, United States: United Nations Children's Fund (UNICEF). | GHDx |
| **112500** | Yemen | 0 | 528 | 9114 | Yemen Demographic and Health Survey 2013 | 2013 | Central Statistical Organization (Yemen), ICF International, Ministry of Public Health and Population (Yemen). Yemen Demographic and Health Survey 2013. Fairfax, United States of America: ICF International. | GHDx |
| **14027** | Zambia | 0 | 72 | 3132 | Zambia Living Conditions Monitoring Survey 2002-2003 | 2002-2003 | Central Statistical Office (Zambia). Zambia Living Conditions Monitoring Survey 2002-2003. Lusaka, Zambia: Central Statistical Office (Zambia). | GHDx |
| **21102** | Zambia | 0 | 72 | 1892 | Zambia Demographic and Health Survey 2001-2002 | 2001-2002 | Central Board of Health (Zambia), Central Statistical Office (Zambia), Macro International, Inc. Zambia Demographic and Health Survey 2001-2002. Fairfax, United States of America: ICF International. | GHDx |
| **21117** | Zambia | 319 | 0 | 5302 | Zambia Demographic | 2007 | Central Statistical Office (Zambia), Macro International, Inc. Zambia Demographic and Health Survey 2007. Fairfax, United States of America: ICF International. | GHDx |

| | | | | | | | | |
|---|---|---|---|---|---|---|---|---|
| | | | | | and Health Survey 2007 | | | |
| **26702** | Zambia | 0 | 22 | 2237 | Zambia Global Fund Household Health Coverage Survey 2008 | 2008 | Central Statistical Office (Zambia). Zambia Global Fund Household Health Coverage Survey 2008. Lusaka, Zambia: Central Statistical Office (Zambia). | GHDx |
| **77516** | Zambia | 719 | 0 | 11048 | Zambia Demographic and Health Survey 2013-2014 | 2013-2014 | Central Statistical Office (Zambia), ICF International, Ministry of Health (Zambia), Tropical Diseases Research Centre, University Teaching Hospital (Zambia), University of Zambia. Zambia Demographic and Health Survey 2013-2014. Fairfax, United States of America: ICF International. | GHDx |
| **21163** | Zimbabwe | 394 | 0 | 2811 | Zimbabwe Demographic and Health Survey 2005-2006 | 2005-2006 | Central Statistical Office (Zimbabwe), Macro International, Inc. Zimbabwe Demographic and Health Survey 2005-2006. Fairfax, United States of America: ICF International. | GHDx |
| **35493** | Zimbabwe | 0 | 10 | 1916 | Zimbabwe Multiple Indicator Monitoring Survey 2009 | 2009 | Central Statistical Office (Zimbabwe). Zimbabwe Multiple Indicator Monitoring Survey 2009. New York, United States of America: United Nations Children's Fund (UNICEF). | GHDx |
| **55992** | Zimbabwe | 391 | 0 | 3824 | Zimbabwe Demographic and Health Survey 2010-2011 | 2010-2011 | ICF Macro, Zimbabwe National Statistics Agency. Zimbabwe Demographic and Health Survey 2010-2011. Calverton, United States of America: ICF Macro, 2012. | GHDx |
| **152720** | Zimbabwe | 0 | 10 | 8033 | Zimbabwe Multiple Indicator Cluster Survey 2014 | 2014 | United Nations Children's Fund (UNICEF), Zimbabwe National Statistics Agency. Zimbabwe Multiple Indicator Cluster Survey 2014. New York, United States of America: United Nations Children's Fund (UNICEF), 2015. | GHDx |
| **157066** | Zimbabwe | 398 | 0 | 3031 | Zimbabwe Demographic | 2015 | ICF International, National Microbiology Reference Laboratory, Harare Central Hospital (NMRL) (Zimbabwe), Zimbabwe National Statistics Agency. Zimbabwe | GHDx |

| | | | | | | | | |
|---|---|---|---|---|---|---|---|---|
| | | | | | and Health Survey 2015 | | Demographic and Health Survey 2015. Fairfax, United States of America: ICF International, 2016. | |
| **431951** | Zimbabwe | 0 | 10 | 2381 | Zimbabwe Multiple Indicator Cluster Survey 2019 | 2019 | United Nations Children's Fund (UNICEF), Zimbabwe National Statistics Agency. Zimbabwe Multiple Indicator Cluster Survey 2019. New York, United States of America: United Nations Children's Fund (UNICEF), 2020. | GHDx |

*Supplementary Table 5:* Survey sources excluded from analysis
Citations, series, country, and years provided for each excluded data set along with rationale for their exclusion. This table includes results of all LMICs from the GHDx, whether they were included in final analysis or not.

| Country | Series | Year(s) | Citation | Rationale for exclusion |
|---|---|---|---|---|
| **Albania** | Albania Multiple Indicator Cluster Survey 2005 | 2005 | National Institute of Statistics (Albania), United Nations Children's Fund (UNICEF). Albania Multiple Indicator Cluster Survey 2005. New York, United States: United Nations Children's Fund (UNICEF). | Estimates considered implausible. Survey estimates are systematically low compared to admin estimates and estimates from other established series (2004-2006 DHS) |
| **Albania** | Albania Demographic and Health Survey 2017-2018 | 2017-2018 | Albania Institute of Public Health (IPH), Albania Institute of Statistics (INSTAT), ICF International. Albania Demographic and Health Survey 2017-2018. Fairfax, United States of America: ICF International, 2019. | Survey outliered due to absence of required survey variable indicators and reliability concerns. |
| **Angola** | Angola Core Welfare Indicators Questionnaire Survey 2011 | 2011 | Ministry of Planning and Territorial Development (Angola), National Institute of Statistics (Angola). Angola Core Welfare Indicators Questionnaire Survey 2011. Luanda, Angola: National Institute of Statistics (Angola). | Estimates considered implausible. Survey estimates are systematically low compared to admin estimates |
| **Armenia** | Armenia Demographic and Health Survey 2005 | 2005 | MOH Center for Health Information and Statistics, Macro International, Inc, National Statistical Service (NSS). Armenia Demographic and Health Survey 2005. Fairfax, United States of America: ICF International. | Outliered due to small sample size and reliability concerns. Estimates considered implausibly low compared to admin estimates |
| **Bangladesh** | Bangladesh - Dhaka Malnutrition and Enteric Disease Study 2009-2014 | 2009-2014 | Fogarty International Center, National Institutes of Health (NIH), Foundation for the National Institutes of Health (FNIH), International Centre for Diarrhoeal Disease Research, Bangladesh (ICDDR,B). Bangladesh - Dhaka Malnutrition and Enteric Disease Study 2009-2014. | Survey estimates include implausibly high coverage rates of 100% |
| **Bangladesh** | Bangladesh GAVI FCE Measles-Rubella Post-Vaccine | 2014 | Institute for Health Metrics and Evaluation (IHME), International Centre for Diarrhoeal Disease Research, Bangladesh (ICDDR,B). Bangladesh GAVI FCE Measles-Rubella Post- | Post-campaign surveys make it difficult to distinguish routine immunization coverage from campaign coverage. |

| | | | | |
|---|---|---|---|---|
| | Campaign Household Survey 2014 | | Vaccine Campaign Household Survey 2014. Seattle, United States of America: Institute for Health Metrics and Evaluation (IHME), 2017. | |
| **Benin** | Benin Demographic and Health Survey 2011-2012 | 2011-2012 | ICF International, National Institute of Statistics and Economic Analysis (INSAE) (Benin), National Program Against AIDS (PNLS) (Benin). Benin Demographic and Health Survey 2011-2012. Fairfax, United States: ICF International, 2014. | Estimates considered implausible. Survey estimates are systematically low compared to admin estimates and estimates from other established survey series (2009-2012 Benin Health Statistical Yearbook, 2008 External Review of the Extended Programme on Immunisation) |
| **Burkina Faso** | Burkina Faso Core Welfare Indicators Questionnaire Survey 2005 | 2005 | National Institute of Statistics and Demography (Burkina Faso), World Bank. Burkina Faso Core Welfare Indicators Questionnaire Survey 2005. Ouagadougou, Burkina Faso: National Institute of Statistics and Demography (Burkina Faso). | Estimates considered implausible. Survey estimates are inconsistent with admin estimates and estimates from other established survey series (Burkina Faso Multiple Indicator Cluster Survey 2006, Burkina Faso Demographic and Health Survey 2003) |
| **Burkina Faso** | Burkina Faso Core Welfare Indicators Questionnaire Survey 2007 | 2007 | National Institute of Statistics and Demography (INSD). Burkina Faso Core Welfare Indicators Questionnaire Survey 2007. Ouagadougou, Burkina Faso: National Institute of Statistics and Demography (INSD), 2008. | Estimates considered implausible. Survey estimates are inconsistent with admin estimates and estimates from other established survey series (Burkina Faso Multiple Indicator Cluster Survey 2006, Burkina Faso Demographic and Health Survey 2003) |
| **Cambodia** | Cambodia Socio-Economic Survey 2009 | 2009 | National Institute of Statistics (Cambodia), Statistics Sweden. Cambodia Socio-Economic Survey 2009. Phnom Penh, Cambodia: National Institute of Statistics (Cambodia). | Estimates considered implausible. Survey estimates are inconsistent with admin estimates and estimates from other established survey series (Cambodia Demographic and Health Survey 2005-2006, Cambodia Demographic and Health Survey 2010-2011) |
| **Congo** | Congo Demographic and Health Survey 2005 | 2005 | Macro International, Inc, National Center for Statistics and Economic Studies (Congo, Rep.). Congo Demographic and Health Survey 2005. Fairfax, United States of America: ICF International. | Cohort years 2000-2003 removed due to implausible coverage rates. |

| | | | | |
|---|---|---|---|---|
| **Costa Rica** | Costa Rica National Health Survey 2006 | 2006 | Central American Population Center, University of Costa Rica. Costa Rica National Health Survey 2006. San José, Costa Rica: Central American Population Center, University of Costa Rica. | Estimates considered implausible. Survey estimates are systematically low compared to admin estimates and estimates from other established survey series (2007-2008 MICS) |
| **Djibouti** | Djibouti Family Health Survey 2002 | 2002 | Department of Statistics and Demographic Studies (Djibouti), League of Arab States, Ministry of Health (Djibouti), Pan Arab Project for Family Health (PAPFAM). Djibouti Family Health Survey 2002. | Large difference in cohort coverage, with older cohorts having implausibly high coverage compare to younger cohorts. |
| **Ecuador** | Ecuador Reproductive Health Survey 2004 | 2004 | Center for Studies of Population and Social Development (CEPAR) (Ecuador) and Division of Reproductive Health-Centers for Disease Control and Prevention (CDC). (2005) Ecuador Reproductive Health Survey 2004. Quito, Ecuador: CEPAR. | Estimates considered implausible. Survey reports an anomalous drop in coverage in 2004 that is inconsistent with admin estimates and estimates from other established survey series (2005-2006 Ecuador Living Conditions Survey) |
| **El Salvador** | El Salvador Reproductive Health Survey 2002-2003 | 2002-2003 | Asociación demográfica salvadoreña (ADS), Division of Reproductive Health-Centers for Disease Control and Prevention (CDC). (2004) El Salvador Reproductive Health Survey 2002-2003. San Salvador, El Salvador: ADS. | Survey estimates include implausibly high coverage rates of 100% |
| **El Salvador** | El Salvador Reproductive Health Survey 2008 | 2008 | Asociación demográfica salvadoreña (ADS), Division of Reproductive Health-Centers for Disease Control and Prevention (CDC). (2009) El Salvador Reproductive Health Survey 2008. San Salvador, El Salvador: ADS. | Estimates considered implausible. Survey estimates are systematically low compared to admin estimates and estimates from other established survey series (2012-2013 MICS) |
| **Ethiopia** | Ethiopia Welfare Monitoring Survey 2004 | 2004 | Central Statistical Agency (Ethiopia). Ethiopia Welfare Monitoring Survey 2004. | Estimates considered implausible. Survey estimates are systematically high compared to admin estimates and estimates from other established surveys series (2001-2003 DHS, 2001 National EPI Coverage Survey) |
| **Georgia** | Georgia Multiple Indicator Cluster Survey 2005 | 2005 | National Center for Disease Control (Georgia), State Department of Statistics of Georgia, United Nations Children's Fund (UNICEF). Georgia Multiple Indicator Cluster Survey 2005. New | Estimates considered implausible. Survey estimates are systematically low compared to admin estimates |

| | | | | |
|---|---|---|---|---|
| | | | York, United States: United Nations Children's Fund (UNICEF). | |
| **Ghana** | Ghana Living Standards Measurement Survey 2012-2013 | 2012-2013 | Ghana Statistical Service, World Bank. Ghana Living Standards Measurement Survey 2012-2013. Accra, Ghana: Ghana Statistical Service. | Estimates considered implausible. Survey estimates are systematically low compared to admin estimates |
| **Guatemala** | Guatemala Demographic and Health Survey 2014-2015 | 2014-2015 | ICF International, Institute of Nutrition of Central America and Panama, Ministry of Public Health and Social Assistance (Guatemala), National Statistics Institute (Guatemala), Secretary of Planning and Programming of the Presidency (Segeplan) (Guatemala). Guatemala Demographic and Health Survey 2014-2015. Fairfax, United States: ICF International, 2017. | Estimates considered implausible. Survey estimates are systematically high compared to admin estimates and trends from other established surveys in nearby years (2008-2009 Guatemala Reproductive Health Survey) |
| **Honduras** | Honduras Survey of Living Conditions 2004 | 2004 | National Institute of Statistics (Honduras). Honduras Survey of Living Conditions 2004. Tegucigalpa, Honduras: National Institute of Statistics (Honduras). | Survey estimates include implausibly high coverage rates of 100% |
| **India** | India District Level Household Survey 2007-2008 | 2007-2008 | International Institute for Population Sciences (India). India District Level Household Survey 2007-2008. Mumbai, India: International Institute for Population Sciences (India), 2010. | Estimates considered implausible. Survey estimates are systematically high compared to admin estimates and estimates from other established survey series (2003-2004 DHS, 2004-2005 India Human Development Survey, 2005 India Coverage Evaluation Survey) |
| **India** | India - Vellore Malnutrition and Enteric Disease Study 2009-2014 | 2009-2014 | Fogarty International Center, National Institutes of Health (NIH), Foundation for the National Institutes of Health (FNIH). India - Vellore Malnutrition and Enteric Disease Study 2009-2014. | 2010 estimates considered implausibly low compared to other survey years. |
| **India** | India District Level Household | 2012-2014 | International Institute for Population Sciences (India). India District Level Household Survey 2012-2014. New Delhi, India: Ministry of Health and Family Welfare (India). | Estimates considered implausible. Coverage estimates are greater than 12% higher than coverage reported by India Demographic and |

| | | | | |
|---|---|---|---|---|
| | Survey 2012-2014 | | | Health Survey 2015-2016 for the same cohort years. |
| **Indonesia** | Indonesia Family Life Survey 2000 | 2000 | Center for Population and Policy Studies, Gadjah Mada University (Indonesia), RAND Corporation. Indonesia Family Life Survey 2000. Santa Monica, United States: RAND Corporation. | Estimates considered implausible. Survey estimates are systematically high compared to admin estimates and estimates from other established survey series (1996-1999 DHS) |
| **Indonesia** | Indonesia National Socioeconomic Survey 2006 | 2004-2013 | Statistics Indonesia. Indonesia National Socioeconomic Survey 2006. | Estimates considered implausible. Survey estimates are inconsistent with admin estimates and estimates from other established survey series (2007-2008 Indonesia Family Life Survey, 2003-2005 DHS) |
| **Indonesia** | Indonesia Family Life Survey East 2012 | 2012 | National Team for the Acceleration of Poverty Reduction (TNP2K) (Indonesia), SurveyMETER, University of Southern California, World Bank. Indonesia Family Life Survey East 2012. | Estimates considered implausible. Survey estimates are inconsistent with admin estimates and estimates from other established survey series (2008-2011 DHS) |
| **Kenya** | Kenya - North Eastern Province Multiple Indicator Cluster Survey 2007 | 2007 | Kenya National Bureau of Statistics, United Nations Children's Fund (UNICEF). Kenya - North Eastern Province Multiple Indicator Cluster Survey 2007. Nairobi, Kenya: Kenya National Bureau of Statistics. | Estimates considered implausible. Survey estimates are systematically low compared to admin estimates and estimates from other established survey series (2005-2006 Kenya Integrated Household Budget Survey, 2004-2006 DHS) |
| **Kyrgyzstan** | Kyrgyzstan Multiple Indicator Cluster Survey 2014 | 2014 | National Statistical Committee of the Kyrgyz Republic, United Nations Children's Fund (UNICEF). Kyrgyzstan Multiple Indicator Cluster Survey 2014. New York, United States of America: United Nations Children's Fund (UNICEF), 2015. | Implausibly low coverage estimates of 0% for some age cohorts. |
| **Lebanon** | Lebanon Family Health Survey 2004 | 2004 | Central Administration of Statistics (Lebanon), League of Arab States, Ministry of Social Affairs (Lebanon), Pan Arab Project for Family Health (PAPFAM). Lebanon Family Health Survey 2004. | Data was only collected at national level |

| | | | | |
|---|---|---|---|---|
| **Lebanon** | Palestinians in Lebanon Multiple Indicator Cluster Survey 2005-2006 | 2005-2006 | Palestinian Central Bureau of Statistics, Pan Arab Project for Family Health (PAPFAM), United Nations Children's Fund (UNICEF). Palestinians in Lebanon Multiple Indicator Cluster Survey 2005-2006. New York, United States: United Nations Children's Fund (UNICEF). | Estimates considered implausible. Survey estimates are inconsistent with both admin estimates and estimates from other established survey series (2006-2007 Palestine Family Health Survey) |
| **Lebanon** | Palestinians in Lebanon Multiple Indicator Cluster Survey 2011 | 2011 | Palestinian Central Bureau of Statistics, United Nations Children's Fund (UNICEF). Palestinians in Lebanon Multiple Indicator Cluster Survey 2011. New York, United States: United Nations Children's Fund (UNICEF), 2013. | Estimates considered implausible. Survey estimates are inconsistent with both admin estimates and estimates from other established survey series (2004 Lebanon Family Health Survey) |
| **Moldova** | Moldova Multiple Indicator Cluster Survey 2000 | 2000 | National Scientific and Applied Center for Preventive Medicine (NCPM) (Moldova), United Nations Children's Fund (UNICEF). Moldova Multiple Indicator Cluster Survey 2000. New York, United States: United Nations Children's Fund (UNICEF). | Estimates considered implausible. Survey estimates are systematically high compared to admin estimates and estimates from other established survey series (2002-2004 DHS) |
| **Mali** | Mali Multiple Indicator Cluster Survey 2009-2010 | 2009-2010 | Ministry of Health (Mali), National Institute of Statistics (INSTAT) (Mali), United Nations Children's Fund (UNICEF). Mali Multiple Indicator Cluster Survey 2009-2010. New York, United States: United Nations Children's Fund (UNICEF), 2017. | Estimates considered implausible. Survey estimates are inconsistent with both admin estimates and estimates from other established survey series (2008-2011 DHS, 2010-2013 World Bank Living Standards Measurement Survey) |
| **Mexico** | Mexico Family Life Survey 2005-2006 | 2005-2006 | California Center for Population Research (CCPR), University of California Los Angeles (UCLA), Center for Research and Teaching in Economics (CIDE) (Mexico), Ibero-American University, National Institute of Public Health (Mexico). Mexico Family Life Survey 2005-2006. | Estimates considered implausible. Survey estimates are inconsistent with both admin estimates and estimates from other established survey series (2008-2009 Mexican National Health and Nutrition Survey) |
| **Mexico** | Mexico Family Life Survey 2008-2013 | 2008-2013 | Center for Research and Teaching in Economics (CIDE) (Mexico), Duke University, Ibero-American University, National Institute of Public Health (Mexico), University of | Estimates considered implausible. Sudden drop in coverage inconsistent with admin estimates and estimates from other established survey series (Mexico National Survey of Health and Nutrition |

| | | | | |
|---|---|---|---|---|
| | | | California, Los Angeles (UCLA). Mexico Family Life Survey 2008-2013. | 2011-2012, Mexico Multiple Indicator Cluster Survey 2015) |
| **Mongolia** | Mongolia Multiple Indicator Cluster Survey 2013 | 2013 | Government of Mongolia, National Statistical Office of Mongolia, United Nations Children's Fund (UNICEF), United Nations Population Fund (UNFPA). Mongolia Multiple Indicator Cluster Survey 2013. New York, United States: United Nations Children's Fund (UNICEF), 2016. | Estimates considered implausible. Sudden drop in coverage inconsistent with admin estimates and MICS estimates from neighboring years |
| **Morocco** | Morocco National Survey on Population and Family Health 2010-2011 | 2010-2011 | Ministry of Health (Morocco), Pan Arab Project for Family Health (PAPFAM), United Nations Children's Fund (UNICEF), United Nations Population Fund (UNFPA), World Health Organization (WHO). Morocco National Survey on Population and Family Health 2010-2011. | Estimates for cohort year 2008 removed due to 100% coverage. Other cohort years retained |
| **Nepal** | Nepal Living Standards Measurement Survey 2003-2004 | 2009-2014 | Fogarty International Center, National Institutes of Health (NIH), Foundation for the National Institutes of Health (FNIH), Institute of Medicine, Tribhuvan University, University of Bergen, Walter Reed/AFRIMS Research Unit Nepal (WARUN). Nepal - Bhaktapur Malnutrition and Enteric Disease Study 2009-2014. | Estimates considered implausible. Survey estimates include implausibly high coverage rates of 100% |
| **Nepal** | Nepal - Bhaktapur Malnutrition and Enteric Disease Study 2009-2014 | 2014-2015 | National Institute of Statistics (Niger), World Bank. Niger National Survey on Household Living Conditions and Agriculture 2014-2015. Washington DC, United States of America: World Bank. | Estimates considered implausible. Survey estimates include implausibly high coverage rates of 100% |
| **Niger** | Niger National Survey on Household Living Conditions and Agriculture 2014-2015 | 2014-2015 | National Institute of Statistics (Niger), World Bank. Niger National Survey on Household Living Conditions and Agriculture 2014-2015. Washington DC, United States of America: World Bank. | Estimates outliered due to reliability concerns, survey design, and implausibly low coverage. |

| | | | | |
|---|---|---|---|---|
| **Nigeria** | Nigeria Reproductive Health, Child Health, and Education Household, School, and Health Facility Baseline Surveys 2005 | 2005 | MEASURE Evaluation Project, Carolina Population Center, University of North Carolina, Center for Research, Evaluation, and Resource Development (CRERD), Center for Communication Programs, Bloomberg School of Public Health, Johns Hopkins, Creative Associates International, Constella Futures, Adolescent Health and Information Project (Nigeria), Federation of Muslim Women's Associations of Nigeria (FOMWAN), Nigerian Medical Association, Management Sciences for Health (MSH), Civil Society Action Coalition on Education For All. Nigeria Reproductive Health, Child Health, and Education Household, School, and Health Facility Baseline Surveys 2005. Chapel Hill, United States: MEASURE Evaluation Project, Carolina Population Center, University of North Carolina. | Estimates considered implausible. Survey estimates are inconsistent with both admin estimates and estimates from other established survey series (1999-2007 DHS, 2007-2010 MICS) |
| **Nigeria** | Nigeria General Household Survey 2007 | 2007 | Central Bank of Nigeria, National Bureau of Statistics (Nigeria), Nigerian Communications Commission (NCC). Nigeria General Household Survey 2007. Abuja, Nigeria: National Bureau of Statistics (Nigeria). | Estimates considered implausible. Survey estimates are systematically low compared to admin estimates and estimates from other established survey series (1999-2007 DHS, 2007-2010 MICS) |
| **Nigeria** | Nigeria Reproductive Health, Child Health, and Education Household, School, and Health Facility Midline Surveys 2007 | 2007 | MEASURE Evaluation Project, Carolina Population Center, University of North Carolina, Center for Research, Evaluation, and Resource Development (CRERD), Center for Communication Programs, Bloomberg School of Public Health, Johns Hopkins, Creative Associates International, Constella Futures, Adolescent Health and Information Project (Nigeria), Federation of Muslim Women's Associations of Nigeria (FOMWAN), Nigerian Medical Association, Management Sciences for Health (MSH), Civil Society Action Coalition on Education For All. Nigeria Reproductive Health, | Estimates considered implausible. Survey estimates are inconsistent with both admin estimates and estimates from other established survey series (1999-2007 DHS, 2007-2010 MICS) |

| | | | | |
|---|---|---|---|---|
| | | | Child Health, and Education Household, School, and Health Facility Midline Surveys 2007. Chapel Hill, United States: MEASURE Evaluation Project, Carolina Population Center, University of North Carolina. | |
| **Nigeria** | Nigeria Living Standards Survey 2008-2010 | 2008-2010 | National Bureau of Statistics (Nigeria). Nigeria Living Standards Survey 2008-2010. Abuja, Nigeria: National Bureau of Statistics (Nigeria). | Estimates considered implausible. Survey estimates are systematically high compared to admin estimates and estimates from other established survey series (1999-2007 DHS, 2007-2010 MICS) |
| **Nigeria** | Nigeria General Household Survey 2010-2011 | 2010-2011 | National Bureau of Statistics (Nigeria). Nigeria General Household Survey 2010-2011. Abuja, Nigeria: National Bureau of Statistics (Nigeria). | Estimates considered implausible. Survey estimates are systematically high compared to admin estimates and estimates from other established survey series (2009 DHS, 2009 MICS, 2009 Nigeria Reproductive Health, Child Health, and Education Household, School, and Health Facility Survey, 2009-2012 Nigeria Annual Abstract of Statistics) |
| **Nigeria** | Nigeria General Household Survey 2012-2013 | 2012-2013 | National Bureau of Statistics (Nigeria). Nigeria General Household Survey 2012-2013. Washington DC, United States: World Bank. | Estimates considered implausible. Survey estimates are systematically low compared to admin estimates and estimates from other established survey series (2011 DHS) |
| **Nigeria** | Nigeria Multiple Indicator Cluster Survey with National Immunization Coverage Survey Supplement 2016-2017 | 2016-2017 | National Agency for the Control of AIDS (Nigeria), National Bureau of Statistics (Nigeria), National Primary Health Care Development Agency (NPHCDA) (Nigeria), United Nations Children's Fund (UNICEF). Nigeria Multiple Indicator Cluster Survey with National Immunization Coverage Survey Supplement 2016-2017. New York, United States: United Nations Children's Fund (UNICEF), 2018. | Estimates considered implausible. Survey estimates are systematically high compared to admin estimates and estimates from other established survey series (1999-2007 DHS) |
| **Pakistan** | Pakistan Social and Living Standards Measurement | 2014-2015 | Pakistan Bureau of Statistics. Pakistan Social and Living Standards Measurement Survey 2014-2015. Islamabad, Pakistan: Pakistan Bureau of Statistics, 2016. | Estimates considered implausible. Survey estimates are inconsistent with admin estimates and estimates from other established survey series |

| | | | | |
|---|---|---|---|---|
| | Survey 2014-2015 | | | (2010-2011 DHS, 2011 Pakistan National Nutrition Survey) |
| **Peru** | Peru Continuous Demographic and Health Survey 2003-2008 | 2003-2008 | Ministry of Economy and Finance (Peru), National Institute of Statistics and Informatics (Peru), ORC Macro. Peru Continuous Demographic and Health Survey 2003-2008. Fairfax, United States of America: ICF International. | Survey outliered due to absence of required survey variable indicators. |
| **Peru** | Peru - Loreto Malnutrition and Enteric Disease Study 2009-2014 | 2009-2014 | Fogarty International Center, National Institutes of Health (NIH), Foundation for the National Institutes of Health (FNIH), Johns Hopkins Bloomberg School of Public Health. Peru - Loreto Malnutrition and Enteric Disease Study 2009-2014. | Estimates considered implausible. Survey estimates include implausibly high coverage rates of 100% |
| **Qatar** | Qatar Multiple Indicator Cluster Survey 2012 | 2012 | Ministry of Development Planning and Statistics (Qatar), Qatar Foundation for Education, Science and Community Development, Qatar Statistics Authority, Supreme Council of Health (Qatar), United Nations Children's Fund (UNICEF). Qatar Multiple Indicator Cluster Survey 2012. New York, United States: United Nations Children's Fund (UNICEF), 2018. | Estimates considered implausible. Survey estimates are systematically low |
| **Sierra Leone** | Sierra Leone Multiple Indicator Cluster Survey 2010 | 2010 | Statistics Sierra Leone, United Nations Children's Fund (UNICEF). Sierra Leone Multiple Indicator Cluster Survey 2010. New York, United States: United Nations Children's Fund (UNICEF). | Estimates considered implausible. Survey estimates are systematically high compared to admin estimates and estimates from other established surveys over the same period (2006-2009 DHS) |
| **Uganda** | Uganda National Service Delivery Survey 2004 | 2004 | Ministry of Public Service (Uganda), Uganda Bureau of Statistics. Uganda National Service Delivery Survey 2004. OpenMicroData. | Estimates considered implausible. Survey estimates are systematically high compared to estimates from other established surveys over the same period (Uganda Demographic and Health Survey 2000-2001, Uganda Demographic and Health Survey 2011). Also, some households were not covered due to insecurity in the districts of Gulu, Lira, Katakwi, Kitgum, and Pader; while in Karamoja region (Kotido, Nakapiripirit and |

| | | | | |
|---|---|---|---|---|
| | | | | Moroto) due to mobility of the pastoralist communities. |
| **Uganda** | Uganda Gavi FCE Household Survey 2015 | 2015 | Institute for Health Metrics and Evaluation (IHME), Infectious Diseases Research Collaboration (IDRC). Uganda Gavi FCE Household Survey 2015. Seattle, United States: Institute for Health Metrics and Evaluation (IHME), 2017. | Estimates considered implausible. Survey estimates are inconsistent with admin estimates and estimates from other established survey series (Uganda Living Standards Measurement Survey - Integrated Survey on Agriculture 2013-2014, Uganda Demographic and Health Survey 2016) |
| **Yemen** | Yemen - Dhamar Nutritional Status and Mortality Survey 2013 | 2013 | Ministry of Public Health and Population (Yemen), Yemen UNICEF. Yemen - Dhamar Nutritional Status and Mortality Survey 2013. | Estimates considered implausible. Survey estimates include implausibly high coverage rates of 100% |

*Supplementary Table 6:* Geospatial covariates used in modelling
For each covariate used in modelling, temporal resolutions and references are provided below.

| **Covariate** | **Temporal resolutio n** | **Source** | **Reference** | **NID** |
|---|---|---|---|---|
| **Travel time to nearest settlement** | Static | Oxford | Weiss, D. J., et al. A global map of travel time to cities to assess inequalities in accessibility in 2015. Nature 533, 333-336 (2018). | 417828 |
| **Distance from rivers or lakes** | Static | Natural Earth Data (derived) | Available at: http://www.naturalearthdata.com/downloads/10m-physical-vectors/10m-rivers-lake-centerlines/ AND http://www.worldwildlife.org/pages/global-lakes-and-wetlands-database | 418521 and 418284 |
| **Elevation** | Static | NOAA GLOBE | Available at: https://www.ngdc.noaa.gov/mgg/topo/gltiles.html | 238858 |
| **Population** | Annual | WorldPop | Lloyd, C. T., et al. Global spatio-temporally harmonised datasets for producing high-resolution gridded population distribution datasets. Big Earth Data. 2096(4471): 2574-5417 (2019).<br><br>World Pop. Get data. Available at: http://www.worldpop.org.uk/data/get_data/. (Accessed: 22th January 2019) | 420764 |
| **Urban or rural** | Annual | European Commission / GHS | Pesaresi, M. et al. Operating procedure for the production of the Global Human Settlement Layer from Landsat data of the epochs 1975, 1990, 2000, and 2014. (Publications Office of the European Union, 2016). Available at: http://ghsl.jrc.ec.europa.eu/data.php | 418851 |
| **Irrigation** | Static | University of Frankfurt and FAO | Siebert, S., Doll, P., Hoogeveen, J., Faures, J.-M., Frenken, K., & Feick, S. Development and validation of the global map of irrigation areas. Hydrology and Earth System Sciences 9, 535-547 (2005).<br><br>Goethe-Universität. Generation of a digital global map of irrigation areas. Available at: https://www.unifrankfurt.de/45218039/Global_Irrigation_Map. (Accessed: 25th July 2017). Also from: http://www.fao.org/nr/water/aquastat/irrigationmap/index10.stm | 419086 |
| **Cloud cover percentage** | Annual | Climatic Research Unit | Harris, I., Osborn, T.J., Jones, P. et al. Version 4 of the CRU TS monthly high-resolution gridded multivariate climate dataset. Sci Data 7, 109 (2020). https://doi.org/10.1038/s41597-020-0453-3 | 450685 |

| | | | | |
|---|---|---|---|---|
| | | | High-resolution gridded datasets (and derived products). Available at: https://crudata.uea.ac.uk/cru/data/hrg/. | |
| **Mean diurnal temperature range** | Annual | Climatic Research Unit | Harris, I., Osborn, T.J., Jones, P. et al. Version 4 of the CRU TS monthly high-resolution gridded multivariate climate dataset. Sci Data 7, 109 (2020). https://doi.org/10.1038/s41597-020-0453-3<br><br>High-resolution gridded datasets (and derived products). Available at: https://crudata.uea.ac.uk/cru/data/hrg/. | 450685 |
| **Frost day frequency** | Annual | Climatic Research Unit | Harris, I., Osborn, T.J., Jones, P. et al. Version 4 of the CRU TS monthly high-resolution gridded multivariate climate dataset. Sci Data 7, 109 (2020). https://doi.org/10.1038/s41597-020-0453-3<br><br>High-resolution gridded datasets (and derived products). Available at: https://crudata.uea.ac.uk/cru/data/hrg/. | 450685 |
| **Mean potential evapotranspiration per day per month** | Annual | Climatic Research Unit | Harris, I., Osborn, T.J., Jones, P. et al. Version 4 of the CRU TS monthly high-resolution gridded multivariate climate dataset. Sci Data 7, 109 (2020). https://doi.org/10.1038/s41597-020-0453-3<br><br>High-resolution gridded datasets (and derived products). Available at: https://crudata.uea.ac.uk/cru/data/hrg/. | 450685 |
| **Average daily minimum temperature** | Annual | Climatic Research Unit | Harris, I., Osborn, T.J., Jones, P. et al. Version 4 of the CRU TS monthly high-resolution gridded multivariate climate dataset. Sci Data 7, 109 (2020). https://doi.org/10.1038/s41597-020-0453-3<br><br>High-resolution gridded datasets (and derived products). Available at: https://crudata.uea.ac.uk/cru/data/hrg/. | 450685 |
| **Average daily mean temperature** | Annual | Climatic Research Unit | Harris, I., Osborn, T.J., Jones, P. et al. Version 4 of the CRU TS monthly high-resolution gridded multivariate climate dataset. Sci Data 7, 109 (2020). https://doi.org/10.1038/s41597-020-0453-3<br><br>High-resolution gridded datasets (and derived products). Available at: https://crudata.uea.ac.uk/cru/data/hrg/. | 450685 |
| **Average daily maximum temperature** | Annual | Climatic Research Unit | Harris, I., Osborn, T.J., Jones, P. et al. Version 4 of the CRU TS monthly high-resolution gridded multivariate climate dataset. Sci Data 7, 109 (2020). https://doi.org/10.1038/s41597-020-0453-3 | 450685 |

| | | | | |
|---|---|---|---|---|
| | | | High-resolution gridded datasets (and derived products). Available at: https://crudata.uea.ac.uk/cru/data/hrg/. | |
| **Mean vapour pressure** | Annual | Climatic Research Unit | Harris, I., Osborn, T.J., Jones, P. et al. Version 4 of the CRU TS monthly high-resolution gridded multivariate climate dataset. Sci Data 7, 109 (2020). https://doi.org/10.1038/s41597-020-0453-3<br><br>High-resolution gridded datasets (and derived products). Available at: https://crudata.uea.ac.uk/cru/data/hrg/. | 450685 |
| **Monthly wet day frequency** | Annual | Climatic Research Unit | Harris, I., Osborn, T.J., Jones, P. et al. Version 4 of the CRU TS monthly high-resolution gridded multivariate climate dataset. Sci Data 7, 109 (2020). https://doi.org/10.1038/s41597-020-0453-3<br><br>High-resolution gridded datasets (and derived products). Available at: https://crudata.uea.ac.uk/cru/data/hrg/. | 450685 |
| **Enhanced vegetation index** | Annual | Moderate Resolution Imaging Spectroradiometer (MODIS) | Weiss, D. J. et al. An effective approach for gapfilling continental scale remotely sensed timeseries. Isprs J. Photogramm. Remote Sens. 98, 106–118 (2014).<br><br>USGS & NASA. Vegetation indices 16-Day L3 global 500m MOD13A1 dataset. Available at: https://lpdaac.usgs.gov/products/mcd12q1v006/. (2020) | 422343 |
| **Growing season length** | Static | FAO Global Agro-Ecological Zone (GAEZ) project | Available at: http://www.fao.org/nr/gaez/about-data-portal/en/ | 419508 |
| **Normalised difference vegetation index** | Annual | MODIS | NASA & NOAA. Advanced Very High Resolution Radiometer (AVHRR) Normalized Difference Vegetation Index (NDVI) dataset. Available at: https://www.nasa.gov/nex. (Accessed: 25th July 2017) | 419645 |
| **Multi-source Weighted-Ensemble Precipitation** | Annual | Princeton University | Beck, H.E., A.I.J.M. van Dijk, V. Levizzani, J. Schellekens, D.G. Miralles, B. Martens, A. de Roo: MSWEP: 3-hourly 0.25 global gridded precipitation (1979-2015) by merging gauge, satellite, and reanalysis data, Hydrology and Earth System Sciences, 21(1), 589-615, 2017.<br><br>Available at: https://data.princetonclimate.com/opendap. | 419634 |

| | | | | |
|---|---|---|---|---|
| **Tasseled cap brightness** | Annual | MODIS | Lobser, S.E., Cohen, W.B. MODIS tasseled cap: land cover characteristics expressed through transformed MODIS data. International Journal of Remote Sensing. 28(22): 5079-5101 (2007).<br><br>Available at: https://modis.gsfc.nasa.gov/data/dataprod/mod43.php | 420102 |
| **Tasseled cap wetness** | Annual | MODIS | Lobser, S.E., Cohen, W.B. MODIS tasseled cap: land cover characteristics expressed through transformed MODIS data. International Journal of Remote Sensing. 28(22): 5079-5101 (2007).<br><br>Available at: https://modis.gsfc.nasa.gov/data/dataprod/mod43.php. | 420102 |
| **Average land surface temperature** | Annual | MODIS | Weiss, D. J. et al. An effective approach for gapfilling continental scale remotely sensed timeseries. Isprs J. Photogramm. Remote Sens. 98, 106–118 (2014).<br><br>Available at https://modis.gsfc.nasa.gov/data/dataprod/mod11.php | 420096 |
| **Daytime land surface temperature** | Annual | MODIS | Weiss, D. J. et al. An effective approach for gapfilling continental scale remotely sensed timeseries. Isprs J. Photogramm. Remote Sens. 98, 106–118 (2014).<br><br>Available at https://modis.gsfc.nasa.gov/data/dataprod/mod11.php | 420096 |
| **Difference between daytime and nighttime land surface temperature** | Annual | MODIS | Weiss, D. J. et al. An effective approach for gapfilling continental scale remotely sensed timeseries. Isprs J. Photogramm. Remote Sens. 98, 106–118 (2014).<br><br>Available at https://modis.gsfc.nasa.gov/data/dataprod/mod11.php | 420096 |
| **Nighttime land surface temperature** | Annual | MODIS | Weiss, D. J. et al. An effective approach for gapfilling continental scale remotely sensed timeseries. Isprs J. Photogramm. Remote Sens. 98, 106–118 (2014).<br><br>Available at https://modis.gsfc.nasa.gov/data/dataprod/mod11.php | 420096 |
| **Mean years of education among 15–49 year-old females (maternal education)** | Annual | IHME | Graetz, N., Woyczynski, L., Wilson, K.F. et al. Mapping disparities in education across low- and middle-income countries. Nature 577, 235–238 (2020).<br><br>Available at: http://internal-ghdx.healthdata.org/record/ihme-data/lmic-education-geospatial-estimates-2000-2017\ | N/A |

*Supplementary Table 7:* Covariates used per region in stacked generalisation ensemble modelling after variance inflation factor algorithm for covariate selection
Regions are defined as the following: Andean South America [*ANSA*], Central Asia Eastern Europe [*CAEU*], Caribbean [*CRBN]*, Central sub-Saharan Africa [*CSSA*], Central America [*CTAM*], East Asia [*EAAS*], East sub-Saharan Africa [*ESSA*], North Africa and Middle East [*NAME*], South East Asia, [*SEAS*], South Asia [*SOAS*], Southern sub-Saharan Africa [*SSSA*], Tropical South America [*TRSA*], and Western sub-Saharan Africa [*WSSA*]. A "Yes" indicates the covariate was selected per region and a "No" indicates the covariate was not selected.

| **Covariate** | **ANSA** | **CAEU** | **CRBN** | **CSSA** | **CTAM** | **EAAS** | **ESSA** | **NAME** | **SEAS** | **SOAS** | **SSSA** | **TRSA** | **WSSA** |
|---|---|---|---|---|---|---|---|---|---|---|---|---|---|
| **Access to roads** | Yes | Yes | Yes | Yes | Yes | Yes | Yes | Yes | Yes | Yes | Yes | Yes | Yes |
| **Distance from rivers or lakes** | Yes | Yes | Yes | Yes | Yes | Yes | Yes | Yes | Yes | Yes | Yes | Yes | Yes |
| **Elevation** | No | No | Yes | No | No | Yes | Yes | Yes | Yes | Yes | Yes | Yes | Yes |
| **Population** | Yes | Yes | Yes | Yes | Yes | Yes | Yes | Yes | Yes | Yes | Yes | Yes | Yes |
| **Urban or rural** | Yes | Yes | Yes | Yes | Yes | Yes | Yes | Yes | Yes | Yes | Yes | Yes | Yes |
| **Irrigation** | Yes | Yes | Yes | Yes | Yes | Yes | Yes | Yes | Yes | Yes | Yes | Yes | Yes |
| **Cloud cover percentage** | Yes | Yes | Yes | Yes | Yes | No | Yes | Yes | Yes | No | Yes | No | Yes |
| **Mean diurnal temperature range** | Yes | Yes | Yes | Yes | Yes | Yes | Yes | Yes | Yes | Yes | Yes | Yes | No |
| **Frost day frequency** | Yes | No | Yes | Yes | Yes | No | Yes | No | Yes | Yes | No | Yes | Yes |
| **Mean potential evapotranspiration per day per month** | Yes | No | Yes | No | No | Yes | Yes | No | Yes | Yes | Yes | Yes | No |
| **Average daily minimum temperature** | No | No | No | No | No | No | No | No | No | No | No | No | Yes |
| **Average daily mean temperature** | No | No | No | No | No | No | No | No | No | No | No | No | No |
| **Average daily maximum temperature** | No | No | No | Yes | Yes | No | No | No | Yes | No | No | Yes | No |
| **Mean vapour pressure** | No | Yes | Yes | No | No | No | No | Yes | No | No | No | No | No |

| | | | | | | | | | | | | | |
|---|---|---|---|---|---|---|---|---|---|---|---|---|---|
| **Monthly wet day frequency** | Yes | Yes | Yes | Yes | No | Yes | Yes | No | Yes | Yes | Yes | No | No |
| **Enhanced vegetation index** | No | No | No | No | No | No | No | No | Yes | Yes | No | Yes | No |
| **Growing season length** | No | Yes | Yes | No | No | No | No | Yes | Yes | No | Yes | Yes | No |
| **Normalised difference vegetation index** | Yes | Yes | Yes | Yes | Yes | No | Yes | Yes | Yes | Yes | Yes | Yes | Yes |
| **Multi-source Weighted-Ensemble Precipitation** | Yes | Yes | Yes | Yes | Yes | Yes | Yes | Yes | Yes | Yes | Yes | Yes | Yes |
| **Tasseled cap brightness** | Yes | Yes | Yes | Yes | Yes | Yes | Yes | Yes | Yes | Yes | No | Yes | Yes |
| **Tasseled cap wetness** | Yes | Yes | No | No | Yes | Yes | No | No | No | Yes | Yes | No | No |
| **Daytime land surface temperature** | Yes | No | No | No | Yes | No | No | No | No | No | No | No | No |
| **Nighttime land surface temperature** | No | No | Yes | Yes | No | No | No | No | No | No | No | No | Yes |
| **Difference between daytime and nighttime land surface temperature** | No | Yes | Yes | No | No | No | Yes | Yes | Yes | No | Yes | No | Yes |
| **Maternal education** | Yes | Yes | Yes | No | Yes | Yes | Yes | Yes | Yes | Yes | Yes | Yes | Yes |

*Supplementary Table 8:* Fitted model parameters and final penalised control prior specification
Means and upper and lower quantiles of fitted model parameters for the posterior distribution are given by region. Final penalised control prior $\boldsymbol{\rho_0}$ selection also reported per region. Regions are defined as the following: Andean South America [*ANSA*], Central Asia Eastern Europe [*CAEU*], Caribbean [*CRBN]*, Central sub-Saharan Africa [*CSSA*], Central America [*CTAM*], East Asia [*EAAS*], East sub-Saharan Africa [*ESSA*], North Africa and Middle East [*NAME*], South East Asia, [*SEAS*], South Asia [*SOAS*], Southern sub-Saharan Africa [*SSSA*], Tropical South America [*TRSA*], and Western sub-Saharan Africa [*WSSA*].

| Region | Quantile | Intercept | GAM | Lasso | GBM | Nominal range | $\rho_0$ | Nominal variance | AR1 ρ | Precision | Country random effect precision |
|---|---|---|---|---|---|---|---|---|---|---|---|
| **ANSA** | 0.025 | -0.096 | -0.069 | -0.451 | 0.741 | 0.051 | -- | 0.188 | 0.858 | 483.038 | 116.753 |
| | 0.500 | 0.16 | 0.122 | -0.165 | 1.043 | 0.078 | 0.039 | 0.321 | 0.919 | 2350.766 | 1271.491 |
| | 0.975 | 0.416 | 0.313 | 0.120 | 1.345 | 0.116 | -- | 0.526 | 0.952 | 8875.4 | 6886.637 |
| **CAEU** | 0.025 | -0.022 | 0.227 | -0.382 | 0.362 | 0.024 | -- | 0.166 | 0.660 | 267.156 | 71.467 |
| | 0.500 | 0.121 | 0.451 | -0.118 | 0.666 | 0.036 | 0.033 | 0.249 | 0.799 | 1706.392 | 1073.213 |
| | 0.975 | 0.264 | 0.677 | 0.145 | 0.970 | 0.052 | -- | 0.358 | 0.877 | 8356.505 | 6743.526 |
| **CRBN** | 0.025 | -0.037 | -0.051 | -0.339 | 0.751 | 0.008 | -- | 0.103 | 0.728 | 4.249 | 183.241 |
| | 0.500 | 0.097 | 0.150 | -0.135 | 0.985 | 0.013 | 0.028 | 0.168 | 0.873 | 6.448 | 1409.929 |
| | 0.975 | 0.230 | 0.351 | 0.068 | 1.220 | 0.022 | -- | 0.255 | 0.932 | 9.492 | 8238.198 |
| **CSSA** | 0.025 | -0.381 | -0.091 | -0.105 | 0.744 | 0.045 | -- | 0.451 | 0.852 | 732.748 | 10.315 |
| | 0.500 | -0.165 | 0.057 | 0.067 | 0.875 | 0.052 | 0.034 | 0.535 | 0.879 | 2750.245 | 52.537 |
| | 0.975 | 0.051 | 0.206 | 0.239 | 1.006 | 0.060 | -- | 0.644 | 0.903 | 10671.613 | 252.011 |
| **CTAM** | 0.025 | -0.302 | -0.175 | -0.388 | 0.932 | 0.068 | -- | 0.390 | 0.763 | 286.604 | 116.760 |
| | 0.500 | -0.024 | -0.008 | -0.145 | 1.153 | 0.102 | 0.054 | 0.597 | 0.855 | 1878.183 | 1279.972 |
| | 0.975 | 0.254 | 0.160 | 0.097 | 1.374 | 0.159 | -- | 0.941 | 0.918 | 8353.858 | 6701.157 |
| **EAAS** | 0.025 | -0.316 | -0.228 | -0.082 | 0.140 | 0.045 | -- | 0.246 | 0.567 | 180.566 | 149.156 |
| | 0.500 | 0.079 | 0.185 | 0.324 | 0.491 | 0.087 | 0.045 | 0.547 | 0.798 | 1631.491 | 1439.261 |
| | 0.975 | 0.474 | 0.595 | 0.731 | 0.843 | 0.153 | -- | 1.066 | 0.905 | 8095.035 | 8197.362 |
| **ESSA** | 0.025 | -0.058 | 0.109 | -0.036 | 0.511 | 0.031 | -- | 0.956 | 0.872 | 11.950 | 15.820 |
| | 0.500 | 0.126 | 0.264 | 0.111 | 0.625 | 0.034 | 0.048 | 1.088 | 0.890 | 14.666 | 26.269 |
| | 0.975 | 0.309 | 0.420 | 0.258 | 0.738 | 0.038 | -- | 1.202 | 0.903 | 18.344 | 55.881 |
| **NAME** | 0.025 | 0.016 | -0.064 | -0.070 | 0.759 | 0.048 | -- | 0.649 | 0.878 | 774.166 | 135.331 |
| | 0.500 | 0.224 | 0.020 | 0.078 | 0.889 | 0.057 | 0.061 | 0.816 | 0.905 | 2800.478 | 1247.523 |
| | 0.975 | 0.433 | 0.132 | 0.219 | 1.019 | 0.068 | -- | 1.028 | 0.925 | 9871.623 | 6942.145 |

| | | | | | | | | | | | |
|---|---|---|---|---|---|---|---|---|---|---|---|
| **SEAS** | 0.025 | 0.192 | 0.314 | 0.125 | 0.075 | 0.192 | -- | 0.629 | 0.931 | 902.225 | 5.223 |
| | 0.500 | 0.322 | 0.461 | 0.216 | 0.094 | 0.322 | 0.051 | 0.892 | 0.95 | 3170.129 | 19.516 |
| | 0.975 | 0.452 | 0.607 | 0.309 | 0.110 | 0.452 | -- | 1.145 | 0.96 | 11388.874 | 137.374 |
| **SOAS** | 0.025 | -0.017 | 0.075 | -0.003 | 0.701 | 0.023 | -- | 0.718 | 0.924 | 501.285 | 2.430 |
| | 0.500 | 0.272 | 0.151 | 0.077 | 0.772 | 0.025 | 0.036 | 0.814 | 0.934 | 2315.602 | 11.398 |
| | 0.975 | 0.561 | 0.227 | 0.157 | 0.843 | 0.027 | -- | 0.887 | 0.940 | 10218.148 | 42.347 |
| **SSSA** | 0.025 | 0.036 | -0.142 | -0.442 | 0.969 | 0.025 | -- | 0.244 | 0.563 | 336.787 | 186.477 |
| | 0.500 | 0.157 | 0.038 | -0.217 | 1.179 | 0.034 | 0.025 | 0.319 | 0.703 | 1918.747 | 1563.802 |
| | 0.975 | 0.277 | 0.218 | 0.007 | 1.390 | 0.048 | -- | 0.419 | 0.798 | 8854.038 | 7359.890 |
| **TRSA** | 0.025 | -0.441 | -0.072 | -0.044 | 0.473 | 0.078 | -- | 0.217 | 0.531 | 206.004 | 144.793 |
| | 0.500 | 0.067 | -0.033 | 0.257 | 0.773 | 0.147 | 0.044 | 0.605 | 0.833 | 1628.357 | 1394.899 |
| | 0.975 | 0.576 | 0.013 | 0.556 | 1.074 | 0.299 | -- | 1.771 | 0.952 | 7627.442 | 7199.690 |
| **WSSA** | 0.025 | -0.104 | 0.061 | 0.126 | 0.547 | 0.028 | -- | 0.724 | 0.858 | 24.589 | 21.278 |
| | 0.500 | 0.029 | 0.150 | 0.235 | 0.614 | 0.030 | 0.049 | 0.796 | 0.870 | 35.217 | 60.381 |
| | 0.975 | 0.162 | 0.246 | 0.339 | 0.682 | 0.031 | -- | 0.852 | 0.878 | 65.410 | 208.613 |

*Supplementary Table 9a–b:* First administrative level in-sample fit metrics
In-sample fit statistics are provided for first administrative levels, by both year and modelling region, along with the number of surveys per year. Regions are defined as the following: Andean South America [*ANSA*], Central Asia Eastern Europe [*CAEU*], Caribbean [*CRBN]*, Central sub-Saharan Africa [*CSSA*], Central America [*CTAM*], East Asia [*EAAS*], East sub-Saharan Africa [*ESSA*], North Africa and Middle East [*NAME*], South East Asia, [*SEAS*], South Asia [*SOAS*], Southern sub-Saharan Africa [*SSSA*], Tropical South America [*TRSA*], and Western sub-Saharan Africa [*WSSA*].

a) Predictive in-sample metrics by year

| **Year** | **# surveys** | **Mean Error** | **Absolute Mean Error** | **RMSE** | **Median SS** | **Correlation** | **95% Cov** |
|---|---|---|---|---|---|---|---|
| **2000** | 33 | 0.00053692 | 0.02635373 | 0.04231509 | 46.0363422 | 0.97994849 | 0.97877094 |
| **2001** | 37 | 0.0009708 | 0.02605133 | 0.04024005 | 44.6298732 | 0.97737563 | 0.97554964 |
| **2002** | 56 | 2.34E-05 | 0.02424297 | 0.03745372 | 42 | 0.98443402 | 0.9636974 |
| **2003** | 72 | 0.00243208 | 0.02095623 | 0.03623518 | 43.0737074 | 0.97992682 | 0.95475457 |
| **2004** | 75 | 0.00021764 | 0.02210362 | 0.03725476 | 51.8042602 | 0.98083166 | 0.96617166 |
| **2005** | 67 | 0.00129004 | 0.02162749 | 0.03718889 | 51.9133479 | 0.9817879 | 0.96095419 |
| **2006** | 44 | -0.0024252 | 0.023592 | 0.0364414 | 49 | 0.98383887 | 0.95499233 |
| **2007** | 55 | -0.0011763 | 0.02287269 | 0.0372812 | 39 | 0.98268483 | 0.96655247 |
| **2008** | 61 | 0.00293164 | 0.02198287 | 0.03474891 | 49.3144316 | 0.98064814 | 0.95838031 |
| **2009** | 80 | 0.00092838 | 0.01805624 | 0.02979466 | 54.0691397 | 0.98451572 | 0.94511165 |
| **2010** | 65 | -0.0005564 | 0.02004318 | 0.03082232 | 63 | 0.98573271 | 0.94623312 |
| **2011** | 56 | 0.00046661 | 0.01823046 | 0.02828082 | 61.6804272 | 0.98690118 | 0.96439813 |
| **2012** | 57 | 0.00154442 | 0.01512939 | 0.02533908 | 72 | 0.98830327 | 0.97722976 |
| **2013** | 51 | 0.00125432 | 0.0142874 | 0.02439052 | 89 | 0.98751972 | 0.97659502 |
| **2014** | 39 | -0.0006373 | 0.01540902 | 0.02823571 | 60 | 0.98658157 | 0.98080389 |
| **2015** | 30 | 0.01347675 | 0.03553002 | 0.04983321 | 96.5 | 0.97359943 | 0.82523967 |
| **2016** | 25 | 0.00071699 | 0.02007698 | 0.03075514 | 137 | 0.98372424 | 0.96828173 |
| **2017** | 12 | -0.0016224 | 0.01913547 | 0.02805477 | 117.937189 | 0.9906914 | 0.98273656 |
| **2018** | 2 | -0.0009848 | 0.02614406 | 0.03233535 | 93.3276923 | 0.98519003 | 0.98927971 |

b) Predictive in-sample metrics by region

| Region | Mean Error | Absolute Mean Error | RMSE | Median SS | Correlation | 95% Cov |
|---|---|---|---|---|---|---|
| **ANSA** | 5.11E-05 | 0.01637548 | 0.02509985 | 134.468578 | 0.9156748 | 0.91890569 |
| **CAEU** | 0.00043577 | 0.02298082 | 0.03273594 | 16.3927678 | 0.90704614 | 0.96232358 |
| **CRBN** | 0.00113856 | 0.03177861 | 0.04568725 | 46 | 0.92520688 | 0.98642848 |
| **CSSA** | 0.00020721 | 0.02810706 | 0.04010708 | 93 | 0.97394071 | 0.98093556 |
| **CTAM** | 0.00073975 | 0.01918668 | 0.0281529 | 59.5419267 | 0.9069162 | 0.96960494 |
| **EAAS** | 0.00177776 | 0.02889506 | 0.04342324 | 27.036481 | 0.88900484 | 0.9725173 |
| **ESSA** | 0.00063204 | 0.02084235 | 0.03772253 | 30 | 0.97831948 | 0.9817295 |
| **NAME** | 0.00171722 | 0.01971767 | 0.03230932 | 77 | 0.98587959 | 0.96784038 |
| **SEAS** | 0.00526308 | 0.03746742 | 0.05447697 | 16.1065991 | 0.94308048 | 0.88517549 |
| **SOAS** | 0.00052722 | 0.00875791 | 0.0148395 | 209.556704 | 0.99401275 | 0.96138823 |
| **SSSA** | 0.00074742 | 0.02030037 | 0.03027176 | 79 | 0.95790943 | 0.98497672 |
| **TRSA** | 0.00222705 | 0.0309591 | 0.04262648 | 35 | 0.88806962 | 0.9705352 |
| **WSSA** | 0.00065904 | 0.02168152 | 0.0312355 | 129.274825 | 0.99044181 | 0.9659038 |

*Supplementary Table 10a–b:* Second administrative level in-sample fit metrics
In-sample fit statistics are provided for second administrative levels, by both year and modelling region, along with the number of surveys per year. Regions are defined as the following: Andean South America [*ANSA*], Central Asia Eastern Europe [*CAEU*], Caribbean [*CRBN]*, Central sub-Saharan Africa [*CSSA*], Central America [*CTAM*], East Asia [*EAAS*], East sub-Saharan Africa [*ESSA*], North Africa and Middle East [*NAME*], South East Asia, [*SEAS*], South Asia [*SOAS*], Southern sub-Saharan Africa [*SSSA*], Tropical South America [*TRSA*], and Western sub-Saharan Africa [*WSSA*].

a) Predictive in-sample metrics by year

| Year | # surveys | Mean Error | Absolute Mean Error | RMSE | Median SS | Correlation | 95% Cov |
|---|---|---|---|---|---|---|---|
| **2000** | 33 | 0.00055443 | 0.05654278 | 0.09811363 | 4 | 0.90842575 | 0.97912349 |
| **2001** | 37 | 0.0009936 | 0.05522176 | 0.09651392 | 4 | 0.89288495 | 0.97571901 |
| **2002** | 56 | 5.82E-05 | 0.04932417 | 0.08542299 | 4.82131901 | 0.92791321 | 0.96448719 |
| **2003** | 72 | 0.00245723 | 0.04297837 | 0.07219868 | 5.08379584 | 0.93061094 | 0.95386247 |
| **2004** | 75 | 0.00011671 | 0.04569372 | 0.07704234 | 5.38316321 | 0.9277682 | 0.96634161 |
| **2005** | 67 | 0.00122365 | 0.04515145 | 0.07299875 | 7 | 0.93828568 | 0.95923504 |
| **2006** | 44 | -0.0025486 | 0.04849309 | 0.07650222 | 7.16909864 | 0.93591081 | 0.95487512 |
| **2007** | 55 | -0.0009849 | 0.04796788 | 0.08243783 | 5 | 0.92731906 | 0.96855446 |
| **2008** | 61 | 0.00300661 | 0.04014995 | 0.06701189 | 5 | 0.93616091 | 0.9596579 |
| **2009** | 80 | 0.00108027 | 0.03742898 | 0.06477792 | 4.81470714 | 0.93960644 | 0.94748026 |
| **2010** | 65 | -0.0005959 | 0.04472063 | 0.07132594 | 6.82345885 | 0.93786648 | 0.94465938 |
| **2011** | 56 | 0.00048929 | 0.04877053 | 0.07925216 | 6.47812137 | 0.91824284 | 0.96421503 |
| **2012** | 57 | 0.00164323 | 0.04506436 | 0.07179587 | 8 | 0.93015831 | 0.97752475 |
| **2013** | 51 | 0.0013939 | 0.04064388 | 0.06380981 | 7.03390651 | 0.93644966 | 0.97626159 |
| **2014** | 39 | -0.0007658 | 0.04580421 | 0.07261361 | 6 | 0.93269463 | 0.98153882 |
| **2015** | 30 | 0.01383076 | 0.06062786 | 0.0877248 | 12 | 0.92388174 | 0.82729539 |
| **2016** | 25 | 0.00059757 | 0.04860972 | 0.07904682 | 11 | 0.91943344 | 0.97175523 |
| **2017** | 12 | -0.0012481 | 0.06393478 | 0.09924336 | 9 | 0.91149812 | 0.98034004 |
| **2018** | 2 | -0.0016765 | 0.03281482 | 0.04229367 | 11.8941708 | 0.97411665 | 0.98962449 |

b) Predictive in-sample metrics by region

| | Mean Error | Absolute Mean Error | RMSE | Median SS | Correlation | 95% Cov |
|---|---|---|---|---|---|---|
| **ANSA** | 8.39E-05 | 0.03686722 | 0.06324687 | 9.25872298 | 0.68455009 | 0.91765183 |
| **CAEU** | 0.00052046 | 0.03597987 | 0.05529659 | 1.6357065 | 0.79865872 | 0.96322514 |
| **CRBN** | 0.00100688 | 0.05451513 | 0.08113386 | 9 | 0.82208926 | 0.98842882 |
| **CSSA** | 9.54E-05 | 0.0555715 | 0.08770973 | 9 | 0.90516314 | 0.98036271 |
| **CTAM** | 0.0006236 | 0.04386879 | 0.08254381 | 2 | 0.60240256 | 0.97027574 |
| **EAAS** | 0.00204573 | 0.06911484 | 0.11563918 | 1.35976537 | 0.62498241 | 0.97201757 |
| **ESSA** | 0.0006909 | 0.04544783 | 0.07583879 | 10 | 0.9251329 | 0.98189334 |
| **NAME** | 0.00176904 | 0.03817814 | 0.06700717 | 5 | 0.94340503 | 0.96706453 |
| **SEAS** | 0.00544513 | 0.06349269 | 0.10428847 | 3 | 0.83191054 | 0.88778324 |
| **SOAS** | 0.00056308 | 0.03499376 | 0.05106628 | 18.188021 | 0.9539919 | 0.9612275 |
| **SSSA** | 0.00091458 | 0.04383531 | 0.06695007 | 15.2583897 | 0.84967729 | 0.98568338 |
| **TRSA** | 0.00196177 | 0.0432823 | 0.07032544 | 3.65083624 | 0.75854069 | 0.96925537 |
| **WSSA** | 0.00067849 | 0.05308181 | 0.08344501 | 9 | 0.94175485 | 0.96661521 |

*Supplementary Table 11a–b:* First administrative level out-of-sample fit metrics

Out-of-sample fit statistics are provided first administrative levels, by both year and modelling region, along with the number of surveys per year. Regions are defined as the following: Andean South America [*ANSA*], Central Asia Eastern Europe [*CAEU*], Caribbean [*CRBN]*, Central sub-Saharan Africa [*CSSA*], Central America [*CTAM*], East Asia [*EAAS*], East sub-Saharan Africa [*ESSA*], North Africa and Middle East [*NAME*], South East Asia, [*SEAS*], South Asia [*SOAS*], Southern sub-Saharan Africa [*SSSA*], Tropical South America [*TRSA*], and Western sub-Saharan Africa [*WSSA*].

a) Predictive out-of-sample metrics by year

| Year | # surveys | Mean Error | Absolute Mean Error | RMSE | Median SS | Correlation | 95% Cov |
|---|---|---|---|---|---|---|---|
| **2000** | 33 | 0.00109608 | 0.05892839 | 0.08701755 | 46.0363422 | 0.90870324 | 0.93675611 |
| **2001** | 37 | 0.00604213 | 0.0532355 | 0.08293942 | 44.6298732 | 0.90235839 | 0.90739097 |
| **2002** | 56 | 0.00423353 | 0.07072861 | 0.10108503 | 42 | 0.87802169 | 0.8929278 |
| **2003** | 72 | 0.01007656 | 0.06968165 | 0.10026723 | 43.0737074 | 0.83821621 | 0.9040794 |
| **2004** | 75 | 0.00201912 | 0.06638141 | 0.09497559 | 51.8042602 | 0.86485974 | 0.91461614 |
| **2005** | 67 | 0.00737509 | 0.0614735 | 0.08816797 | 51.9133479 | 0.89244413 | 0.92099329 |
| **2006** | 44 | -0.0019245 | 0.05374569 | 0.07677644 | 49 | 0.92331759 | 0.92384506 |
| **2007** | 55 | 0.00370613 | 0.0581578 | 0.08810045 | 39 | 0.89710094 | 0.91424082 |
| **2008** | 61 | 0.01334289 | 0.05877675 | 0.08935583 | 49.3144316 | 0.8709797 | 0.90457104 |
| **2009** | 80 | 0.01533936 | 0.05951215 | 0.08277292 | 54.0691397 | 0.88274276 | 0.90919968 |
| **2010** | 65 | 0.00443667 | 0.06209517 | 0.09188177 | 63 | 0.86255137 | 0.90610095 |
| **2011** | 56 | 0.00972485 | 0.05753966 | 0.08487157 | 61.6804272 | 0.8783782 | 0.93934 |
| **2012** | 57 | 0.00647198 | 0.06522642 | 0.09535599 | 72 | 0.82011745 | 0.93209742 |
| **2013** | 50 | 0.00546607 | 0.06826785 | 0.09609485 | 89 | 0.78241634 | 0.9283279 |
| **2014** | 39 | 0.00033541 | 0.07196178 | 0.10376123 | 60 | 0.80460077 | 0.94465868 |
| **2015** | 30 | 0.01497122 | 0.07367801 | 0.09938328 | 96.5 | 0.87217776 | 0.8049238 |
| **2016** | 25 | 0.00293919 | 0.0660663 | 0.0965227 | 137 | 0.82945943 | 0.91802663 |
| **2017** | 12 | 0.00459285 | 0.07279136 | 0.10221884 | 117.937189 | 0.86442152 | 0.92541822 |
| **2018** | 2 | 0.00345032 | 0.10441635 | 0.12606068 | 93.3276923 | 0.76193256 | 0.78226911 |

b) Predictive out-of-sample metrics by region

| Region | Mean Error | Absolute Mean Error | RMSE | Median SS | Correlation | 95% Cov |
|---|---|---|---|---|---|---|
| **ANSA** | -0.0035869 | 0.03406898 | 0.04641319 | 134.468578 | 0.6409674 | 0.91809894 |
| **CAEU** | 0.00031804 | 0.04532475 | 0.0664237 | 16.3927678 | 0.5735325 | 0.8485832 |
| **CRBN** | 0.00502411 | 0.05673316 | 0.07855574 | 46 | 0.75792476 | 0.96676594 |
| **CSSA** | 0.0163004 | 0.10252405 | 0.13353253 | 93 | 0.64316062 | 0.8873901 |
| **CTAM** | 0.0028507 | 0.03133523 | 0.04382939 | 59.5419267 | 0.75196409 | 0.94480988 |
| **EAAS** | 0.01243832 | 0.07463007 | 0.10013111 | 27.036481 | 0.08308726 | 0.92658645 |
| **ESSA** | 0.0082887 | 0.06129358 | 0.09423161 | 30 | 0.85744421 | 0.9506731 |
| **NAME** | 0.00333958 | 0.06466844 | 0.11056161 | 77 | 0.81492986 | 0.88321762 |
| **SEAS** | 0.00553729 | 0.04345195 | 0.06715842 | 16.1065991 | 0.9094903 | 0.87136797 |
| **SOAS** | 0.00725594 | 0.07158899 | 0.09599978 | 209.556704 | 0.71154827 | 0.92697774 |
| **SSSA** | -0.0040252 | 0.05271564 | 0.07651169 | 79 | 0.67767687 | 0.95339528 |
| **TRSA** | -0.0042544 | 0.0606068 | 0.08404111 | 35 | 0.45380718 | 0.87566424 |
| **WSSA** | 0.00885015 | 0.07753701 | 0.10246178 | 129.274825 | 0.88974842 | 0.89856221 |

*Supplementary Table 12a–b:* Second administrative level out-of-sample fit metrics
Out-of-sample fit statistics are provided for second administrative levels, by both year and modelling region, along with the number of surveys per year. Regions are defined as the following: Andean South America [*ANSA*], Central Asia Eastern Europe [*CAEU*], Caribbean [*CRBN]*, Central sub-Saharan Africa [*CSSA*], Central America [*CTAM*], East Asia [*EAAS*], East sub-Saharan Africa [*ESSA*], North Africa and Middle East [*NAME*], South East Asia, [*SEAS*], South Asia [*SOAS*], Southern sub-Saharan Africa [*SSSA*], Tropical South America [*TRSA*], and Western sub-Saharan Africa [*WSSA*].

a) Predictive out-of-sample metrics by year

| Year | # surveys | Mean Error | Absolute Mean Error | RMSE | Median SS | Correlation | 95% Cov |
|---|---|---|---|---|---|---|---|
| **2000** | 33 | 0.00270406 | 0.07782518 | 0.12262384 | 4 | 0.8506162 | 0.95151268 |
| **2001** | 37 | 0.00220842 | 0.07136626 | 0.11528163 | 4 | 0.84271056 | 0.94030888 |
| **2002** | 56 | 0.00257876 | 0.07065943 | 0.1104081 | 4.82131901 | 0.87541732 | 0.92823263 |
| **2003** | 72 | 0.0046717 | 0.06723186 | 0.10182437 | 5.08379584 | 0.85709933 | 0.91830692 |
| **2004** | 75 | -1.718E-05 | 0.06922424 | 0.10471612 | 5.38316321 | 0.86102734 | 0.94019813 |
| **2005** | 67 | 0.00102664 | 0.06938524 | 0.10220661 | 7 | 0.87428723 | 0.9370929 |
| **2006** | 44 | -0.0041778 | 0.06828019 | 0.10266948 | 7.16909864 | 0.87996406 | 0.92207539 |
| **2007** | 55 | -0.0010841 | 0.07240422 | 0.11454926 | 5 | 0.85230679 | 0.93570283 |
| **2008** | 61 | 0.00428895 | 0.06157605 | 0.09733453 | 5 | 0.86058997 | 0.93435323 |
| **2009** | 80 | 0.00169011 | 0.06273314 | 0.09849461 | 4.81470787 | 0.85398882 | 0.91301816 |
| **2010** | 65 | -0.0026736 | 0.07146478 | 0.10593297 | 6.82345825 | 0.85518859 | 0.90667115 |
| **2011** | 56 | 0.00144875 | 0.07293265 | 0.11208121 | 6.47812221 | 0.8283444 | 0.93889497 |
| **2012** | 57 | 0.0015987 | 0.07452569 | 0.10897109 | 8 | 0.8288919 | 0.94274301 |
| **2013** | 51 | 0.00171151 | 0.0708803 | 0.10269853 | 7.03390651 | 0.82265477 | 0.93944086 |
| **2014** | 39 | -0.001167 | 0.07850075 | 0.11242676 | 6 | 0.82800796 | 0.9532038 |
| **2015** | 30 | 0.01464069 | 0.07984529 | 0.11241412 | 12 | 0.86561962 | 0.80632746 |
| **2016** | 25 | 0.00069926 | 0.07540918 | 0.11391859 | 11 | 0.82240423 | 0.92469919 |
| **2017** | 12 | -0.003244 | 0.10508367 | 0.14553064 | 9 | 0.79498885 | 0.93423726 |
| **2018** | 2 | 0.00774015 | 0.06367272 | 0.09124818 | 11.8941708 | 0.87933984 | 0.90504853 |

b) Predictive out-of-sample metrics by region

| Region | Mean Error | Absolute Mean Error | RMSE | Median SS | Correlation | 95% Cov |
|---|---|---|---|---|---|---|
| **ANSA** | -0.0018076 | 0.04556895 | 0.0724233 | 9.25872298 | 0.53934742 | 0.91629933 |
| **CAEU** | 0.00106486 | 0.04704413 | 0.07091812 | 1.63570656 | 0.63018562 | 0.90066858 |
| **CRBN** | 0.00224646 | 0.07014859 | 0.10028029 | 9 | 0.70439486 | 0.97665381 |
| **CSSA** | 0.00171496 | 0.09110177 | 0.1326402 | 9 | 0.75815441 | 0.94919553 |
| **CTAM** | 0.0012037 | 0.04914668 | 0.08774568 | 2 | 0.52625109 | 0.95486638 |
| **EAAS** | 0.024974 | 0.10548164 | 0.14280062 | 1.35976537 | 0.31256475 | 0.90264126 |
| **ESSA** | 0.00215741 | 0.07170412 | 0.10922689 | 10 | 0.83783816 | 0.96159855 |
| **NAME** | 0.0023493 | 0.05327719 | 0.08958711 | 5 | 0.89547121 | 0.93217578 |
| **SEAS** | 0.00619067 | 0.06712985 | 0.10825644 | 3 | 0.81676836 | 0.8727628 |
| **SOAS** | -0.00021 | 0.06964078 | 0.09894883 | 18.188021 | 0.80999656 | 0.92504512 |
| **SSSA** | -0.0034354 | 0.06187324 | 0.09156887 | 15.2583897 | 0.68677512 | 0.97661508 |
| **TRSA** | 0.00498065 | 0.06784998 | 0.09625923 | 3.65083624 | 0.44838905 | 0.89373318 |
| **WSSA** | 0.00085017 | 0.0904069 | 0.12762859 | 9 | 0.85638995 | 0.91159305 |

*Supplementary Table 13:* Gini coefficients in 2000 and 2019
Gini coefficients were calculated from corresponding Lorenz curves in 2000 and 2019 and used in calculations of absolute inequality.

| Country | Mean MCV1 Coverage | Gini Coefficient | Absolute Inequality | Year |
|---|---|---|---|---|
| **Afghanistan** | 34.9% | 0.132 | 0.0919 | 2000 |
| | 57.5% | 0.121 | 0.1396 | 2019 |
| **Angola** | 52.5% | 0.215 | 0.2257 | 2019 |
| | 64.0% | 0.100 | 0.1281 | 2000 |
| **Armenia** | 87.8% | 0.010 | 0.0181 | 2000 |
| | 93.7% | 0.005 | 0.0098 | 2019 |
| **Azerbaijan** | 65.5% | 0.070 | 0.0919 | 2000 |
| | 88.6% | 0.015 | 0.0262 | 2019 |
| **Burundi** | 83.2% | 0.034 | 0.0571 | 2000 |
| | 91.2% | 0.015 | 0.0269 | 2019 |
| **Benin** | 71.7% | 0.055 | 0.0792 | 2000 |
| | 75.7% | 0.053 | 0.0805 | 2019 |
| **Burkina Faso** | 64.4% | 0.112 | 0.1442 | 2000 |
| | 95.5% | 0.007 | 0.0142 | 2019 |
| **Bangladesh** | 77.7% | 0.056 | 0.0863 | 2000 |
| | 93.2% | 0.013 | 0.0249 | 2019 |
| **Belize** | 86.3% | 0.026 | 0.0456 | 2000 |
| | 90.7% | 0.010 | 0.0190 | 2019 |
| **Bolivia** | 86.0% | 0.041 | 0.0697 | 2000 |
| | 89.0% | 0.020 | 0.0360 | 2019 |
| **Botswana** | 86.2% | 0.020 | 0.0352 | 2000 |
| | 89.0% | 0.011 | 0.0196 | 2019 |
| **Central African Republic** | 39.6% | 0.092 | 0.0729 | 2019 |
| | 45.1% | 0.088 | 0.0797 | 2000 |
| **Cote d'Ivoire** | 68.0% | 0.079 | 0.1072 | 2000 |
| | 74.3% | 0.037 | 0.0545 | 2019 |
| **Cameroon** | 67.8% | 0.109 | 0.1483 | 2019 |
| | 72.0% | 0.142 | 0.2040 | 2000 |
| **Democratic Republic of the Congo** | 48.2% | 0.165 | 0.1592 | 2000 |
| | 66.5% | 0.105 | 0.1401 | 2019 |
| **Republic of Congo** | 33.8% | 0.134 | 0.0902 | 2000 |
| | 71.5% | 0.079 | 0.1133 | 2019 |
| **Colombia** | 92.8% | 0.017 | 0.0309 | 2000 |

| | | | | |
|---|---|---|---|---|
| | 91.6% | 0.005 | 0.0091 | 2019 |
| **Comoros** | 69.8% | 0.017 | 0.0238 | 2000 |
| | 90.5% | 0.008 | 0.0139 | 2019 |
| **Cape Verde** | 81.7% | 0.016 | 0.0265 | 2000 |
| | 99.9% | 0.000 | 0.0001 | 2019 |
| **Costa Rica** | 89.5% | 0.006 | 0.0111 | 2000 |
| | 99.9% | 0.000 | 0.0002 | 2019 |
| **Cuba** | 86.2% | 0.015 | 0.0266 | 2000 |
| | 90.6% | 0.008 | 0.0147 | 2019 |
| **Djibouti** | 59.9% | 0.049 | 0.0590 | 2000 |
| | 84.6% | 0.020 | 0.0345 | 2019 |
| **Dominican Republic** | 85.7% | 0.011 | 0.0194 | 2019 |
| | 90.4% | 0.015 | 0.0265 | 2000 |
| **Algeria** | 82.7% | 0.017 | 0.0276 | 2019 |
| | 93.1% | 0.012 | 0.0227 | 2000 |
| **Ecuador** | 75.2% | 0.025 | 0.0376 | 2019 |
| | 82.5% | 0.044 | 0.0728 | 2000 |
| **Egypt** | 97.3% | 0.005 | 0.0097 | 2000 |
| | 99.3% | 0.001 | 0.0017 | 2019 |
| **Eritrea** | 86.5% | 0.038 | 0.0659 | 2000 |
| | 99.9% | 0.001 | 0.0013 | 2019 |
| **Ethiopia** | 24.2% | 0.302 | 0.1457 | 2000 |
| | 56.8% | 0.132 | 0.1495 | 2019 |
| **Gabon** | 51.6% | 0.043 | 0.0448 | 2000 |
| | 67.9% | 0.027 | 0.0371 | 2019 |
| **Ghana** | 84.0% | 0.053 | 0.0891 | 2000 |
| | 91.1% | 0.013 | 0.0240 | 2019 |
| **Guinea** | 48.2% | 0.144 | 0.1390 | 2019 |
| | 51.8% | 0.162 | 0.1679 | 2000 |
| **Gambia** | 85.5% | 0.017 | 0.0297 | 2019 |
| | 92.1% | 0.011 | 0.0203 | 2000 |
| **Guinea-Bissau** | 69.8% | 0.055 | 0.0770 | 2000 |
| | 78.1% | 0.034 | 0.0529 | 2019 |
| **Equatorial Guinea** | 43.2% | 0.066 | 0.0567 | 2000 |
| | 43.3% | 0.079 | 0.0685 | 2019 |
| **Guatemala** | 78.1% | 0.031 | 0.0481 | 2000 |
| | 84.8% | 0.007 | 0.0121 | 2019 |
| **Guyana** | 91.0% | 0.005 | 0.0093 | 2000 |
| | 94.7% | 0.002 | 0.0045 | 2019 |

| | | | | |
|---|---|---|---|---|
| **Honduras** | 87.4% | 0.015 | 0.0262 | 2000 |
| | 90.2% | 0.007 | 0.0126 | 2019 |
| **Haiti** | 70.1% | 0.077 | 0.1081 | 2000 |
| | 71.6% | 0.040 | 0.0579 | 2019 |
| **Indonesia** | 71.8% | 0.076 | 0.1092 | 2000 |
| | 80.1% | 0.050 | 0.0806 | 2019 |
| **India** | 51.5% | 0.217 | 0.2237 | 2000 |
| | 92.0% | 0.036 | 0.0669 | 2019 |
| **Iran** | 95.6% | 0.016 | 0.0312 | 2000 |
| | 99.5% | 0.004 | 0.0079 | 2019 |
| **Iraq** | 78.2% | 0.053 | 0.0826 | 2019 |
| | 81.7% | 0.033 | 0.0544 | 2000 |
| **Jamaica** | 92.3% | 0.014 | 0.0260 | 2000 |
| | 100.0% | 0.000 | 0.0001 | 2019 |
| **Jordan** | 77.7% | 0.012 | 0.0184 | 2019 |
| | 96.4% | 0.004 | 0.0081 | 2000 |
| **Kenya** | 78.4% | 0.081 | 0.1265 | 2000 |
| | 82.0% | 0.036 | 0.0595 | 2019 |
| **Kyrgyzstan** | 88.7% | 0.028 | 0.0500 | 2019 |
| | 93.7% | 0.015 | 0.0289 | 2000 |
| **Cambodia** | 63.7% | 0.093 | 0.1189 | 2000 |
| | 91.1% | 0.014 | 0.0258 | 2019 |
| **Laos** | 46.3% | 0.186 | 0.1721 | 2000 |
| | 72.9% | 0.057 | 0.0832 | 2019 |
| **Liberia** | 71.0% | 0.092 | 0.1303 | 2000 |
| | 93.1% | 0.015 | 0.0270 | 2019 |
| **Sri Lanka** | 94.5% | 0.009 | 0.0170 | 2000 |
| | 90.9% | 0.012 | 0.0223 | 2019 |
| **Lesotho** | 77.9% | 0.014 | 0.0217 | 2000 |
| | 91.0% | 0.003 | 0.0057 | 2019 |
| **Morocco** | 92.1% | 0.021 | 0.0396 | 2000 |
| | 92.0% | 0.006 | 0.0116 | 2019 |
| **Moldova** | 89.3% | 0.004 | 0.0069 | 2019 |
| | 96.0% | 0.003 | 0.0066 | 2000 |
| **Madagascar** | 58.3% | 0.147 | 0.1710 | 2000 |
| | 59.6% | 0.149 | 0.1779 | 2019 |
| **Mexico** | 67.5% | 0.035 | 0.0466 | 2019 |
| | 76.7% | 0.081 | 0.1235 | 2000 |
| **Mali** | 58.8% | 0.161 | 0.1897 | 2000 |

| | | | | |
|---|---|---|---|---|
| | 69.4% | 0.080 | 0.1104 | 2019 |
| **Myanmar** | 80.4% | 0.033 | 0.0529 | 2019 |
| | 86.8% | 0.048 | 0.0837 | 2000 |
| **Mongolia** | 83.9% | 0.027 | 0.0459 | 2019 |
| | 89.9% | 0.018 | 0.0326 | 2000 |
| **Mozambique** | 77.6% | 0.088 | 0.1362 | 2000 |
| | 95.2% | 0.015 | 0.0286 | 2019 |
| **Mauritania** | 69.6% | 0.071 | 0.0983 | 2000 |
| | 80.0% | 0.047 | 0.0750 | 2019 |
| **Malawi** | 86.8% | 0.032 | 0.0555 | 2000 |
| | 96.4% | 0.004 | 0.0075 | 2019 |
| **Malaysia** | 86.5% | 0.034 | 0.0592 | 2000 |
| | 100.0% | 0.010 | 0.0210 | 2019 |
| **Namibia** | 83.0% | 0.043 | 0.0714 | 2000 |
| | 97.0% | 0.005 | 0.0102 | 2019 |
| **Niger** | 40.6% | 0.134 | 0.1086 | 2000 |
| | 62.8% | 0.054 | 0.0682 | 2019 |
| **Nigeria** | 39.3% | 0.339 | 0.2669 | 2000 |
| | 59.1% | 0.169 | 0.2000 | 2019 |
| **Nicaragua** | 93.1% | 0.010 | 0.0177 | 2000 |
| | 99.9% | 0.000 | 0.0006 | 2019 |
| **Nepal** | 78.9% | 0.076 | 0.1193 | 2000 |
| | 97.4% | 0.007 | 0.0146 | 2019 |
| **Pakistan** | 66.2% | 0.077 | 0.1021 | 2000 |
| | 76.2% | 0.099 | 0.1505 | 2019 |
| **Panama** | 94.3% | 0.003 | 0.0059 | 2000 |
| | 93.5% | 0.003 | 0.0060 | 2019 |
| **Peru** | 89.6% | 0.011 | 0.0205 | 2000 |
| | 86.0% | 0.019 | 0.0330 | 2019 |
| **Philippines** | 79.9% | 0.047 | 0.0751 | 2000 |
| | 76.6% | 0.035 | 0.0536 | 2019 |
| **Papua New Guinea** | 45.0% | 0.139 | 0.1254 | 2019 |
| | 87.1% | 0.021 | 0.0364 | 2000 |
| **Paraguay** | 89.7% | 0.011 | 0.0195 | 2000 |
| | 93.0% | 0.004 | 0.0071 | 2019 |
| **Rwanda** | 87.0% | 0.024 | 0.0413 | 2000 |
| | 91.6% | 0.012 | 0.0229 | 2019 |
| **Sudan** | 55.9% | 0.054 | 0.0609 | 2000 |
| | 84.9% | 0.083 | 0.1404 | 2019 |

| | | | | |
|---|---|---|---|---|
| **Senegal** | 69.1% | 0.068 | 0.0933 | 2000 |
| | 88.6% | 0.019 | 0.0338 | 2019 |
| **Sierra Leone** | 49.8% | 0.109 | 0.1091 | 2000 |
| | 89.6% | 0.021 | 0.0385 | 2019 |
| **El Salvador** | 86.3% | 0.007 | 0.0118 | 2000 |
| | 92.0% | 0.004 | 0.0070 | 2019 |
| **Somalia** | 23.9% | 0.136 | 0.0647 | 2019 |
| | 28.2% | 0.175 | 0.0985 | 2000 |
| **South Sudan** | 30.4% | 0.145 | 0.0880 | 2000 |
| | 46.3% | 0.077 | 0.0717 | 2019 |
| **Sao Tome and Principe** | 74.1% | 0.011 | 0.0170 | 2000 |
| | 99.4% | 0.000 | 0.0006 | 2019 |
| **Suriname** | 71.8% | 0.038 | 0.0548 | 2019 |
| | 77.9% | 0.029 | 0.0458 | 2000 |
| **Swaziland** | 86.2% | 0.012 | 0.0212 | 2000 |
| | 88.6% | 0.005 | 0.0091 | 2019 |
| **Syria** | 69.6% | 0.026 | 0.0366 | 2019 |
| | 77.7% | 0.012 | 0.0189 | 2000 |
| **Chad** | 26.8% | 0.212 | 0.1134 | 2000 |
| | 40.5% | 0.150 | 0.1216 | 2019 |
| **Togo** | 59.9% | 0.120 | 0.1437 | 2000 |
| | 76.5% | 0.037 | 0.0562 | 2019 |
| **Thailand** | 90.7% | 0.019 | 0.0339 | 2019 |
| | 95.0% | 0.008 | 0.0145 | 2000 |
| **Tajikistan** | 86.5% | 0.018 | 0.0319 | 2000 |
| | 90.2% | 0.013 | 0.0227 | 2019 |
| **Turkmenistan** | 94.4% | 0.004 | 0.0080 | 2019 |
| | 96.4% | 0.002 | 0.0037 | 2000 |
| **Timor-Leste** | 36.3% | 0.126 | 0.0915 | 2000 |
| | 67.7% | 0.042 | 0.0563 | 2019 |
| **Trinidad and Tobago** | 95.0% | 0.007 | 0.0137 | 2000 |
| | 93.6% | 0.004 | 0.0072 | 2019 |
| **Tunisia** | 92.9% | 0.007 | 0.0129 | 2000 |
| | 95.9% | 0.005 | 0.0086 | 2019 |
| **Tanzania** | 83.2% | 0.057 | 0.0947 | 2000 |
| | 88.1% | 0.031 | 0.0542 | 2019 |
| **Uganda** | 66.6% | 0.071 | 0.0939 | 2000 |
| | 72.2% | 0.045 | 0.0649 | 2019 |
| **Ukraine** | 93.7% | 0.012 | 0.0232 | 2000 |

| | | | | |
|---|---|---|---|---|
| | 100.0% | 0.000 | 0.0002 | 2019 |
| **Uzbekistan** | 92.2% | 0.005 | 0.0099 | 2019 |
| | 95.4% | 0.007 | 0.0128 | 2000 |
| **Vietnam** | 84.4% | 0.064 | 0.1087 | 2000 |
| | 90.3% | 0.021 | 0.0383 | 2019 |
| **Yemen** | 35.7% | 0.240 | 0.1714 | 2000 |
| | 70.8% | 0.054 | 0.0768 | 2019 |
| **South Africa** | 78.9% | 0.084 | 0.1320 | 2000 |
| | 78.2% | 0.019 | 0.0291 | 2019 |
| **Zambia** | 86.6% | 0.028 | 0.0480 | 2000 |
| | 90.5% | 0.017 | 0.0306 | 2019 |
| **Zimbabwe** | 71.6% | 0.039 | 0.0560 | 2000 |
| | 84.7% | 0.023 | 0.0392 | 2019 |

*Supplementary Table 14:* Age cohort regression coefficients

Coefficients from age cohort only regression analysis of the log of the ratios of coverage between older cohorts and the target cohort among cohorts of children with multiple survey timepoints. t-statistic and corresponding two-side p=values are presented. The t+3 cohort was significant.

| | Beta | Std. Error | t-value | p-value |
|---|---|---|---|---|
| T+1 | 0.01436 | 0.02483 | 0.578 | 0.5646 |
| T+2 | 0.05409 | 0.02786 | 1.941 | 0.0556 |
| T+3 | 0.14564 | 0.02840 | 5.129 | 0.00000184*** |

*Supplementary Table 15:* Key analysis findings for multiple age cohort modelling choices
For each modelling version with various cohorting approaches, key results and themes presented in the manuscript are consistent, including proportion of districts with increasing coverage and meeting GVAP targets, as well as inequality metrics and the geographic distribution of children.

| | Full cohort model | Target age, t+1 and t+2 cohort model*<br><br>*Presented as main findings | Target age and t+1 cohort model | Target age cohort only model |
|---|---|---|---|---|
| Proportion of districts with increased coverage from 2000 to 2019 | 58.0%<br>(95% UI: 51.1 – 65.6%) | 57.4%<br>(95% UI: 50.4–64.6%) | 55.9%<br>(95% UI: 49.3 – 63.8%) | 55.5%<br>(95% UI: 49.8 – 61.5%) |
| Proportion of districts with increased coverage from 2000 to 2010 | 69.6%<br>(95% UI: 64.9–74.4%) | 70.5% (95% UI: 66.0–75.4%) | 67.9%<br>(95% UI: 62.3 – 73.5%) | 72.0%<br>(95% UI: 67.6 – 76.3%) |
| Proportion of districts with increased coverage from 2010 to 2019 | 41.7%<br>(95% UI: 35.5 – 49.5%) | 40.1% (95% UI: 34.2–46.9%) | 40.3%<br>(95% UI: 34.5 – 47.6%) | 36.9%<br>(95% UI: 32.1 – 42.6%) |
| Proportion of districts meeting GVAP target in 2000 | 39.1% | 38.4% | 37.0% | 37.2% |
| Proportion of districts meeting GVAP target in 2019 | 34.0% | 33.2% | 33.1% | 34.8% |
| % of all unvaccinated children living in rural areas | 16.2% | 16.0% | 16.1% | 16.2% |
| % of all unvaccinated children living in urban areas | 47.5% | 47.9% | 48.0% | 47.6% |
| % of children living rural areas that are unvaccinated | 33.2% | 33.3% | 33.1% | 33.2% |
| % of all unvaccinated children living in urban areas | 14.9% | 15.2% | 15.1% | 14.9% |

| | | | | |
|---|---|---|---|---|
| Correlation between changes in coverage and absolute inequality from 2000 - 2019 | -0.49 (Pearson's product moment correlation, t = -5.65, p = 0.0000001564) | -0.47 (Pearson's product moment correlation, t = -5.36, p = 0.0000005332) | -0.45 (Pearson's product moment correlation, t = -4.95, p = 0.000003044) | -0.53 (Pearson's product moment correlation, t = -6.49, p =0.000000002564) |
| Number of countries with increasing inequality from 2000 - 2019 | 23 | 25 | 29 | 25 |

## 5.0 Supplementary References


1. Stevens, G. A. *et al.* Guidelines for accurate and transparent health estimates reporting: the GATHER statement. *The Lancet* **388**, e19–e23 (2016).
2. *WHO | WHO/UNICEF Joint Reporting Process*. http://www.who.int/immunization/monitoring_surveillance/routine/reporting/en/ (2019).
3. Dansereau, E., Brown, D., Stashko, L. & Danovaro-Holliday, M. C. A systematic review of the agreement of recall, home-based records, facility records, BCG scar, and serology for ascertaining vaccination status in low and middle-income countries. *Gates Open Res* **3**, 923 (2019).
4. Portnoy, A., Jit, M., Helleringer, S. & Verguet, S. Impact of measles supplementary immunization activities on reaching children missed by routine programs. *Vaccine* **36**, 170–178 (2018).
5. World Health Organization. COVID-19: Operational guidance for maintaining essential health services during an outbreak. https://www.who.int/publications-detail/covid-19-operational-guidance-for-maintaining-essential-health-services-during-an-outbreak.
6. World Health Organization. *Global measles and rubella strategic plan: 2012-2020.* http://apps.who.int/iris/bitstream/10665/44855/1/9789241503396%5Feng.pdf (2012).
7. Utazi, C. E. *et al.* High resolution age-structured mapping of childhood vaccination coverage in low and middle income countries. *Vaccine* **36**, 1583–1591 (2018).
8. Vos, T. *et al.* Global burden of 369 diseases and injuries in 204 countries and territories, 1990–2019: a systematic analysis for the Global Burden of Disease Study 2019. *The Lancet* **396**, 1204–1222 (2020).
9. WHO | Decade of Vaccines — Global Vaccine Action Plan 2011-2020. *WHO* https://www.who.int/immunization/global_vaccine_action_plan/DoV_GVAP_2012_2020/en/.
10. Dicker, D. *et al.* Global, regional, and national age-sex-specific mortality and life expectancy, 1950–2017: a systematic analysis for the Global Burden of Disease Study 2017. *Lancet* **392**, 1684–1735 (2018).

11. Global Health Data Exchange | GHDx. http://ghdx.healthdata.org/ (2019).

12. World Health Organization. Harmonizing vaccination coverage measures in household surveys: A primer. https://www.who.int/immunization/monitoring_surveillance/Surveys_White_Paper_immunization_2019.pdf (2019).

13. WHO | Data, statistics and graphics. *WHO* http://www.who.int/immunization/monitoring_surveillance/data/en/.

14. Mosser, J. F. *et al.* Mapping diphtheria-pertussis-tetanus vaccine coverage in Africa, 2000–2016: a spatial and temporal modelling study. *Lancet* **393**, 1843–1855 (2019).

15. Tatem, A. J. WorldPop, open data for spatial demography. *Sci Data* **4**, 170004 (2017).

16. Package 'survey'. Lumley, T. https://cran.r-project.org/web/packages/survey/survey.pdf.

17. Faraway, J. J. *Linear Models with R*.

18. Bhatt, S. *et al.* Improved prediction accuracy for disease risk mapping using Gaussian process stacked generalization. *Journal of The Royal Society Interface* **14**, 20170520 (2017).

19. Fuglstad, G.-A., Simpson, D., Lindgren, F. & Rue, H. Constructing priors that penalize the complexity of Gaussian random fields. *Journal of the American Statistical Association* **114**, 445–452 (2019).

20. Gómez-Rubio, V. *Chapter 6 Advanced Features | Bayesian inference with INLA*.

21. Fullman, N. *et al.* Measuring performance on the Healthcare Access and Quality Index for 195 countries and territories and selected subnational locations: a systematic analysis from the Global Burden of Disease Study 2016. *The Lancet* **391**, 2236–2271 (2018).

22. Python 2.7.0 Release. *Python.org* https://www.python.org/download/releases/2.7/.

23. ArcMap | Documentation. https://desktop.arcgis.com/en/arcmap/.

24. The Comprehensive R Archive Network. https://cran.r-project.org/.

25. The Coffey-Feingold-Bromberg measure: A normed measure of variability among proportions.

26. ESA-CCI Project. Land cover classification gridded maps from 1992 to present derived from satellite observations. https://cds.climate.copernicus.eu/cdsapp#!/dataset/satellite-land-cover?tab=overview (2020).

27. World Health Organization. Immunization schedules by diseases. https://apps.who.int/immunization_monitoring/globalsummary/diseases (2019).
