# Supplementary material for: Mapping routine measles vaccination in low- and middle-income countries
Source: Nature. 2020 Dec 16;589(7842):415–9. doi: 10.1038/s41586-020-03043-4 (PMC7739806; doi:10.1038/s41586-020-03043-4)
Supplement: Supplementary file 2 — Reporting Summary [file 41586_2020_3043_MOESM2_ESM.pdf]

nature research

Corresponding author(s): Jonathan F. Mosser

Last updated by author(s): Dec 3, 2020

Reporting Summary

Nature Research wishes to improve the reproducibility of the work that we publish. This form provides structure for consistency and transparency in reporting. For further information on Nature Research policies, see Authors & Referees and the Editorial Policy Checklist.

Statistics

For all statistical analyses, confirm that the following items are present in the figure legend, table legend, main text, or Methods section.

n/a	Confirmed	
☐	☒	The exact sample size (*n*) for each experimental group/condition, given as a discrete number and unit of measurement
☐	☒	A statement on whether measurements were taken from distinct samples or whether the same sample was measured repeatedly
☐	☒	The statistical test(s) used AND whether they are one- or two-sided<br>*Only common tests should be described solely by name; describe more complex techniques in the Methods section.*
☐	☒	A description of all covariates tested
☐	☒	A description of any assumptions or corrections, such as tests of normality and adjustment for multiple comparisons
☐	☒	A full description of the statistical parameters including central tendency (e.g. means) or other basic estimates (e.g. regression coefficient) AND variation (e.g. standard deviation) or associated estimates of uncertainty (e.g. confidence intervals)
☐	☒	For null hypothesis testing, the test statistic (e.g. *F*, *t*, *r*) with confidence intervals, effect sizes, degrees of freedom and *P* value noted<br>*Give P values as exact values whenever suitable.*
☐	☒	For Bayesian analysis, information on the choice of priors and Markov chain Monte Carlo settings
☒	☐	For hierarchical and complex designs, identification of the appropriate level for tests and full reporting of outcomes
☒	☐	Estimates of effect sizes (e.g. Cohen's *d*, Pearson's *r*), indicating how they were calculated

Our web collection on statistics for biologists contains articles on many of the points above.

Software and code

Policy information about availability of computer code

Data collection	No primary data collection was carried out for this analysis.
Data analysis	This analysis was carried out using R version 3.6.1 and using R-INLA v.20.01.29.9000. Maps were produced using ArcGIS Desktop 10.6 and Python 2.7. All code used for these analyses will be made publicly available upon publication.

For manuscripts utilizing custom algorithms or software that are central to the research but not yet described in published literature, software must be made available to editors/reviewers. We strongly encourage code deposition in a community repository (e.g. GitHub). See the Nature Research guidelines for submitting code & software for further information.

Data

Policy information about availability of data

All manuscripts must include a data availability statement. This statement should provide the following information, where applicable:

- Accession codes, unique identifiers, or web links for publicly available datasets
- A list of figures that have associated raw data
- A description of any restrictions on data availability

The findings of this study are supported by data available in public online repositories and data publicly available upon request of the data provider. A detailed table of data sources and availability can be found in Supplementary Table 4 and http://ghdx.healthdata.org/lbd-publication-data-input-sources. Administrative boundaries were modified from the Database for Global Administrative Areas (GADM) dataset. Populations were retrieved from WorldPop and gridded estimates of travel time to nearest city or settlement were available online from work by Weiss, et al 2018. This study complies with the Guidelines for Accurate and Transparent Health Estimates Reporting (GATHER) recommendations. All maps and figures presented in this study are generated by the authors; no permissions are required for publication.

Field-specific reporting

Please select the one below that is the best fit for your research. If you are not sure, read the appropriate sections before making your selection.

☒ Life sciences ☐ Behavioural & social sciences ☐ Ecological, evolutionary & environmental sciences

For a reference copy of the document with all sections, see nature.com/documents/nr-reporting-summary-flat.pdf

Life sciences study design

All studies must disclose on these points even when the disclosure is negative.

Sample size	This observational study incorporated all available survey data sources that met the inclusion criteria as described in detail in the manuscript and supplementary information. The combined dataset from 354 household based surveys contained information on vaccination status from 1.70 million individual children.
Data exclusions	Surveys were excluded due to unrealistic national or geographic trends compared to other surveys in nearby country-years, inability to match the microdata to geographic locations, or non-standard methodology. These criteria were pre-established prior to reviewing the data. A full list of excluded surveys is included in Supplementary Table 5.
Replication	All code and data are available publicly for reproducibility.
Randomization	As this work is an observational mapping study, there were no experimental groups.
Blinding	As this work is an observational mapping study, there was no need for blinding.

Reporting for specific materials, systems and methods

We require information from authors about some types of materials, experimental systems and methods used in many studies. Here, indicate whether each material, system or method listed is relevant to your study. If you are not sure if a list item applies to your research, read the appropriate section before selecting a response.

Materials & experimental systems

n/a	Involved in the study	
☒	☐	Antibodies
☒	☐	Eukaryotic cell lines
☒	☐	Palaeontology
☒	☐	Animals and other organisms
☒	☐	Human research participants
☒	☐	Clinical data

Methods

n/a	Involved in the study	
☒	☐	ChIP-seq
☒	☐	Flow cytometry
☒	☐	MRI-based neuroimaging
